# Key Genetic Components of Fibrosis in Diabetic Nephropathy: An Updated Systematic Review and Meta-Analysis

**DOI:** 10.3390/ijms232315331

**Published:** 2022-12-05

**Authors:** Maria Tziastoudi, Theoharis C. Theoharides, Evdokia Nikolaou, Maria Efthymiadi, Theodoros Eleftheriadis, Ioannis Stefanidis

**Affiliations:** 1 Department of Nephrology, Faculty of Medicine, School of Health Sciences, University of Thessaly, 41110 Larissa, Greece; 2 Laboratory of Molecular Immunopharmacology and Drug Discovery, Department of Immunology, Tufts University School of Medicine, Boston, MA 02155, USA; 3 School of Graduate Biomedical Sciences, Tufts University School of Medicine, Boston, MA 02155, USA; 4 Departments of Internal Medicine and Psychiatry, Tufts University School of Medicine and Tufts Medical Center, Boston, MA 02155, USA; 5 Institute of Neuro-Immune Medicine, Nova Southeastern University, Clearwater, FL 33314, USA

**Keywords:** renal fibrosis, diabetic nephropathy, genes, review, meta-analysis

## Abstract

Renal fibrosis (RF) constitutes the common end-point of all kinds of chronic kidney disease (CKD), regardless of the initial cause of disease. The aim of the present study was to identify the key players of fibrosis in the context of diabetic nephropathy (DN). A systematic review and meta-analysis of all available genetic association studies regarding the genes that are included in signaling pathways related to RF were performed. The evaluated studies were published in English and they were included in PubMed and the GWAS Catalog. After an extensive literature review and search of the Kyoto Encyclopedia of Genes and Genomes (KEGG) database, eight signaling pathways related to RF were selected and all available genetic association studies of these genes were meta-analyzed. *ACE*, *AGT*, *EDN1*, *EPO*, *FLT4*, *GREM1*, *IL1B*, *IL6*, *IL10*, *IL12RB1*, *NOS3*, *TGFB1*, *IGF2/INS/TH cluster*, and *VEGFA* were highlighted as the key genetic components driving the fibrosis process in DN. The present systematic review and meta-analysis indicate, as key players of fibrosis in DN, sixteen genes. However, the results should be interpreted with caution because the number of studies was relatively small.

## 1. Introduction

Diabetic nephropathy (DN) is a multifactorial condition that involves both metabolic and hemodynamic factors, but clear evidence (heritability estimates, familial clustering of the disease, and differential vulnerability according to race) also indicates that the genetic profile of individuals plays a major role [1,2,3]. In addition, differences in the risk of DN among ethnic populations indicate that differences in lifestyle and environment also play major roles in the selection of alleles contributing to the different risks of disease occurrence [4]. These observations suggest that some diabetic patients are programmed to develop DN. Given the continuous increase in both DM and its complications, the effort to prevent DM is still considered a global challenge.

Among the histological lesions that occur in DN [5,6], renal fibrosis (RF) is considered as a complex and irreversible process in the late stages of DN, further exacerbating the progression of the disease [6]. In RF, which takes place in response to injury and inflammation, there is excessive deposition of the extracellular matrix (ECM) and epithelial–mesenchymal transition (EMT), leading to the loss of differentiated epithelial cells and their vascular capillary bed, accumulation of myofibroblasts and inflammatory cells, and, ultimately, the formation of a scar [7]. As a result, the destruction of the normal architecture and function of the kidney occurs [6,8,9]. RF also constitutes the end point of all kinds of chronic kidney disease (CKD), regardless of the initial cause of disease [8].

It is noteworthy that, although fibrosis is considered a detrimental condition, recent studies suggest a protective role of this process, because it helps the maintenance of crosstalk with injured proximal tubular cells supporting their regeneration [10]. In autosomal-dominant polycystic kidney disease (ADPKD), for instance, fibrosis may have some protective roles, as the fibrogenic response is generally correlated with cystic disease regression [11].

Given the undoubtedly genetic involvement in the course of DN, many genetic studies, such as linkage scans and genetic association studies (GAS), as well as meta-analyses of these studies, have been published [12,13,14,15,16]. A major contribution to the genetic dissection of DN has been made by genome-wide association studies (GWAS) and their meta-analyses [17,18,19,20,21,22].

In order to identify the key players of fibrosis in the context of DN, we performed a systematic review and meta-analysis of all available genetic association studies regarding genes that are involved in signaling pathways related to RF. In this study, we included all available genetic association studies regarding fibrosis-related genes that have been published within a time period of 35 months since our field synopsis of all available genetic association studies regarding DN [13] and presented an updated meta-analysis of the relevant polymorphisms.

## 2. Methods

### 2.1. Identification and Eligibility of Relevant Studies

**Figure 1:**
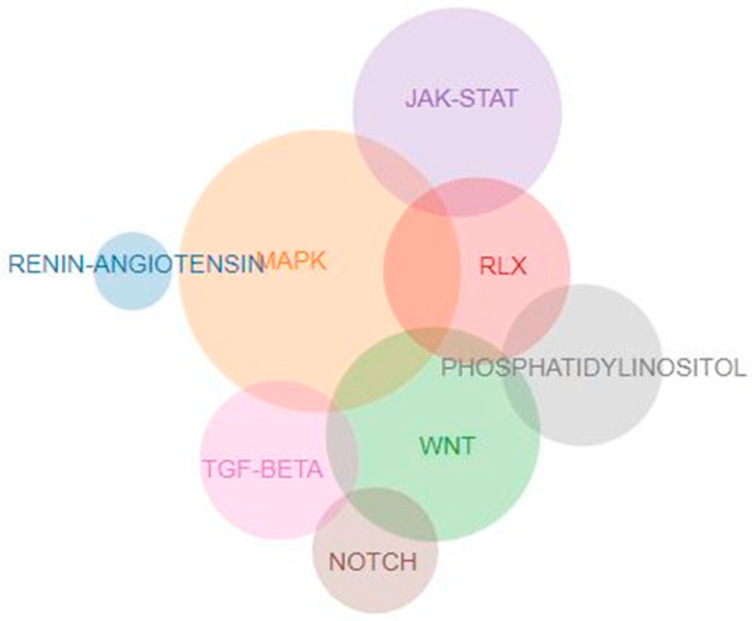
Venn diagram showing the overlap between genes in pathways.

In an effort to decipher the key genetic components of the RF process, we performed a systematic review and meta-analysis of eight signaling pathways that are closely related to RF. The eight pathways were selected after a literature review and after a search of the Kyoto Encyclopedia of Genes and Genomes (KEGG) database. The included pathways were the ACE pathway, the Relaxin pathway, the Wnt signaling pathway, the MAPK signaling pathway, the PI3KC signaling pathway, the TGFB1 signaling pathway, the NOTCH signaling pathway, and the JAK signaling pathway (Table 1). The overlap between genes in these pathways is depicted in a Venn diagram (Figure 1). The full names of the genes in each signaling pathway are shown in Supplementary Tables S1–S8. In the meta-analysis, we included GAS that examined the association of any gene included in the aforementioned pathways with DN.

A literature search of PubMed and the GWAS Catalog (http://www.genome.gov/gwastudies/) (accessed on 11 June 2022) was conducted, and the inclusion and exclusion criteria and data extraction were as previously described [13]. More specifically, cases were defined as diabetics with persistent micro/macroalbuminuria with or without diabetic retinopathy, whereas diseased controls were defined as diabetics with normoalbuminuria and/or normal renal function. The eligibility of the studies was assessed by two investigators (M.T. and I.S.). The reporting of the systematic review process will follow the PRISMA statement [23].

### 2.2. Data Synthesis and Analysis

The genetic association between each polymorphism and DN was assessed using the generalized odds ratio (ORG) [24,25]. The threshold for the meta-analysis was the presence of two studies per genetic polymorphism. The pooled OR was estimated using the Der Simonian and Laird random-effects model [26]. The associations are presented with ORs and their corresponding 95% confidence intervals (Cis). The between-study heterogeneity was tested with Cochran’s Q statistic (considered statistically significant at *p* < 0.10) and its extent was assessed with the I^2^ statistic [27,28].

We also examined if controls confronted with Hardy–Weinberg equilibrium (HWE) predicted genotypes using Fisher’s exact test for each study that provided genotype counts. We also tested for the ‘small-study effect’ with the Egger test [29].

## 3. Results and Discussion

### 3.1. Study Characteristics

**Figure 2:**
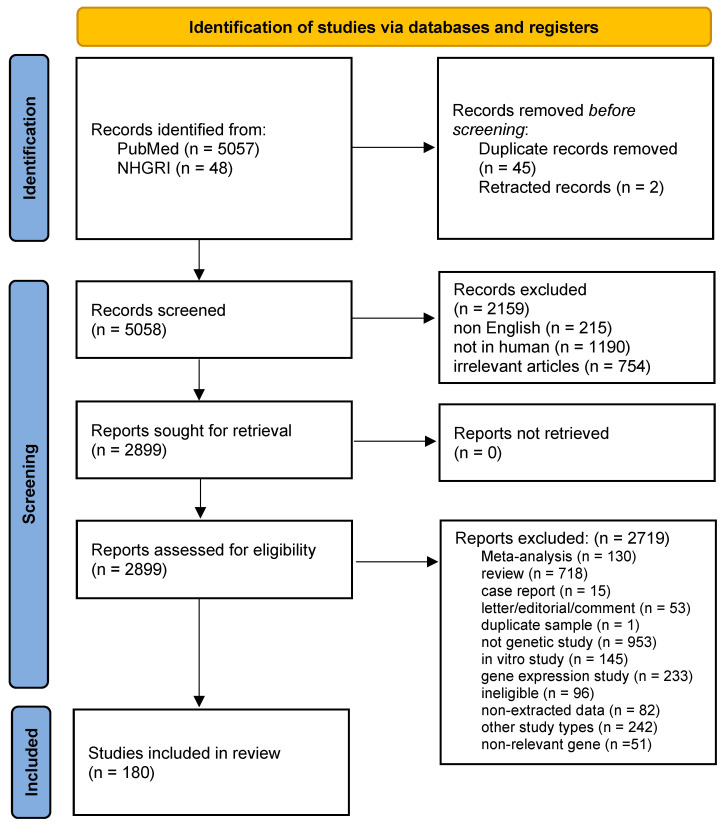
Flowchart of retrieved articles with specification of the reasons for exclusion.

The literature search of both PubMed and the GWAS Catalog retrieved 5058 papers after the exclusion of duplicate studies, whereas, in the meta-analysis, 180 articles were included. When an article provided data for different populations, each population was considered as a different study. Figure 2 presents a flowchart of the retrieved articles and the reasons for the exclusion of certain papers. The included studies were published between 1994 and 2021. The demographic characteristics of each study are shown in Supplementary Table S9, which was updated from our previous study [13]. Supplementary Table S9 only presents the included studies of these polymorphisms that were updated from our previous study [13].

### 3.2. Meta-Analysis Results

**Figure 3:**
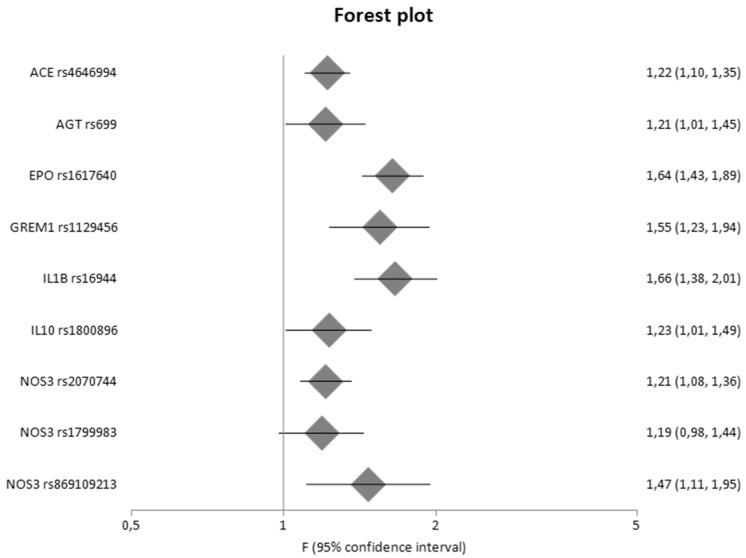
Meta-analysis results of diseased controls versus cases based on genotype counts.

**Figure 4:**
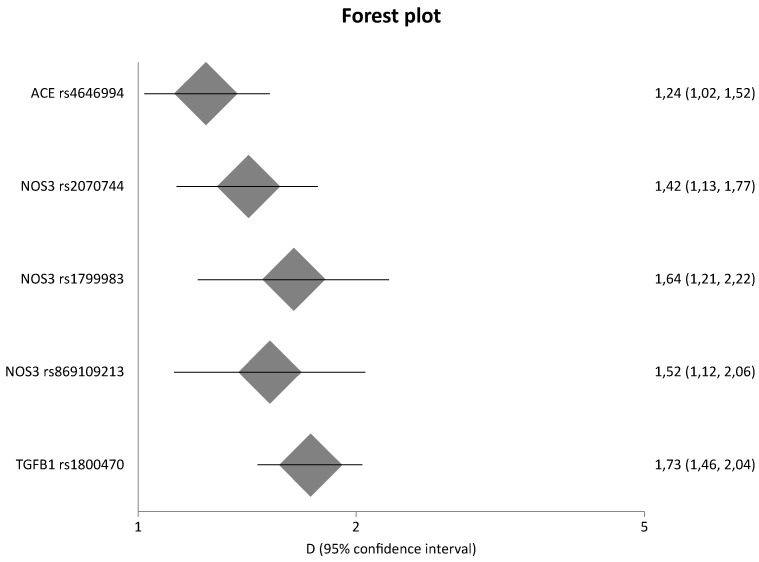
Meta-analysis results of healthy controls versus cases based on genotype counts.

Among the 884 different genes that were involved in eight pathways related to fibrosis, 134 genetic variants located in 45 different genes were meta-analyzed. In comparison with our previous study [13], the present study updated results for 24 genes. Table 2 shows the statistically significant results of the meta-analyses based on genotype counts, whereas Table 3 shows the statistically significant results of the meta-analyses based on allele counts. Table 2 and Table 3 include also updated data from meta-analyses that were already published by our group in a previous study [13]. However, for the purpose of completeness, Supplementary Tables S10–S12 also present the non-significant results. Figure 3 and Figure 4 are forest plots that show the pooled odds ratios of the significant results. Overall, sixteen genes provided significant results in all meta-analyses.

In meta-analyses based on genotype counts, statistically significant results were reported for angiotensin I-converting enzyme (*ACE*), angiotensinogen (*AGT*), erythropoietin (*EPO*), gremlin 1, DAN family BMP antagonist (*GREM1*), interleukin 1 beta (*IL1B*), interleukin 6 (*IL6*), interleukin 10 (*IL10*), nitric oxide synthase 3 (*NOS3*), and transforming growth factor beta 1 (*TGFB1*). 

More specifically, in comparison with diseased controls versus cases, the *ACE* I/D polymorphism was significantly associated with DN with a pooled OR_G_ of 1.22 (95% CI 1.10–1.35), the *AGT* M235T variant showed significant results with a pooled OR_G_ of 1.21 (95% CI 1.01–1.45), *EPO* rs1617640 was significantly associated with DN with a pooled OR_G_ of 1.64 (95% CI 1.43–1.89), *GREM1* rs1129456 was associated with DN with a pooled OR_G_ of 1.55 (95% CI 1.23–1.94), *IL1B* -511C > T provided significant results with a pooled OR_G_ of 1.66 (95% CI 1.38–2.01), *IL10* -1082A > G was also associated with DN with a pooled OR_G_ of 1.23 (95% CI 1.01–1.49), and three variants in *NOS3* gene (rs2070744, rs1799983, rs869109213) produced significant results with pooled OR_G_ values of 1.21 (95% CI 1.08–1.36), 1.19 (95% CI 0.98–1.44), and 1.47 (1.11–1.95), respectively. 

When healthy controls were compared with cases, the *ACE* I/D polymorphism was significantly associated with DN with a pooled OR_G_ of 1.24 (95% CI 1.02–1.52), three variants of the *NOS3* gene (rs2070744, rs1799983, and rs869109213) provided significant results with pooled OR_G_ values of 1.42 (95% CI 1.13–1.77), 1.64 (95% CI 1.21–2.22), 1.52 (95% CI 1.12–2.06), respectively, and TGFB1 T869C was also associated with DN with a pooled OR_G_ value of 1.73 (95% CI 1.46–2.04).

In a comparison of healthy controls versus diseased controls versus cases, *IL6* rs1800795 was significantly associated with DN with a pooled OR_G_ of 1.44 (95% CI 1.10–1.89), and three variants of the *NOS3* gene (rs2070744, rs1799983, rs869109213) gave significant results, with pooled OR_G_ values of 1.29 (95% CI 1.17–1.43), 1.28 (95% CI 1.05–1.56), and 1.30 (95% CI 1.04–1.63), respectively.

In meta-analyses based on allele counts, significant associations were reported for endothelin 1 (*EDN1*), fms-related receptor tyrosine kinase 4 (*FLT4*), insulin-like growth factor 2/insulin/tyrosine hydroxylase cluster (*IGF2/INS/TH cluster*), interleukin 12 receptor subunit beta 1 (*IL12RB1*), and vascular endothelial growth factor A (*VEGFA*).

**Figure 5:**
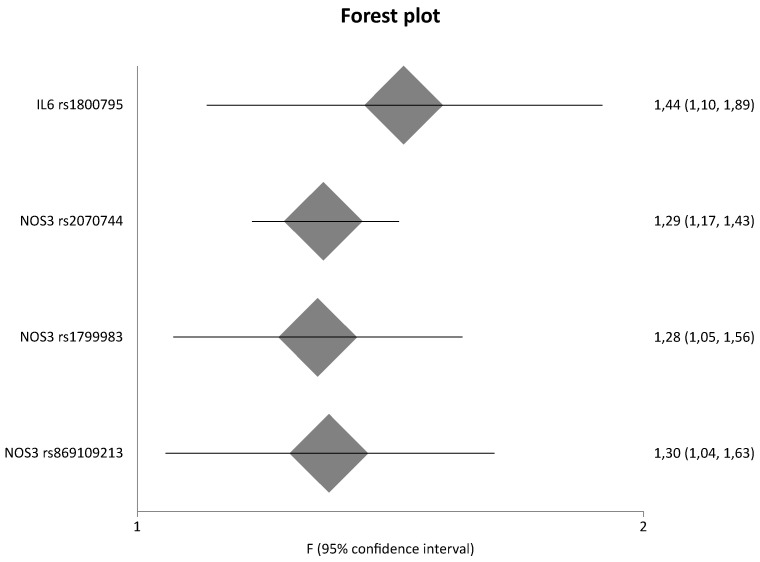
Meta-analysis results of healthy controls versus diseased controls versus cases based on genotype counts.

**Figure 6:**
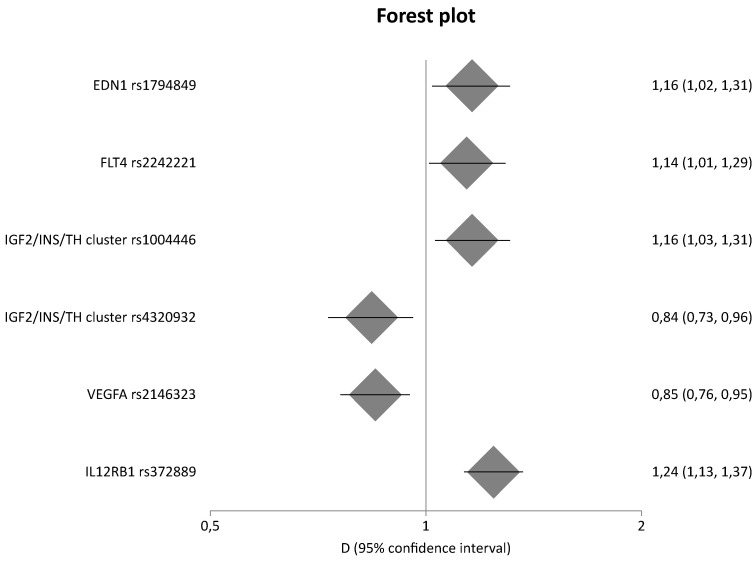
Meta-analysis results of diseased controls versus cases and healthy controls versus cases based on allele counts.

More specifically, in a comparison of diseased controls versus cases, *EDN1* rs1794849 was significantly associated with DN, with a pooled OR of 1.16 (95% CI 1.02–1.31), *FLT4* rs2242221 produced significant results with a pooled OR of 1.14 (95% CI 1.01–1.29), two variants in the IGF2/INS/TH cluster, rs1004446 and rs4320932, were also associated with DN, with pooled OR values of 1.16 (95% CI 1.03–1.31) and 0.84 (95% CI 0.73–0.96), respectively, and *VEGFA* rs2146323 also produced significant results, with a pooled OR of 0.85 (95% CI 0.76–0.95). In a comparison of healthy controls versus cases, only one variant in IL12RB1 (rs372889) was associated with DN, with a pooled OR value of 1.24 (95% CI 1.13–1.37). Figure 3, Figure 4, Figure 5 and Figure 6 are forest plot representations of the genetic variants that are significantly associated with DN.

### 3.3. Discussion

To the best of our knowledge, this review constitutes the most comprehensive study of the genetic variants that are related to fibrosis in the context of DN. This study is an extension of our previous work in the field of the genetic epidemiology of DN [13], but, for the first time, we focused on fibrosis-related genes. Among the 134 genetic variants located in 45 different genes that were meta-analyzed, sixteen genes (*ACE*, *AGT*, *EDN1*, *EPO*, *FLT4*, *GREM1*, *IL1B*, *IL6*, *IL10*, *IL12RB1*, *NOS3*, *TGFB1*, *IGF2/INS/TH cluster*, and *VEGFA*) produced significant results in all meta-analyses.

The present systematic review confirmed the statistical significance of genes that are well known to have fibrotic effects, such as *ACE*, *AGT*, *EDN1*, *GREM1*, *IL1B*, *IL6*, and *TGFB1*, whereas some other genes are known for their anti-fibrotic effects, such as *EPO* and *NOS3*. The statistically significant contribution of *IL12RB1* and *TH* constitutes a novel finding, as there are no available experimental results to clarify their contribution to fibrosis.

More specifically, *ACE* and *AGT* constitute members of the renin–angiotensin system (RAS), but this finding is no surprise because RAS inhibitors also have antifibrotic effects [30]. Many lines of evidence indicate that RAS is a major regulator of renal fibrosis, as angiotensin II (AngII) promotes the release of TGF-β and also activates the inflammatory process [31]. In addition, many publications indicate the involvement of TGFB1, which has been also characterized as the master regulator of fibrosis via the activation of both canonical and non-canonical pathways [32]. TGF-β is also regarded as the most important inducer of endothelial-to-mesenchymal transition (EndMT) in vitro and in vivo, a form of EMT [33], suggesting that targeting the TGF-β receptor signaling pathway could constitute a putative treatment for fibrosis [33].

Regarding the role of the innate proinflammatory cytokine IL1B, it has been reported that it induces a metabolic switch from oxidative phosphorylation to glycolysis in kidney stromal cells (SCs), promoting proximal tubule damage and fibrosis [34]. Increased expression of IL-6 and extensive and chronic activation of STAT3 were also associated with fibrosis [35]. The pathways of the anti-inflammatory mediator IL-10 have been found to be conserved in many diseases associated with fibrosis, although their underlying dissimilarity suggests that the IL-10 signaling pathway may have antifibrotic properties [36]. With regard to *IL12RB1*, to the best of our knowledge, this is a novel finding as no previous studies have found any association of *IL12RB1* with kidney fibrosis.

A previous study reported that the disruption of *eNOS* and *ApoE* genes accelerates kidney fibrosis and senescence after injury [37], indicating a protective role of the normal function of these genes. In addition, a meta-analysis that investigated the association between the (*eNOS*) 4b/a gene polymorphism and renal interstitial fibrosis in patients with DN demonstrated that the frequency of *eNOS*4bb in DN renal interstitial patients was lower than that in non-nephropathy diabetic patients and normal controls [38]. Regarding *EDN1*, it has been found that the endothelin receptors in renal interstitial cells do not contribute to the development of fibrosis during experimental kidney disease [39]. However, a randomized controlled trial, SONAR, regarding the selective endothelin A receptor antagonist atrasentan showed promising results, as atrasentan reduced the risk of renal events in patients with diabetes and chronic kidney disease [40]. Regarding the *EPO* gene, it has been found that erythropoietin attenuates renal interstitial fibrosis via the inhibition of fibrocyte accumulation [41]. Gremlin1 (*Grem1*), which is an antagonist of bone morphogenetic proteins, plays a key role in kidney development and renal fibrosis, and a previous study demonstrated that its levels are increased in many diseases associated with fibrosis [42]. More specifically, the grem1 levels are increased in renal fibrosis, as well as in fibrosis of the heart and lungs. *FLT4*, a tyrosine kinase receptor for *VEGFC* and *VEGFD*, is involved in lymphangiogenesis, which is a condition that develops during the progression of fibrosis, indicating a fibrotic effect of this factor [43].

Regarding the *IGF2*/*INS*/*TH* cluster, previous results have shown that IGF2 stimulates the differentiation into myofibroblasts that produce large amounts of collagen and other extracellular matrix proteins (ECM) [44]. Regarding *INS*, a hormone that plays a key role in carbohydrate and lipid metabolism, it has been found that sodium–glucose cotransporter 2 (SGLT2) inhibitors suppressed kidney fibrosis in diabetic mice [45]. Regarding *TH*, which is involved in tyrosine-to-dopamine conversion, there are no results regarding its involvement in kidney fibrosis. Finally, *VEGFA*, a growth factor essential for both physiological and pathological angiogenesis, can inhibit the expression of Smad3 and miR192, thereby suppressing TGF-β-induced EMT and improving renal fibrosis [46].

Based on the statistically significant results of the present systematic review and meta-analysis, it should be noted that mast cells (MCs) are implicated in many fibrotic conditions [47,48,49,50]. *ACE*, *IL1B*, *IL-6*, and *IL-10* are key mediators of MCs, and these genes have produced statistically significant results in the present systematic review and meta-analysis. The role of MCs in the fibrotic process is controversial, as studies in humans and in vitro data indicate a pro-fibrotic role of these cells, whereas animal studies have produced inconsistent results [51]. The reason may be the duration of the stimuli. Based on the fact that MCs are scarce in healthy human kidneys and are very rarely observed in glomeruli [52], these cells could serve as sensors of injury and could enhance the repair process. When the injury is short-lived, the MCs have an anti-fibrotic effect, but when the injury is chronic or repeated, the MCs have a pro-fibrotic effect [51]. It has been also found that an increase in the number of MCs is correlated negatively with renal function [53,54], but is correlated positively with the extent of fibrosis [54,55,56]. One more reason for the inconsistency is the fact that MCs produce both pro-fibrotic and anti-fibrotic mediators [49,57,58,59,60].

## 4. Conclusions

In summary, the present systematic review and meta-analysis indicate, as key players of fibrosis in DN, sixteen genes (*ACE*, *AGT*, *EDN1*, *EPO*, *FLT4*, *GREM1*, *IL1B*, *IL6*, *IL10*, *IL12RB1*, *NOS3*, *TGFB1*, *IGF2/INS/TH cluster*, and *VEGFA*). However, the results should be interpreted with caution because the number of studies in most meta-analyses is relatively small.

## Figures and Tables

**Table 1:** Pathways related to renal fibrosis in KEGG *.

**ACE pathway** *ACE*, *ACE2*, *AGT*, *AGTR1*, *AGTR2*, *ANPEP*, *ATP6AP2*, *CMA1*, *CPA3*, *CTSA*, *CTSG*, *ENPEP*, *KLK1*, *KLK2*, *LNPEP*, *MAS1*, *MME*, *MRGPRD*, *NLN*, *PRCP*, *PREP*, *REN*, *THOP1*
**Relaxin pathway** *ACTA2*, *ADCY1*, *ADCY2*, *ADCY3*, *ADCY4*, *ADCY5*, *ADCY6*, *ADCY7*, *ADCY8*, *ADCY9*, *AKT1*, *AKT2*, *AKT3*, *ARRB1*, *ARRB2*, *ATF2*, *ATF4*, *ATF6B*, *COL1A1*, *COL1A2*, *COL3A1*, *COL4A1*, *COL4A2*, *COL4A3*, *COL4A4*, *COL4A5*, *COL4A6*, *CREB1*, *CREB3*, *CREB3L1*, *CREB3L2*, *CREB3L3*, *CREB3L4*, *CREB5*, *EDN1*, *EDNRB*, *EGFR*, *FOS*, *GNA15*, *GNAI1*, *GNAI2*, *GNAI3*, *GNAO1*, *GNAS*, *GNB1*, *GNB2*, *GNB3*, *GNB4*, *GNB5*, *GNG10*, *GNG11*, *GNG12*, *GNG13*, *GNG2*, *GNG3*, *GNG4*, *GNG5*, *GNG7*, *GNG8*, *GNGT1*, *GNGT2*, *GRB2*, *HRAS*, *INSL3*, *INSL5*, *JUN*, *KRAS*, *MAP2K1*, *MAP2K2*, *MAP2K4*, *MAP2K7*, *MAPK1*, *MAPK10*, *MAPK11*, *MAPK12*, *MAPK13*, *MAPK14*, *MAPK3*, *MAPK8*, *MAPK9*, *MMP1*, *MMP13*, *MMP2*, *MMP9*, *NFKB1*, *NFKBIA*, *NOS1*, *NOS2*, *NOS3*, *NRAS*, *PIK3CA*, *PIK3CB*, *PIK3CD*, *PIK3R1*, *PIK3R2*, *PIK3R3*, *PLCB1*, *PLCB2*, *PLCB3*, *PLCB4*, *PRKACA*, *PRKACB*, *PRKACG*, *PRKCA*, *PRKCZ*, *RAF1*, *RELA*, *RLN1*, *RLN2*, *RLN3*, *RXFP1*, *RXFP2*, *RXFP3*, *RXFP4*, *SHC1*, *SHC2*, *SHC3*, *SHC4*, *SMAD2*, *SOS1*, *SOS2*, *SRC*, *TGFB1*, *TGFBR1*, *TGFBR2*, *VEGFA*, *VEGFB*, *VEGFC*, *VEGFD*
**Wnt signaling pathway** *APC*, *APC2*, *APCDD1*, *APCDD1L*, *AXIN1*, *AXIN2*, *BAMBI*, *BTRC*, *CACYBP*, *CAMK2A*, *CAMK2B*, *CAMK2D*, *CAMK2G*, *CBY1*, *CCDC88C*, *CCN4*, *CCND1*, *CCND2*, *CCND3*, *CER1*, *CHD8*, *CREBBP*, *CSNK1A1*, *CSNK1A1L*, *CSNK1E*, *CSNK2A1*, *CSNK2A2*, *CSNK2A3*, *CSNK2B*, *CTBP1*, *CTBP2*, *CTNNB1*, *CTNNBIP1*, *CTNND2*, *CUL1*, *CXXC4*, *DAAM1*, *DAAM2*, *DKK1*, *DKK2*, *DKK4*, *DVL1*, *DVL2*, *DVL3*, *EP300*, *FBXW11*, *FOSL1*, *FRAT1*, *FRAT2*, *FRZB*, *FZD1*, *FZD10*, *FZD2*, *FZD3*, *FZD4*, *FZD5*, *FZD6*, *FZD7*, *FZD8*, *FZD9*, *GPC4*, *GSK3B*, *INVS*, *JUN*, *LEF1*, *LGR4*, *LGR5*, *LGR6*, *LRP5*, *LRP6*, *MAP3K7*, *MAPK10*, *MAPK8*, *MAPK9*, *MMP7*, *MYC*, *NFATC1*, *NFATC2*, *NFATC3*, *NFATC4*, *NKD1*, *NKD2*, *NLK*, *NOTUM*, *PLCB1*, *PLCB2*, *PLCB3*, *PLCB4*, *PORCN*, *PPARD*, *PPP3CA*, *PPP3CB*, *PPP3CC*, *PPP3R1*, *PPP3R2*, *PRICKLE1*, *PRICKLE2*, *PRICKLE3*, *PRICKLE4*, *PRKACA*, *PRKACB*, *PRKACG*, *PRKCA*, *PRKCB*, *PRKCG*, *PSEN1*, *RAC1*, *RAC2*, *RAC3*, *RBX1*, *RHOA*, *RNF43*, *ROCK2*, *ROR1*, *ROR2*, *RSPO1*, *RSPO2*, *RSPO3*, *RSPO4*, *RUVBL1*, *RYK*, *SENP2*, *SERPINF1*, *SFRP1*, *SFRP2*, *SFRP4*, *SFRP5*, *SIAH1*, *SKP1*, *SMAD3*, *SMAD4*, *SOST*, *SOX17*, *TBL1X*, *TBL1XR1*, *TBL1Y*, *TCF7*, *TCF7L1*, *TCF7L2*, *TLE1*, *TLE2*, *TLE3*, *TLE4*, *TLE6*, *TLE7*, *TP53*, *TPTEP2-CSNK1E*, *VANGL1*, *VANGL2*, *WIF1*, *WNT1*, *WNT10A*, *WNT10B*, *WNT11*, *WNT16*, *WNT2*, *WNT2B*, *WNT3*, *WNT3A*, *WNT4*, *WNT5A*, *WNT5B*, *WNT6*, *WNT7A*, *WNT7B*, *WNT8A*, *WNT8B*, *WNT9A*, *WNT9B*, *ZNRF3*
**MAPK signaling pathway** *AKT1*, *AKT2*, *AKT3*, *ANGPT1*, *ANGPT2*, *ANGPT4*, *ARAF*, *AREG*, *ARRB1*, *ARRB2*, *ATF2*, *ATF4*, *BDNF*, *BRAF*, *CACNA1A*, *CACNA1B*, *CACNA1C*, *CACNA1D*, *CACNA1E*, *CACNA1F*, *CACNA1G*, *CACNA1H*, *CACNA1I*, *CACNA1S*, *CACNA2D1*, *CACNA2D2*, *CACNA2D3*, *CACNA2D4*, *CACNB1*, *CACNB2*, *CACNB3*, *CACNB4*, *CACNG1*, *CACNG2*, *CACNG3*, *CACNG4*, *CACNG5*, *CACNG6*, *CACNG7*, *CACNG8*, *CASP3*, *CD14*, *CDC25B*, *CDC42*, *CHUK*, *CRK*, *CRKL*, *CSF1*, *CSF1R*, *DAXX*, *DDIT3*, *DUSP1*, *DUSP10*, *DUSP16*, *DUSP2*, *DUSP3*, *DUSP4*, *DUSP5*, *DUSP6*, *DUSP7*, *DUSP8*, *DUSP9*, *ECSIT*, *EFNA1*, *EFNA2*, *EFNA3*, *EFNA4*, *EFNA5*, *EGF*, *EGFR*, *ELK1*, *ELK4*, *EPHA2*, *ERBB2*, *ERBB3*, *ERBB4*, *EREG*, *FAS*, *FASLG*, *FGF1*, *FGF10*, *FGF16*, *FGF17*, *FGF18*, *FGF19*, *FGF2*, *FGF20*, *FGF21*, *FGF22*, *FGF23*, *FGF3*, *FGF4*, *FGF5*, *FGF6*, *FGF7*, *FGF8*, *FGF9*, *FGFR1*, *FGFR2*, *FGFR3*, *FGFR4*, *FLNA*, *FLNB*, *FLNC*, *FLT1*, *FLT3*, *FLT3LG*, *FLT4*, *FOS*, *GADD45A*, *GADD45B*, *GADD45G*, *GNA12*, *GNG12*, *GRB2*, *HGF*, *HRAS*, *HSPA1A*, *HSPA1B*, *HSPA1L*, *HSPA2*, *HSPA6*, *HSPA8*, *HSPB1*, *IGF1*, *IGF1R*, *IGF2*, *IKBKB*, *IKBKG*, *IL1A*, *IL1B*, *IL1R1*, *IL1RAP*, *INS*, *INSR*, *IRAK1*, *IRAK4*, *JMJD7-PLA2G4B*, *JUN*, *JUND*, *KDR*, *KIT*, *KITLG*, *KRAS*, *LAMTOR3*, *MAP2K1*, *MAP2K2*, *MAP2K3*, *MAP2K4*, *MAP2K5*, *MAP2K6*, *MAP2K7*, *MAP3K1*, *MAP3K11*, *MAP3K12*, *MAP3K13*, *MAP3K14*, *MAP3K2*, *MAP3K20*, *MAP3K3*, *MAP3K4*, *MAP3K5*, *MAP3K6*, *MAP3K7*, *MAP3K8*, *MAP4K1*, *MAP4K2*, *MAP4K3*, *MAP4K4*, *MAPK1*, *MAPK10*, *MAPK11*, *MAPK12*, *MAPK13*, *MAPK14*, *MAPK3*, *MAPK7*, *MAPK8*, *MAPK8IP1*, *MAPK8IP2*, *MAPK8IP3*, *MAPK9*, *MAPKAPK2*, *MAPKAPK3*, *MAPKAPK5*, *MAPT*, *MAX*, *MECOM*, *MEF2C*, *MET*, *MKNK1*, *MKNK2*, *MRAS*, *MYC*, *MYD88*, *NF1*, *NFATC1*, *NFATC3*, *NFKB1*, *NFKB2*, *NGF*, *NGFR*, *NLK*, *NR4A1*, *NRAS*, *NTF3*, *NTF4*, *NTRK1*, *NTRK2*, *PAK1*, *PAK2*, *PDGFA*, *PDGFB*, *PDGFC*, *PDGFD*, *PDGFRA*, *PDGFRB*, *PGF*, *PLA2G4A*, *PLA2G4B*, *PLA2G4C*, *PLA2G4D*, *PLA2G4E*, *PLA2G4F*, *PPM1A*, *PPM1B*, *PPP3CA*, *PPP3CB*, *PPP3CC*, *PPP3R1*, *PPP3R2*, *PPP5C*, *PRKACA*, *PRKACB*, *PRKACG*, *PRKCA*, *PRKCB*, *PRKCG*, *PTPN5*, *PTPN7*, *PTPRR*, *RAC1*, *RAC2*, *RAC3*, *RAF1*, *RAP1A*, *RAP1B*, *RAPGEF2*, *RASA1*, *RASA2*, *RASGRF1*, *RASGRF2*, *RASGRP1*, *RASGRP2*, *RASGRP3*, *RASGRP4*, *RELA*, *RELB*, *RPS6KA1*, *RPS6KA2*, *RPS6KA3*, *RPS6KA4*, *RPS6KA5*, *RPS6KA6*, *RRAS*, *RRAS2*, *SOS1*, *SOS2*, *SRF*, *STK3*, *STK4*, *STMN1*, *TAB1*, *TAB2*, *TAOK1*, *TAOK2*, *TAOK3*, *TEK*, *TGFA*, *TGFB1*, *TGFB2*, *TGFB3*, *TGFBR1*, *TGFBR2*, *TNF*, *TNFRSF1A*, *TP53*, *TRADD*, *TRAF2*, *TRAF6*, *VEGFA*, *VEGFB*, *VEGFC*, *VEGFD*
**PI3KC signaling pathway** *BPNT2*, *CALM1*, *CALM2*, *CALM3*, *CALML3*, *CALML4*, *CALML5*, *CALML6*, *CDIPT*, *CDS1*, *CDS2*, *DGKA*, *DGKB*, *DGKD*, *DGKE*, *DGKG*, *DGKH*, *DGKI*, *DGKK*, *DGKQ*, *DGKZ*, *IMPA1*, *IMPA2*, *INPP1*, *INPP4A*, *INPP4B*, *INPP5A*, *INPP5B*, *INPP5D*, *INPP5E*, *INPP5F*, *INPPL1*, *IP6K1*, *IP6K2*, *IP6K3*, *IPMK*, *IPPK*, *ITPK1*, *ITPKA*, *ITPKB*, *ITPKC*, *ITPR1*, *ITPR2*, *ITPR3*, *MTM1*, *MTMR1*, *MTMR14*, *MTMR2*, *MTMR3*, *MTMR4*, *MTMR6*, *MTMR7*, *MTMR8*, *OCRL*, *PI4K2A*, *PI4K2B*, *PI4KA*, *PI4KB*, *PIK3C2A*, *PIK3C2B*, *PIK3C2G*, *PIK3C3*, *PIK3CA*, *PIK3CB*, *PIK3CD*, *PIK3R1*, *PIK3R2*, *PIK3R3*, *PIKFYVE*, *PIP4K2A*, *PIP4K2B*, *PIP4K2C*, *PIP4P1*, *PIP4P2*, *PIP5K1A*, *PIP5K1B*, *PIP5K1C*, *PLCB1*, *PLCB2*, *PLCB3*, *PLCB4*, *PLCD1*, *PLCD3*, *PLCD4*, *PLCE1*, *PLCG1*, *PLCG2*, *PLCZ1*, *PPIP5K1*, *PPIP5K2*, *PRKCA*, *PRKCB*, *PRKCG*, *PTEN*, *SACM1L*, *SYNJ1*, *SYNJ2*
**TGFB1 signaling pathway** *ACVR1*, *ACVR1B*, *ACVR1C*, *ACVR2A*, *ACVR2B*, *AMH*, *AMHR2*, *BAMBI*, *BMP2*, *BMP4*, *BMP5*, *BMP6*, *BMP7*, *BMP8A*, *BMP8B*, *BMPR1A*, *BMPR1B*, *BMPR2*, *CDKN2B*, *CHRD*, *CREBBP*, *CUL1*, *DCN*, *E2F4*, *E2F5*, *EP300*, *FBN1*, *FMOD*, *FST*, *GDF5*, *GDF6*, *GDF7*, *GREM1*, *GREM2*, *HAMP*, *HJV*, *ID1*, *ID2*, *ID3*, *ID4*, *IFNG*, *INHBA*, *INHBB*, *INHBC*, *INHBE*, *LEFTY1*, *LEFTY2*, *LTBP1*, *MAPK1*, *MAPK3*, *MICOS10-NBL1*, *MYC*, *NBL1*, *NEO1*, *NODAL*, *NOG*, *PITX2*, *PPP2CA*, *PPP2CB*, *PPP2R1A*, *PPP2R1B*, *RBL1*, *RBX1*, *RGMA*, *RGMB*, *RHOA*, *ROCK1*, *RPS6KB1*, *RPS6KB2*, *SKP1*, *SMAD1*, *SMAD2*, *SMAD3*, *SMAD4*, *SMAD5*, *SMAD6*, *SMAD7*, *SMAD9*, *SMURF1*, *SMURF2*, *SP1*, *TFDP1*, *TGFB1*, *TGFB2*, *TGFB3*, *TGFBR1*, *TGFBR2*, *TGIF1*, *TGIF2*, *THBS1*, *THSD4*, *TNF*, *ZFYVE16*, *ZFYVE9*
**NOTCH signaling pathway** *ADAM17*, *APH1A*, *APH1B*, *ATXN1*, *ATXN1L*, *CIR1*, *CREBBP*, *CTBP1*, *CTBP2*, *DLL1*, *DLL3*, *DLL4*, *DTX1*, *DTX2*, *DTX3*, *DTX3L*, *DTX4*, *DVL1*, *DVL2*, *DVL3*, *EP300*, *HDAC1*, *HDAC2*, *HES1*, *HES5*, *HEY1*, *HEY2*, *HEYL*, *JAG1*, *JAG2*, *KAT2A*, *KAT2B*, *LFNG*, *MAML1*, *MAML2*, *MAML3*, *MFNG*, *NCOR2*, *NCSTN*, *NOTCH1*, *NOTCH2*, *NOTCH3*, *NOTCH4*, *NUMB*, *NUMBL*, *PSEN1*, *PSEN2*, *PSENEN*, *PTCRA*, *RBPJ*, *RBPJL*, *RFNG*, *SNW1*, *TLE1*, *TLE2*, *TLE3*, *TLE4*, *TLE6*, *TLE7*
**JAK signaling pathway** *AKT1*, *AKT2*, *AKT3*, *AOX1*, *BCL2*, *BCL2L1*, *CCND1*, *CCND2*, *CCND3*, *CDKN1A*, *CISH*, *CNTF*, *CNTFR*, *CREBBP*, *CRLF2*, *CSF2*, *CSF2RA*, *CSF2RB*, *CSF3*, *CSF3R*, *CSH1*, *CSH2*, *CTF1*, *EGF*, *EGFR*, *EP300*, *EPO*, *EPOR*, *FHL1*, *GFAP*, *GH1*, *GH2*, *GHR*, *GRB2*, *HRAS*, *IFNA1*, *IFNA10*, *IFNA13*, *IFNA14*, *IFNA16*, *IFNA17*, *IFNA2*, *IFNA21*, *IFNA4*, *IFNA5*, *IFNA6*, *IFNA7*, *IFNA8*, *IFNAR1*, *IFNAR2*, *IFNB1*, *IFNE*, *IFNG*, *IFNGR1*, *IFNGR2*, *IFNK*, *IFNL1*, *IFNL2*, *IFNL3*, *IFNLR1*, *IFNW1*, *IL10*, *IL10RA*, *IL10RB*, *IL11*, *IL11RA*, *IL12A*, *IL12B*, *IL12RB1*, *IL12RB2*, *IL13*, *IL13RA1*, *IL13RA2*, *IL15*, *IL15RA*, *IL17D*, *IL19*, *IL2*, *IL20*, *IL20RA*, *IL20RB*, *IL21*, *IL21R*, *IL22*, *IL22RA1*, *IL22RA2*, *IL23A*, *IL23R*, *IL24*, *IL27RA*, *IL2RA*, *IL2RB*, *IL2RG*, *IL3*, *IL3RA*, *IL4*, *IL4R*, *IL5*, *IL5RA*, *IL6*, *IL6R*, *IL6ST*, *IL7*, *IL7R*, *IL9*, *IL9R*, *IRF9*, *JAK1*, *JAK2*, *JAK3*, *LEP*, *LEPR*, *LIF*, *LIFR*, *MCL1*, *MPL*, *MTOR*, *MYC*, *OSM*, *OSMR*, *PDGFA*, *PDGFB*, *PDGFRA*, *PDGFRB*, *PIAS1*, *PIAS2*, *PIAS3*, *PIAS4*, *PIK3CA*, *PIK3CB*, *PIK3CD*, *PIK3R1*, *PIK3R2*, *PIK3R3*, *PIM1*, *PRL*, *PRLR*, *PTPN11*, *PTPN2*, *PTPN6*, *RAF1*, *SOCS1*, *SOCS2*, *SOCS3*, *SOCS4*, *SOCS5*, *SOCS6*, *SOCS7*, *SOS1*, *SOS2*, *STAM*, *STAM2*, *STAT1*, *STAT2*, *STAT3*, *STAT4*, *STAT5A*, *STAT5B*, *STAT6*, *THPO*, *TSLP*, *TYK2*

* There are only published studies for underlined genes, so only these genes were meta-analyzed.

**Table 2:** Results of the meta-analyses of statistically significant polymorphisms listed in alphabetical order based on genotype counts.

Gene	Variant	RS	Studies (n)	Cases/Controls (n)	RE OR_G_	95% LL	95% UL	I^2^(%)	P_Q_	P_E_	Current Status
**Diseased Controls versus Cases**											
*ACE*	I > D	rs4646994	66	11437/10984	1.22	1.10	1.35	76.34	0.00	0.70	updated
	All in HWE	I > D	56	9383/8847	1.28	1.16	1.41	67.29	0.00	0.65	
*AGT*	M235T	rs699	26	5015/5253	1.21	1.01	1.45	82,45	0,00	0.84	[13]
	All in HWE		19	3181/3655	1.09	0.92	1.31	72.76	0.00	0.95	
*EPO*	G > T	rs1617640	3	1618/954	1.64	1.43	1.89	0.00	0.78	0.03	[13]
*GREM1*		rs1129456 (A/T)	2	859/940	1.55	1.23	1.94	1.02	0.31	na	new
*IL1B*	−511C > T	rs16944	3	774/667	1.66	1.38	2.01	0.00	0.86	0.28	[13]
	All in HWE		3								
*IL10*	−1082 A > G	rs1800896	4	677/761	1.23	1.01	1.49	0	0.56	0.63	[13]
		All in HWE	2	610/690	1.25	1.02	1.53	0	0.62	na	
*NOS3*	T-786C	rs2070744	9	2288/2154	1.21	1.08	1.36	0.00	0.62	0.40	updated
	All in HWE		7	2026/1862	1.21	1.08	1.37	3.08	0.40	0.29	
*NOS3*	G894T	rs1799983	21	4538/3774	1.19	0.98	1.44	77.97	<0.001	0.36	updated
		All in HWE	19	4306/3564	1.24	1.02	1.51	77.68	0.00	0.20	
*NOS3*		rs869109213	2	354/444	1.47	1.11	1.95	0.00	0.95	na	updated
		All in HWE	2							na	
**Healthy Controls versus Cases**											
*ACE*	I > D	rs4646994	30	3690/4927	1.24	1.02	1.52	83.20	0.00	0.03	[13]
	All in HWE	I > D	29	3283/4695	1.26	1.02	1.55	82.87	0.00	0.01	
*NOS3*	T-786C	rs2070744	9	1583/2142	1.42	1.13	1.77	58.00	0.01	0.84	updated
	All in HWE		8	1516/2042	1.41	1.11	1.79	63.04	0.01	0.80	
*NOS3*	G894T	rs1799983	11	2295/2737	1.64	1.21	2.22	82.07	<0.001	0.11	updated
	All in HWE		10	2247/2467	1.55	1.14	2.11	82.40	<0.001	0.21	
*NOS3*		rs869109213	2	354/444	1.52	1.12	2.06	17.59	0.27	na	updated
	All in HWE		2								
*TGFB1*	T869C	rs1800470	6	814/1450	1.30	0.86	1.96	83.64	0	0.18	
		All in HWE	4	706/1103	1.73	1.46	2.04	0	0.41	0.21	
**Healthy Controls versus Diseased Controls versus Cases**											
*IL6*	G(−174)C	rs1800795	2	90/234/212	1.44	1.10	1.89	0.00	0.42	na	updated
	All in HWE		1								
*NOS3*	T-786C	rs2070744	5	1307/1117/1451	1.29	1.17	1.43	0.00	0.53	0.46	updated
	All in HWE		4	1240/1080/1351	1.29	1.16	1.43	3.59	0.37	0.51	
*NOS3*	G894T	rs1799983	8	1506/1255/1642	1.28	1.05	1.56	70.00	0.01	0.32	updated
	All in HWE		7		1.35	1.17	1.56	40.52	0.12	0.41	
*NOS3*		rs869109213	2	354/444/515	1.30	1.04	1.63	30.45	0.23	na	updated
	All in HWE		2								

RS: SNP identifier, RE OR_G_: generalized random-effects odds ratio, LL: lower limit, UL: upper limit, I^2^: I^2^ statistic, P_Q_: *p*-value from heterogeneity testing, P_E_: *p*-value from Egger’s test.

**Table 3:** Results of the meta-analyses of statistically significant polymorphisms listed in alphabetical order based on allele counts.

GENE	Variant	RS	Studies (n)	Cases/ Controls (n)	RE OR	95% LL	95% UL	I^2^ (%)	P_Q_	P_E_	Current Status
**Diseased Controls versus Cases**											
*EDN1*		rs1794849	3	1176/1323	1.16	1.02	1.31	0	0.62	0.08	[13]
*FLT4*		rs2242221	3	1176/1323	1.14	1.01	1.29	0	0.38	0.43	[13]
*IGF2/INS/TH cluster*		rs1004446	3	1176/1323	1.16	1.03	1.31	0	0.49	0.22	[13]
*IGF2/INS/TH cluster*		rs4320932	3	1176/1323	0.84	0.73	0.96	0	0.43	0.06	[13]
*VEGFA*	C > A	rs2146323	3	1176/1323	0.85	0.76	0.95	0.2		0.2	[13]
**Healthy Controls versus Cases**											
*IL12RB1*		rs372889	2	1674/1719	1.243	1.130	1.367	0	0.567	-	new

RS: SNP identifier, RE OR_G_: random effects odds ratio generalized, LL: lower limit, UL: upper limit, I^2^: I^2^ statistic, P_Q_: *p*-value from heterogeneity testing, P_E_: *p*-value from Egger’s test.

## Supplementary Materials

The following supporting information can be downloaded at: https://www.mdpi.com/article/10.3390/ijms232315331/s1.
