# Supplementary material for: Key Genetic Components of Fibrosis in Diabetic Nephropathy: An Updated Systematic Review and Meta-Analysis
Source: Int J Mol Sci. 2022 Dec 5;23(23):15331. doi: 10.3390/ijms232315331 (PMC9736240; doi:10.3390/ijms232315331)
Supplement: Supplementary file 1 [file ijms-23-15331-s001.zip › Supplementary Table S1.docx]

Table S1: Acronyms of the genes participated in ACE signaling pathway.

| *ACE* | angiotensin I converting enzyme |
| --- | --- |
| *ACE2* | angiotensin converting enzyme 2 |
| *AGT* | angiotensinogen |
| *AGTR1* | angiotensin II receptor type 1 |
| *AGTR2* | angiotensin II receptor type 2 |
| *ANPEP* | alanyl aminopeptidase, membrane |
| *ATP6AP2* | ATPase H+ transporting accessory protein 2 |
| *CMA1* | chymase 1 |
| *CPA3* | carboxypeptidase A3 |
| *CTSA* | cathepsin A |
| *CTSG* | cathepsin G |
| *ENPEP* | glutamyl aminopeptidase |
| *KLK1* | kallikrein 1 |
| *KLK2* | kallikrein related peptidase 2 |
| *LNPEP* | leucyl and cystinyl aminopeptidase |
| *MAS1* | MAS1 proto-oncogene, G protein-coupled receptor |
| *MME* | membrane metalloendopeptidase |
| *MRGPRD* | MAS related GPR family member D |
| *NLN* | neurolysin |
| *PRCP* | prolylcarboxypeptidase |
| *PREP* | prolyl endopeptidase |
| *REN* | renin |
| *THOP1* | thimet oligopeptidase 1 |
