# Supplementary material for: Key Genetic Components of Fibrosis in Diabetic Nephropathy: An Updated Systematic Review and Meta-Analysis
Source: Int J Mol Sci. 2022 Dec 5;23(23):15331. doi: 10.3390/ijms232315331 (PMC9736240; doi:10.3390/ijms232315331)
Supplement: Supplementary file 1 [file ijms-23-15331-s001.zip › Supplementary Table S2.docx]

**Table S2:** Acronyms of the genes participated in relaxin signaling pathway.

| *ACTA2* | actin alpha 2, smooth muscle |
| --- | --- |
| *ADCY1* | adenylate cyclase 1 |
| *ADCY2* | adenylate cyclase 2 |
| *ADCY3* | adenylate cyclase 3 |
| *ADCY4* | adenylate cyclase 4 |
| *ADCY5* | adenylate cyclase 5 |
| *ADCY6* | adenylate cyclase 6 |
| *ADCY7* | adenylate cyclase 7 |
| *ADCY8* | adenylate cyclase 8 |
| *ADCY9* | adenylate cyclase 9 |
| *AKT1* | AKT serine/threonine kinase 1 |
| *AKT2* | AKT serine/threonine kinase 2 |
| *AKT3* | AKT serine/threonine kinase 3 |
| *ARRB1* | arrestin beta 1 |
| *ARRB2* | arrestin beta 2 |
| *ATF2* | activating transcription factor 2 |
| *ATF4* | activating transcription factor 4 |
| *ATF6B* | activating transcription factor 6 beta |
| *COL1A1* | collagen type I alpha 1 chain |
| *COL1A2* | collagen type I alpha 2 chain |
| *COL3A1* | collagen type III alpha 1 chain |
| *COL4A1* | collagen type IV alpha 1 chain |
| *COL4A2* | collagen type IV alpha 2 chain |
| *COL4A3* | collagen type IV alpha 3 chain |
| *COL4A4* | collagen type IV alpha 4 chain |
| *COL4A5* | collagen type IV alpha 5 chain |
| *COL4A6* | collagen type IV alpha 6 chain |
| *CREB1* | cAMP responsive element binding protein 1 |
| *CREB3* | cAMP responsive element binding protein 3 |
| *CREB3L1* | cAMP responsive element binding protein 3 like 1 |
| *CREB3L2* | cAMP responsive element binding protein 3 like 2 |
| *CREB3L3* | cAMP responsive element binding protein 3 like 3 |
| *CREB3L4* | cAMP responsive element binding protein 3 like 4 |
| *CREB5* | cAMP responsive element binding protein 5 |
| *EDN1* | endothelin 1 |
| *EDNRB* | endothelin receptor type B |
| *EGFR* | epidermal growth factor receptor |
| *FOS* | Fos proto-oncogene, AP-1 transcription factor subunit |
| *GNA15* | G protein subunit alpha 15 |
| *GNAI1* | G protein subunit alpha i1 |
| *GNAI2* | G protein subunit alpha i2 |
| *GNAI3* | G protein subunit alpha i3 |
| *GNAO1* | G protein subunit alpha o1 |
| *GNAS* | GNAS complex locus |
| *GNB1* | G protein subunit beta 1 |
| *GNB2* | G protein subunit beta 2 |
| *GNB3* | G protein subunit beta 3 |
| *GNB4* | G protein subunit beta 4 |
| *GNB5* | G protein subunit beta 5 |
| *GNG10* | G protein subunit gamma 10 |
| *GNG11* | G protein subunit gamma 11 |
| *GNG12* | G protein subunit gamma 12 |
| *GNG13* | G protein subunit gamma 13 |
| *GNG2* | G protein subunit gamma 2 |
| *GNG3* | G protein subunit gamma 3 |
| *GNG4* | G protein subunit gamma 4 |
| *GNG5* | G protein subunit gamma 5 |
| *GNG7* | G protein subunit gamma 7 |
| *GNG8* | G protein subunit gamma 8 |
| *GNGT1* | G protein subunit gamma transducin 1 |
| *GNGT2* | G protein subunit gamma transducin 2 |
| *GRB2* | growth factor receptor bound protein 2 |
| *HRAS* | HRas proto-oncogene, GTPase |
| *INSL3* | insulin like 3 |
| *INSL5* | insulin like 5 |
| *JUN* | Jun proto-oncogene, AP-1 transcription factor subunit |
| *KRAS* | KRAS proto-oncogene, GTPase |
| *MAP2K1* | mitogen-activated protein kinase kinase 1 |
| *MAP2K2* | mitogen-activated protein kinase kinase 2 |
| *MAP2K4* | mitogen-activated protein kinase kinase 4 |
| *MAP2K7* | mitogen-activated protein kinase kinase 7 |
| *MAPK1* | mitogen-activated protein kinase 1 |
| *MAPK10* | mitogen-activated protein kinase 10 |
| *MAPK11* | mitogen-activated protein kinase 11 |
| *MAPK12* | mitogen-activated protein kinase 12 |
| *MAPK13* | mitogen-activated protein kinase 13 |
| *MAPK14* | mitogen-activated protein kinase 14 |
| *MAPK3* | mitogen-activated protein kinase 3 |
| *MAPK8* | mitogen-activated protein kinase 8 |
| *MAPK9* | mitogen-activated protein kinase 9 |
| *MMP1* | matrix metallopeptidase 1 |
| *MMP13* | matrix metallopeptidase 13 |
| *MMP2* | matrix metallopeptidase 2 |
| *MMP9* | matrix metallopeptidase 9 |
| *NFKB1* | nuclear factor kappa B subunit 1 |
| *NFKBIA* | NFKB inhibitor alpha |
| *NOS1* | nitric oxide synthase 1 |
| *NOS2* | nitric oxide synthase 2 |
| *NOS3* | nitric oxide synthase 3 |
| *NRAS* | NRAS proto-oncogene, GTPase |
| *PIK3CA* | phosphatidylinositol-4,5-bisphosphate 3-kinase catalytic subunit alpha |
| *PIK3CB* | phosphatidylinositol-4,5-bisphosphate 3-kinase catalytic subunit beta |
| *PIK3CD* | phosphatidylinositol-4,5-bisphosphate 3-kinase catalytic subunit delta |
| *PIK3R1* | phosphoinositide-3-kinase regulatory subunit 1 |
| *PIK3R2* | phosphoinositide-3-kinase regulatory subunit 2 |
| *PIK3R3* | phosphoinositide-3-kinase regulatory subunit 3 |
| *PLCB1* | phospholipase C beta 1 |
| *PLCB2* | phospholipase C beta 2 |
| *PLCB3* | phospholipase C beta 3 |
| *PLCB4* | phospholipase C beta 4 |
| *PRKACA* | protein kinase cAMP-activated catalytic subunit alpha |
| *PRKACB* | protein kinase cAMP-activated catalytic subunit beta |
| *PRKACG* | protein kinase cAMP-activated catalytic subunit gamma |
| *PRKCA* | protein kinase C alpha |
| *PRKCZ* | protein kinase C zeta |
| *RAF1* | Raf-1 proto-oncogene, serine/threonine kinase |
| *RELA* | RELA proto-oncogene, NF-kB subunit |
| *RLN1* | relaxin 1 |
| *RLN2* | relaxin 2 |
| *RLN3* | relaxin 3 |
| *RXFP1* | relaxin family peptide receptor 1 |
| *RXFP2* | relaxin family peptide receptor 2 |
| *RXFP3* | relaxin family peptide receptor 3 |
| *RXFP4* | relaxin family peptide/INSL5 receptor 4 |
| *SHC1* | SHC adaptor protein 1 |
| *SHC2* | SHC adaptor protein 2 |
| *SHC3* | SHC adaptor protein 3 |
| *SHC4* | SHC adaptor protein 4 |
| *SMAD2* | SMAD family member 2 |
| *SOS1* | SOS Ras/Rac guanine nucleotide exchange factor 1 |
| *SOS2* | SOS Ras/Rho guanine nucleotide exchange factor 2 |
| *SRC* | SRC proto-oncogene, non-receptor tyrosine kinase |
| *TGFB1* | transforming growth factor beta 1 |
| *TGFBR1* | transforming growth factor beta receptor 1 |
| *TGFBR2* | transforming growth factor beta receptor 2 |
| *VEGFA* | vascular endothelial growth factor A |
| *VEGFB* | vascular endothelial growth factor B |
| *VEGFC* | vascular endothelial growth factor C |
| *VEGFD* | vascular endothelial growth factor D |
