# Supplementary material for: Key Genetic Components of Fibrosis in Diabetic Nephropathy: An Updated Systematic Review and Meta-Analysis
Source: Int J Mol Sci. 2022 Dec 5;23(23):15331. doi: 10.3390/ijms232315331 (PMC9736240; doi:10.3390/ijms232315331)
Supplement: Supplementary file 1 [file ijms-23-15331-s001.zip › Supplementary Table S3.docx]

**Table S3:** Acronyms of the genes participated in WNT signaling pathway.

| *APC* | APC regulator of WNT signaling pathway |
| --- | --- |
| *APC2* | APC regulator of WNT signaling pathway 2 |
| *APCDD1* | APC down-regulated 1 |
| *APCDD1L* | APC down-regulated 1 like |
| *AXIN1* | axin 1 |
| *AXIN2* | axin 2 |
| *BAMBI* | BMP and activin membrane bound inhibitor |
| *BTRC* | beta-transducin repeat containing E3 ubiquitin protein ligase |
| *CACYBP* | calcyclin binding protein |
| *CAMK2A* | calcium/calmodulin dependent protein kinase II alpha |
| *CAMK2B* | calcium/calmodulin dependent protein kinase II beta |
| *CAMK2D* | calcium/calmodulin dependent protein kinase II delta |
| *CAMK2G* | calcium/calmodulin dependent protein kinase II gamma |
| *CBY1* | chibby family member 1, beta catenin antagonist |
| *CCDC88C* | coiled-coil domain containing 88C |
| *CCN4* | cellular communication network factor 4 |
| *CCND1* | cyclin D1 |
| *CCND2* | cyclin D2 |
| *CCND3* | cyclin D3 |
| *CER1* | cerberus 1, DAN family BMP antagonist |
| *CHD8* | chromodomain helicase DNA binding protein 8 |
| *CREBBP* | CREB binding protein |
| *CSNK1A1* | casein kinase 1 alpha 1 |
| *CSNK1A1L* | casein kinase 1 alpha 1 like |
| *CSNK1E* | casein kinase 1 epsilon |
| *CSNK2A1* | casein kinase 2 alpha 1 |
| *CSNK2A2* | casein kinase 2 alpha 2 |
| *CSNK2A3* | casein kinase 2 alpha 3 |
| *CSNK2B* | casein kinase 2 beta |
| *CTBP1* | C-terminal binding protein 1 |
| *CTBP2* | C-terminal binding protein 2 |
| *CTNNB1* | catenin beta 1 |
| *CTNNBIP1* | catenin beta interacting protein 1 |
| *CTNND2* | catenin delta 2 |
| *CUL1* | cullin 1 |
| *CXXC4* | CXXC finger protein 4 |
| *DAAM1* | dishevelled associated activator of morphogenesis 1 |
| *DAAM2* | dishevelled associated activator of morphogenesis 2 |
| *DKK1* | dickkopf WNT signaling pathway inhibitor 1 |
| *DKK2* | dickkopf WNT signaling pathway inhibitor 2 |
| *DKK4* | dickkopf WNT signaling pathway inhibitor 4 |
| *DVL1* | dishevelled segment polarity protein 1 |
| *DVL2* | dishevelled segment polarity protein 2 |
| *DVL3* | dishevelled segment polarity protein 3 |
| *EP300* | E1A binding protein p300 |
| *FBXW11* | F-box and WD repeat domain containing 11 |
| *FOSL1* | FOS like 1, AP-1 transcription factor subunit |
| *FRAT1* | FRAT regulator of WNT signaling pathway 1 |
| *FRAT2* | FRAT regulator of WNT signaling pathway 2 |
| *FRZB* | frizzled related protein |
| *FZD1* | frizzled class receptor 1 |
| *FZD10* | frizzled class receptor 10 |
| *FZD2* | frizzled class receptor 2 |
| *FZD3* | frizzled class receptor 3 |
| *FZD4* | frizzled class receptor 4 |
| *FZD5* | frizzled class receptor 5 |
| *FZD6* | frizzled class receptor 6 |
| *FZD7* | frizzled class receptor 7 |
| *FZD8* | frizzled class receptor 8 |
| *FZD9* | frizzled class receptor 9 |
| *GPC4* | glypican 4 |
| *GSK3B* | glycogen synthase kinase 3 beta |
| *INVS* | inversin |
| *JUN* | Jun proto-oncogene, AP-1 transcription factor subunit |
| *LEF1* | lymphoid enhancer binding factor 1 |
| *LGR4* | leucine rich repeat containing G protein-coupled receptor 4 |
| *LGR5* | leucine rich repeat containing G protein-coupled receptor 5 |
| *LGR6* | leucine rich repeat containing G protein-coupled receptor 6 |
| *LRP5* | LDL receptor related protein 5 |
| *LRP6* | LDL receptor related protein 6 |
| *MAP3K7* | mitogen-activated protein kinase kinase kinase 7 |
| *MAPK10* | mitogen-activated protein kinase 10 |
| *MAPK8* | mitogen-activated protein kinase 8 |
| *MAPK9* | mitogen-activated protein kinase 9 |
| *MMP7* | matrix metallopeptidase 7 |
| *MYC* | MYC proto-oncogene, bHLH transcription factor |
| *NFATC1* | nuclear factor of activated T cells 1 |
| *NFATC2* | nuclear factor of activated T cells 2 |
| *NFATC3* | nuclear factor of activated T cells 3 |
| *NFATC4* | nuclear factor of activated T cells 4 |
| *NKD1* | NKD inhibitor of WNT signaling pathway 1 |
| *NKD2* | NKD inhibitor of WNT signaling pathway 2 |
| *NLK* | nemo like kinase |
| *NOTUM* | notum, palmitoleoyl-protein carboxylesterase |
| *PLCB1* | phospholipase C beta 1 |
| *PLCB2* | phospholipase C beta 2 |
| *PLCB3* | phospholipase C beta 3 |
| *PLCB4* | phospholipase C beta 4 |
| *PORCN* | porcupine O-acyltransferase |
| *PPARD* | peroxisome proliferator activated receptor delta |
| *PPP3CA* | protein phosphatase 3 catalytic subunit alpha |
| *PPP3CB* | protein phosphatase 3 catalytic subunit beta |
| *PPP3CC* | protein phosphatase 3 catalytic subunit gamma |
| *PPP3R1* | protein phosphatase 3 regulatory subunit B, alpha |
| *PPP3R2* | protein phosphatase 3 regulatory subunit B, beta |
| *PRICKLE1* | prickle planar cell polarity protein 1 |
| *PRICKLE2* | prickle planar cell polarity protein 2 |
| *PRICKLE3* | prickle planar cell polarity protein 3 |
| *PRICKLE4* | prickle planar cell polarity protein 4 |
| *PRKACA* | protein kinase cAMP-activated catalytic subunit alpha |
| *PRKACB* | protein kinase cAMP-activated catalytic subunit beta |
| *PRKACG* | protein kinase cAMP-activated catalytic subunit gamma |
| *PRKCA* | protein kinase C alpha |
| *PRKCB* | protein kinase C beta |
| *PRKCG* | protein kinase C gamma |
| *PSEN1* | presenilin 1 |
| *RAC1* | Rac family small GTPase 1 |
| *RAC2* | Rac family small GTPase 2 |
| *RAC3* | Rac family small GTPase 3 |
| *RBX1* | ring-box 1 |
| *RHOA* | ras homolog family member A |
| *RNF43* | ring finger protein 43 |
| *ROCK2* | Rho associated coiled-coil containing protein kinase 2 |
| *ROR1* | receptor tyrosine kinase like orphan receptor 1 |
| *ROR2* | receptor tyrosine kinase like orphan receptor 2 |
| *RSPO1* | R-spondin 1 |
| *RSPO2* | R-spondin 2 |
| *RSPO3* | R-spondin 3 |
| *RSPO4* | R-spondin 4 |
| *RUVBL1* | RuvB like AAA ATPase 1 |
| *RYK* | receptor like tyrosine kinase |
| *SENP2* | SUMO specific peptidase 2 |
| *SERPINF1* | serpin family F member 1 |
| *SFRP1* | secreted frizzled related protein 1 |
| *SFRP2* | secreted frizzled related protein 2 |
| *SFRP4* | secreted frizzled related protein 4 |
| *SFRP5* | secreted frizzled related protein 5 |
| *SIAH1* | siah E3 ubiquitin protein ligase 1 |
| *SKP1* | S-phase kinase associated protein 1 |
| *SMAD3* | SMAD family member 3 |
| *SMAD4* | SMAD family member 4 |
| *SOST* | sclerostin |
| *SOX17* | SRY-box transcription factor 17 |
| *TBL1X* | transducin beta like 1 X-linked |
| *TBL1XR1* | TBL1X/Y related 1 |
| *TBL1Y* | transducin beta like 1 Y-linked |
| *TCF7* | transcription factor 7 |
| *TCF7L1* | transcription factor 7 like 1 |
| *TCF7L2* | transcription factor 7 like 2 |
| *TLE1* | TLE family member 1, transcriptional corepressor |
| *TLE2* | TLE family member 2, transcriptional corepressor |
| *TLE3* | TLE family member 3, transcriptional corepressor |
| *TLE4* | TLE family member 4, transcriptional corepressor |
| *TLE6* | TLE family member 6, subcortical maternal complex member |
| *TLE7* | TLE family member 7 |
| *TP53* | tumor protein p53 |
| *TPTEP2-CSNK1E* | TPTEP2-CSNK1E readthrough |
| *VANGL1* | VANGL planar cell polarity protein 1 |
| *VANGL2* | VANGL planar cell polarity protein 2 |
| *WIF1* | WNT inhibitory factor 1 |
| *WNT1* | Wnt family member 1 |
| *WNT10A* | Wnt family member 10A |
| *WNT10B* | Wnt family member 10B |
| *WNT11* | Wnt family member 11 |
| *WNT16* | Wnt family member 16 |
| *WNT2* | Wnt family member 2 |
| *WNT2B* | Wnt family member 2B |
| *WNT3* | Wnt family member 3 |
| *WNT3A* | Wnt family member 3A |
| *WNT4* | Wnt family member 4 |
| *WNT5A* | Wnt family member 5A |
| *WNT5B* | Wnt family member 5B |
| *WNT6* | Wnt family member 6 |
| *WNT7A* | Wnt family member 7A |
| *WNT7B* | Wnt family member 7B |
| *WNT8A* | Wnt family member 8A |
| *WNT8B* | Wnt family member 8B |
| *WNT9A* | Wnt family member 9A |
| *WNT9B* | Wnt family member 9B |
| *ZNRF3* | zinc and ring finger 3 |
