# Supplementary material for: Key Genetic Components of Fibrosis in Diabetic Nephropathy: An Updated Systematic Review and Meta-Analysis
Source: Int J Mol Sci. 2022 Dec 5;23(23):15331. doi: 10.3390/ijms232315331 (PMC9736240; doi:10.3390/ijms232315331)
Supplement: Supplementary file 1 [file ijms-23-15331-s001.zip › Supplementary Table S4.docx]

**Table S4:** Acronyms of the genes participated in MAPK signaling pathway.

| *AKT1* | AKT serine/threonine kinase 1 |
| --- | --- |
| *AKT2* | AKT serine/threonine kinase 2 |
| *AKT3* | AKT serine/threonine kinase 3 |
| *ANGPT1* | angiopoietin 1 |
| *ANGPT2* | angiopoietin 2 |
| *ANGPT4* | angiopoietin 4 |
| *ARAF* | A-Raf proto-oncogene, serine/threonine kinase |
| *AREG* | amphiregulin |
| *ARRB1* | arrestin beta 1 |
| *ARRB2* | arrestin beta 2 |
| *ATF2* | activating transcription factor 2 |
| *ATF4* | activating transcription factor 4 |
| *BDNF* | brain derived neurotrophic factor |
| *BRAF* | B-Raf proto-oncogene, serine/threonine kinase |
| *CACNA1A* | calcium voltage-gated channel subunit alpha1 A |
| *CACNA1B* | calcium voltage-gated channel subunit alpha1 B |
| *CACNA1C* | calcium voltage-gated channel subunit alpha1 C |
| *CACNA1D* | calcium voltage-gated channel subunit alpha1 D |
| *CACNA1E* | calcium voltage-gated channel subunit alpha1 E |
| *CACNA1F* | calcium voltage-gated channel subunit alpha1 F |
| *CACNA1G* | calcium voltage-gated channel subunit alpha1 G |
| *CACNA1H* | calcium voltage-gated channel subunit alpha1 H |
| *CACNA1I* | calcium voltage-gated channel subunit alpha1 I |
| *CACNA1S* | calcium voltage-gated channel subunit alpha1 S |
| *CACNA2D1* | calcium voltage-gated channel auxiliary subunit alpha2delta 1 |
| *CACNA2D2* | calcium voltage-gated channel auxiliary subunit alpha2delta 2 |
| *CACNA2D3* | calcium voltage-gated channel auxiliary subunit alpha2delta 3 |
| *CACNA2D4* | calcium voltage-gated channel auxiliary subunit alpha2delta 4 |
| *CACNB1* | calcium voltage-gated channel auxiliary subunit beta 1 |
| *CACNB2* | calcium voltage-gated channel auxiliary subunit beta 2 |
| *CACNB3* | calcium voltage-gated channel auxiliary subunit beta 3 |
| *CACNB4* | calcium voltage-gated channel auxiliary subunit beta 4 |
| *CACNG1* | calcium voltage-gated channel auxiliary subunit gamma 1 |
| *CACNG2* | calcium voltage-gated channel auxiliary subunit gamma 2 |
| *CACNG3* | calcium voltage-gated channel auxiliary subunit gamma 3 |
| *CACNG4* | calcium voltage-gated channel auxiliary subunit gamma 4 |
| *CACNG5* | calcium voltage-gated channel auxiliary subunit gamma 5 |
| *CACNG6* | calcium voltage-gated channel auxiliary subunit gamma 6 |
| *CACNG7* | calcium voltage-gated channel auxiliary subunit gamma 7 |
| *CACNG8* | calcium voltage-gated channel auxiliary subunit gamma 8 |
| *CASP3* | caspase 3 |
| *CD14* | CD14 molecule |
| *CDC25B* | cell division cycle 25B |
| *CDC42* | cell division cycle 42 |
| *CHUK* | component of inhibitor of nuclear factor kappa B kinase complex |
| *CRK* | CRK proto-oncogene, adaptor protein |
| *CRKL* | CRK like proto-oncogene, adaptor protein |
| *CSF1* | colony stimulating factor 1 |
| *CSF1R* | colony stimulating factor 1 receptor |
| *DAXX* | death domain associated protein |
| *DDIT3* | DNA damage inducible transcript 3 |
| *DUSP1* | dual specificity phosphatase 1 |
| *DUSP10* | dual specificity phosphatase 10 |
| *DUSP16* | dual specificity phosphatase 16 |
| *DUSP2* | dual specificity phosphatase 2 |
| *DUSP3* | dual specificity phosphatase 3 |
| *DUSP4* | dual specificity phosphatase 4 |
| *DUSP5* | dual specificity phosphatase 5 |
| *DUSP6* | dual specificity phosphatase 6 |
| *DUSP7* | dual specificity phosphatase 7 |
| *DUSP8* | dual specificity phosphatase 8 |
| *DUSP9* | dual specificity phosphatase 9 |
| *ECSIT* | ECSIT signaling integrator |
| *EFNA1* | ephrin A1 |
| *EFNA2* | ephrin A2 |
| *EFNA3* | ephrin A3 |
| *EFNA4* | ephrin A4 |
| *EFNA5* | ephrin A5 |
| *EGF* | epidermal growth factor |
| *EGFR* | epidermal growth factor receptor |
| *ELK1* | ETS transcription factor ELK1 |
| *ELK4* | ETS transcription factor ELK4 |
| *EPHA2* | EPH receptor A2 |
| *ERBB2* | erb-b2 receptor tyrosine kinase 2 |
| *ERBB3* | erb-b2 receptor tyrosine kinase 3 |
| *ERBB4* | erb-b2 receptor tyrosine kinase 4 |
| *EREG* | epiregulin |
| *FAS* | Fas cell surface death receptor |
| *FASLG* | Fas ligand |
| *FGF1* | fibroblast growth factor 1 |
| *FGF10* | fibroblast growth factor 10 |
| *FGF16* | fibroblast growth factor 16 |
| *FGF17* | fibroblast growth factor 17 |
| *FGF18* | fibroblast growth factor 18 |
| *FGF19* | fibroblast growth factor 19 |
| *FGF2* | fibroblast growth factor 2 |
| *FGF20* | fibroblast growth factor 20 |
| *FGF21* | fibroblast growth factor 21 |
| *FGF22* | fibroblast growth factor 22 |
| *FGF23* | fibroblast growth factor 23 |
| *FGF3* | fibroblast growth factor 3 |
| *FGF4* | fibroblast growth factor 4 |
| *FGF5* | fibroblast growth factor 5 |
| *FGF6* | fibroblast growth factor 6 |
| *FGF7* | fibroblast growth factor 7 |
| *FGF8* | fibroblast growth factor 8 |
| *FGF9* | fibroblast growth factor 9 |
| *FGFR1* | fibroblast growth factor receptor 1 |
| *FGFR2* | fibroblast growth factor receptor 2 |
| *FGFR3* | fibroblast growth factor receptor 3 |
| *FGFR4* | fibroblast growth factor receptor 4 |
| *FLNA* | filamin A |
| *FLNB* | filamin B |
| *FLNC* | filamin C |
| *FLT1* | fms related receptor tyrosine kinase 1 |
| *FLT3* | fms related receptor tyrosine kinase 3 |
| *FLT3LG* | fms related receptor tyrosine kinase 3 ligand |
| *FLT4* | fms related receptor tyrosine kinase 4 |
| *FOS* | Fos proto-oncogene, AP-1 transcription factor subunit |
| *GADD45A* | growth arrest and DNA damage inducible alpha |
| *GADD45B* | growth arrest and DNA damage inducible beta |
| *GADD45G* | growth arrest and DNA damage inducible gamma |
| *GNA12* | G protein subunit alpha 12 |
| *GNG12* | G protein subunit gamma 12 |
| *GRB2* | growth factor receptor bound protein 2 |
| *HGF* | hepatocyte growth factor |
| *HRAS* | HRas proto-oncogene, GTPase |
| *HSPA1A* | heat shock protein family A (Hsp70) member 1A |
| *HSPA1B* | heat shock protein family A (Hsp70) member 1B |
| *HSPA1L* | heat shock protein family A (Hsp70) member 1 like |
| *HSPA2* | heat shock protein family A (Hsp70) member 2 |
| *HSPA6* | heat shock protein family A (Hsp70) member 6 |
| *HSPA8* | heat shock protein family A (Hsp70) member 8 |
| *HSPB1* | heat shock protein family B (small) member 1 |
| *IGF1* | insulin like growth factor 1 |
| *IGF1R* | insulin like growth factor 1 receptor |
| *IGF2* | insulin like growth factor 2 |
| *IKBKB* | inhibitor of nuclear factor kappa B kinase subunit beta |
| *IKBKG* | inhibitor of nuclear factor kappa B kinase regulatory subunit gamma |
| *IL1A* | interleukin 1 alpha |
| *IL1B* | interleukin 1 beta |
| *IL1R1* | interleukin 1 receptor type 1 |
| *IL1RAP* | interleukin 1 receptor accessory protein |
| *INS* | insulin |
| *INSR* | insulin receptor |
| *IRAK1* | interleukin 1 receptor associated kinase 1 |
| *IRAK4* | interleukin 1 receptor associated kinase 4 |
| *JMJD7-PLA2G4B* | JMJD7-PLA2G4B readthrough |
| *JUN* | Jun proto-oncogene, AP-1 transcription factor subunit |
| *JUND* | JunD proto-oncogene, AP-1 transcription factor subunit |
| *KDR* | kinase insert domain receptor |
| *KIT* | KIT proto-oncogene, receptor tyrosine kinase |
| *KITLG* | KIT ligand |
| *KRAS* | KRAS proto-oncogene, GTPase |
| *LAMTOR3* | late endosomal/lysosomal adaptor, MAPK and MTOR activator 3 |
| *MAP2K1* | mitogen-activated protein kinase kinase 1 |
| *MAP2K2* | mitogen-activated protein kinase kinase 2 |
| *MAP2K3* | mitogen-activated protein kinase kinase 3 |
| *MAP2K4* | mitogen-activated protein kinase kinase 4 |
| *MAP2K5* | mitogen-activated protein kinase kinase 5 |
| *MAP2K6* | mitogen-activated protein kinase kinase 6 |
| *MAP2K7* | mitogen-activated protein kinase kinase 7 |
| *MAP3K1* | mitogen-activated protein kinase kinase kinase 1 |
| *MAP3K11* | mitogen-activated protein kinase kinase kinase 11 |
| *MAP3K12* | mitogen-activated protein kinase kinase kinase 12 |
| *MAP3K13* | mitogen-activated protein kinase kinase kinase 13 |
| *MAP3K14* | mitogen-activated protein kinase kinase kinase 14 |
| *MAP3K2* | mitogen-activated protein kinase kinase kinase 2 |
| *MAP3K20* | mitogen-activated protein kinase kinase kinase 20 |
| *MAP3K3* | mitogen-activated protein kinase kinase kinase 3 |
| *MAP3K4* | mitogen-activated protein kinase kinase kinase 4 |
| *MAP3K5* | mitogen-activated protein kinase kinase kinase 5 |
| *MAP3K6* | mitogen-activated protein kinase kinase kinase 6 |
| *MAP3K7* | mitogen-activated protein kinase kinase kinase 7 |
| *MAP3K8* | mitogen-activated protein kinase kinase kinase 8 |
| *MAP4K1* | mitogen-activated protein kinase kinase kinase kinase 1 |
| *MAP4K2* | mitogen-activated protein kinase kinase kinase kinase 2 |
| *MAP4K3* | mitogen-activated protein kinase kinase kinase kinase 3 |
| *MAP4K4* | mitogen-activated protein kinase kinase kinase kinase 4 |
| *MAPK1* | mitogen-activated protein kinase 1 |
| *MAPK10* | mitogen-activated protein kinase 10 |
| *MAPK11* | mitogen-activated protein kinase 11 |
| *MAPK12* | mitogen-activated protein kinase 12 |
| *MAPK13* | mitogen-activated protein kinase 13 |
| *MAPK14* | mitogen-activated protein kinase 14 |
| *MAPK3* | mitogen-activated protein kinase 3 |
| *MAPK7* | mitogen-activated protein kinase 7 |
| *MAPK8* | mitogen-activated protein kinase 8 |
| *MAPK8IP1* | mitogen-activated protein kinase 8 interacting protein 1 |
| *MAPK8IP2* | mitogen-activated protein kinase 8 interacting protein 2 |
| *MAPK8IP3* | mitogen-activated protein kinase 8 interacting protein 3 |
| *MAPK9* | mitogen-activated protein kinase 9 |
| *MAPKAPK2* | MAPK activated protein kinase 2 |
| *MAPKAPK3* | MAPK activated protein kinase 3 |
| *MAPKAPK5* | MAPK activated protein kinase 5 |
| *MAPT* | microtubule associated protein tau |
| *MAX* | MYC associated factor X |
| *MECOM* | MDS1 and EVI1 complex locus |
| *MEF2C* | myocyte enhancer factor 2C |
| *MET* | MET proto-oncogene, receptor tyrosine kinase |
| *MKNK1* | MAPK interacting serine/threonine kinase 1 |
| *MKNK2* | MAPK interacting serine/threonine kinase 2 |
| *MRAS* | muscle RAS oncogene homolog |
| *MYC* | MYC proto-oncogene, bHLH transcription factor |
| *MYD88* | MYD88 innate immune signal transduction adaptor |
| *NF1* | neurofibromin 1 |
| *NFATC1* | nuclear factor of activated T cells 1 |
| *NFATC3* | nuclear factor of activated T cells 3 |
| *NFKB1* | nuclear factor kappa B subunit 1 |
| *NFKB2* | nuclear factor kappa B subunit 2 |
| *NGF* | nerve growth factor |
| *NGFR* | nerve growth factor receptor |
| *NLK* | nemo like kinase |
| *NR4A1* | nuclear receptor subfamily 4 group A member 1 |
| *NRAS* | NRAS proto-oncogene, GTPase |
| *NTF3* | neurotrophin 3 |
| *NTF4* | neurotrophin 4 |
| *NTRK1* | neurotrophic receptor tyrosine kinase 1 |
| *NTRK2* | neurotrophic receptor tyrosine kinase 2 |
| *PAK1* | p21 (RAC1) activated kinase 1 |
| *PAK2* | p21 (RAC1) activated kinase 2 |
| *PDGFA* | platelet derived growth factor subunit A |
| *PDGFB* | platelet derived growth factor subunit B |
| *PDGFC* | platelet derived growth factor C |
| *PDGFD* | platelet derived growth factor D |
| *PDGFRA* | platelet derived growth factor receptor alpha |
| *PDGFRB* | platelet derived growth factor receptor beta |
| *PGF* | placental growth factor |
| *PLA2G4A* | phospholipase A2 group IVA |
| *PLA2G4B* | phospholipase A2 group IVB |
| *PLA2G4C* | phospholipase A2 group IVC |
| *PLA2G4D* | phospholipase A2 group IVD |
| *PLA2G4E* | phospholipase A2 group IVE |
| *PLA2G4F* | phospholipase A2 group IVF |
| *PPM1A* | protein phosphatase, Mg2+/Mn2+ dependent 1A |
| *PPM1B* | protein phosphatase, Mg2+/Mn2+ dependent 1B |
| *PPP3CA* | protein phosphatase 3 catalytic subunit alpha |
| *PPP3CB* | protein phosphatase 3 catalytic subunit beta |
| *PPP3CC* | protein phosphatase 3 catalytic subunit gamma |
| *PPP3R1* | protein phosphatase 3 regulatory subunit B, alpha |
| *PPP3R2* | protein phosphatase 3 regulatory subunit B, beta |
| *PPP5C* | protein phosphatase 5 catalytic subunit |
| *PRKACA* | protein kinase cAMP-activated catalytic subunit alpha |
| *PRKACB* | protein kinase cAMP-activated catalytic subunit beta |
| *PRKACG* | protein kinase cAMP-activated catalytic subunit gamma |
| *PRKCA* | protein kinase C alpha |
| *PRKCB* | protein kinase C beta |
| *PRKCG* | protein kinase C gamma |
| *PTPN5* | protein tyrosine phosphatase non-receptor type 5 |
| *PTPN7* | protein tyrosine phosphatase non-receptor type 7 |
| *PTPRR* | protein tyrosine phosphatase receptor type R |
| *RAC1* | Rac family small GTPase 1 |
| *RAC2* | Rac family small GTPase 2 |
| *RAC3* | Rac family small GTPase 3 |
| *RAF1* | Raf-1 proto-oncogene, serine/threonine kinase |
| *RAP1A* | RAP1A, member of RAS oncogene family |
| *RAP1B* | RAP1B, member of RAS oncogene family |
| *RAPGEF2* | Rap guanine nucleotide exchange factor 2 |
| *RASA1* | RAS p21 protein activator 1 |
| *RASA2* | RAS p21 protein activator 2 |
| *RASGRF1* | Ras protein specific guanine nucleotide releasing factor 1 |
| *RASGRF2* | Ras protein specific guanine nucleotide releasing factor 2 |
| *RASGRP1* | RAS guanyl releasing protein 1 |
| *RASGRP2* | RAS guanyl releasing protein 2 |
| *RASGRP3* | RAS guanyl releasing protein 3 |
| *RASGRP4* | RAS guanyl releasing protein 4 |
| *RELA* | RELA proto-oncogene, NF-kB subunit |
| *RELB* | RELB proto-oncogene, NF-kB subunit |
| *RPS6KA1* | ribosomal protein S6 kinase A1 |
| *RPS6KA2* | ribosomal protein S6 kinase A2 |
| *RPS6KA3* | ribosomal protein S6 kinase A3 |
| *RPS6KA4* | ribosomal protein S6 kinase A4 |
| *RPS6KA5* | ribosomal protein S6 kinase A5 |
| *RPS6KA6* | ribosomal protein S6 kinase A6 |
| *RRAS* | RAS related |
| *RRAS2* | RAS related 2 |
| *SOS1* | SOS Ras/Rac guanine nucleotide exchange factor 1 |
| *SOS2* | SOS Ras/Rho guanine nucleotide exchange factor 2 |
| *SRF* | serum response factor |
| *STK3* | serine/threonine kinase 3 |
| *STK4* | serine/threonine kinase 4 |
| *STMN1* | stathmin 1 |
| *TAB1* | TGF-beta activated kinase 1 (MAP3K7) binding protein 1 |
| *TAB2* | TGF-beta activated kinase 1 (MAP3K7) binding protein 2 |
| *TAOK1* | TAO kinase 1 |
| *TAOK2* | TAO kinase 2 |
| *TAOK3* | TAO kinase 3 |
| *TEK* | TEK receptor tyrosine kinase |
| *TGFA* | transforming growth factor alpha |
| *TGFB1* | transforming growth factor beta 1 |
| *TGFB2* | transforming growth factor beta 2 |
| *TGFB3* | transforming growth factor beta 3 |
| *TGFBR1* | transforming growth factor beta receptor 1 |
| *TGFBR2* | transforming growth factor beta receptor 2 |
| *TNF* | tumor necrosis factor |
| *TNFRSF1A* | TNF receptor superfamily member 1A |
| *TP53* | tumor protein p53 |
| *TRADD* | TNFRSF1A associated via death domain |
| *TRAF2* | TNF receptor associated factor 2 |
| *TRAF6* | TNF receptor associated factor 6 |
| *VEGFA* | vascular endothelial growth factor A |
| *VEGFB* | vascular endothelial growth factor B |
| *VEGFC* | vascular endothelial growth factor C |
| *VEGFD* | vascular endothelial growth factor D |
