# Supplementary material for: Key Genetic Components of Fibrosis in Diabetic Nephropathy: An Updated Systematic Review and Meta-Analysis
Source: Int J Mol Sci. 2022 Dec 5;23(23):15331. doi: 10.3390/ijms232315331 (PMC9736240; doi:10.3390/ijms232315331)
Supplement: Supplementary file 1 [file ijms-23-15331-s001.zip › Supplementary Table S5.docx]

**Table S5:** Acronyms of the genes included in PI3KC signaling pathway.

| *BPNT2* | 3'(2'), 5'-bisphosphate nucleotidase 2 |
| --- | --- |
| *CALM1* | calmodulin 1 |
| *CALM2* | calmodulin 2 |
| *CALM3* | calmodulin 3 |
| *CALML3* | calmodulin like 3 |
| *CALML4* | calmodulin like 4 |
| *CALML5* | calmodulin like 5 |
| *CALML6* | calmodulin like 6 |
| *CDIPT* | CDP-diacylglycerol--inositol 3-phosphatidyltransferase |
| *CDS1* | CDP-diacylglycerol synthase 1 |
| *CDS2* | CDP-diacylglycerol synthase 2 |
| *DGKA* | diacylglycerol kinase alpha |
| *DGKB* | diacylglycerol kinase beta |
| *DGKD* | diacylglycerol kinase delta |
| *DGKE* | diacylglycerol kinase epsilon |
| *DGKG* | diacylglycerol kinase gamma |
| *DGKH* | diacylglycerol kinase eta |
| *DGKI* | diacylglycerol kinase iota |
| *DGKK* | diacylglycerol kinase kappa |
| *DGKQ* | diacylglycerol kinase theta |
| *DGKZ* | diacylglycerol kinase zeta |
| *IMPA1* | inositol monophosphatase 1 |
| *IMPA2* | inositol monophosphatase 2 |
| *INPP1* | inositol polyphosphate-1-phosphatase |
| *INPP4A* | inositol polyphosphate-4-phosphatase type I A |
| *INPP4B* | inositol polyphosphate-4-phosphatase type II B |
| *INPP5A* | inositol polyphosphate-5-phosphatase A |
| *INPP5B* | inositol polyphosphate-5-phosphatase B |
| *INPP5D* | inositol polyphosphate-5-phosphatase D |
| *INPP5E* | inositol polyphosphate-5-phosphatase E |
| *INPP5F* | inositol polyphosphate-5-phosphatase F |
| *INPPL1* | inositol polyphosphate phosphatase like 1 |
| *IP6K1* | inositol hexakisphosphate kinase 1 |
| *IP6K2* | inositol hexakisphosphate kinase 2 |
| *IP6K3* | inositol hexakisphosphate kinase 3 |
| *IPMK* | inositol polyphosphate multikinase |
| *IPPK* | inositol-pentakisphosphate 2-kinase |
| *ITPK1* | inositol-tetrakisphosphate 1-kinase |
| *ITPKA* | inositol-trisphosphate 3-kinase A |
| *ITPKB* | inositol-trisphosphate 3-kinase B |
| *ITPKC* | inositol-trisphosphate 3-kinase C |
| *ITPR1* | inositol 1,4,5-trisphosphate receptor type 1 |
| *ITPR2* | inositol 1,4,5-trisphosphate receptor type 2 |
| *ITPR3* | inositol 1,4,5-trisphosphate receptor type 3 |
| *MTM1* | myotubularin 1 |
| *MTMR1* | myotubularin related protein 1 |
| *MTMR14* | myotubularin related protein 14 |
| *MTMR2* | myotubularin related protein 2 |
| *MTMR3* | myotubularin related protein 3 |
| *MTMR4* | myotubularin related protein 4 |
| *MTMR6* | myotubularin related protein 6 |
| *MTMR7* | myotubularin related protein 7 |
| *MTMR8* | myotubularin related protein 8 |
| *OCRL* | OCRL inositol polyphosphate-5-phosphatase |
| *PI4K2A* | phosphatidylinositol 4-kinase type 2 alpha |
| *PI4K2B* | phosphatidylinositol 4-kinase type 2 beta |
| *PI4KA* | phosphatidylinositol 4-kinase alpha |
| *PI4KB* | phosphatidylinositol 4-kinase beta |
| *PIK3C2A* | phosphatidylinositol-4-phosphate 3-kinase catalytic subunit type 2 alpha |
| *PIK3C2B* | phosphatidylinositol-4-phosphate 3-kinase catalytic subunit type 2 beta |
| *PIK3C2G* | phosphatidylinositol-4-phosphate 3-kinase catalytic subunit type 2 gamma |
| *PIK3C3* | phosphatidylinositol 3-kinase catalytic subunit type 3 |
| *PIK3CA* | phosphatidylinositol-4,5-bisphosphate 3-kinase catalytic subunit alpha |
| *PIK3CB* | phosphatidylinositol-4,5-bisphosphate 3-kinase catalytic subunit beta |
| *PIK3CD* | phosphatidylinositol-4,5-bisphosphate 3-kinase catalytic subunit delta |
| *PIK3R1* | phosphoinositide-3-kinase regulatory subunit 1 |
| *PIK3R2* | phosphoinositide-3-kinase regulatory subunit 2 |
| *PIK3R3* | phosphoinositide-3-kinase regulatory subunit 3 |
| *PIKFYVE* | phosphoinositide kinase, FYVE-type zinc finger containing |
| *PIP4K2A* | phosphatidylinositol-5-phosphate 4-kinase type 2 alpha |
| *PIP4K2B* | phosphatidylinositol-5-phosphate 4-kinase type 2 beta |
| *PIP4K2C* | phosphatidylinositol-5-phosphate 4-kinase type 2 gamma |
| *PIP4P1* | phosphatidylinositol-4,5-bisphosphate 4-phosphatase 1 |
| *PIP4P2* | phosphatidylinositol-4,5-bisphosphate 4-phosphatase 2 |
| *PIP5K1A* | phosphatidylinositol-4-phosphate 5-kinase type 1 alpha |
| *PIP5K1B* | phosphatidylinositol-4-phosphate 5-kinase type 1 beta |
| *PIP5K1C* | phosphatidylinositol-4-phosphate 5-kinase type 1 gamma |
| *PLCB1* | phospholipase C beta 1 |
| *PLCB2* | phospholipase C beta 2 |
| *PLCB3* | phospholipase C beta 3 |
| *PLCB4* | phospholipase C beta 4 |
| *PLCD1* | phospholipase C delta 1 |
| *PLCD3* | phospholipase C delta 3 |
| *PLCD4* | phospholipase C delta 4 |
| *PLCE1* | phospholipase C epsilon 1 |
| *PLCG1* | phospholipase C gamma 1 |
| *PLCG2* | phospholipase C gamma 2 |
| *PLCZ1* | phospholipase C zeta 1 |
| *PPIP5K1* | diphosphoinositol pentakisphosphate kinase 1 |
| *PPIP5K2* | diphosphoinositol pentakisphosphate kinase 2 |
| *PRKCA* | protein kinase C alpha |
| *PRKCB* | protein kinase C beta |
| *PRKCG* | protein kinase C gamma |
| *PTEN* | phosphatase and tensin homolog |
| *SACM1L* | SAC1 like phosphatidylinositide phosphatase |
| *SYNJ1* | synaptojanin 1 |
| *SYNJ2* | synaptojanin 2 |
