# Supplementary material for: Key Genetic Components of Fibrosis in Diabetic Nephropathy: An Updated Systematic Review and Meta-Analysis
Source: Int J Mol Sci. 2022 Dec 5;23(23):15331. doi: 10.3390/ijms232315331 (PMC9736240; doi:10.3390/ijms232315331)
Supplement: Supplementary file 1 [file ijms-23-15331-s001.zip › Supplementary Table S6.docx]

**Table S6:** Acronyms of the genes participated in TGFB signaling pathway.

| *ACVR1* | activin A receptor type 1 |
| --- | --- |
| *ACVR1B* | activin A receptor type 1B |
| *ACVR1C* | activin A receptor type 1C |
| *ACVR2A* | activin A receptor type 2A |
| *ACVR2B* | activin A receptor type 2B |
| *AMH* | anti-Mullerian hormone |
| *AMHR2* | anti-Mullerian hormone receptor type 2 |
| *BAMBI* | BMP and activin membrane bound inhibitor |
| *BMP2* | bone morphogenetic protein 2 |
| *BMP4* | bone morphogenetic protein 4 |
| *BMP5* | bone morphogenetic protein 5 |
| *BMP6* | bone morphogenetic protein 6 |
| *BMP7* | bone morphogenetic protein 7 |
| *BMP8A* | bone morphogenetic protein 8a |
| *BMP8B* | bone morphogenetic protein 8b |
| *BMPR1A* | bone morphogenetic protein receptor type 1A |
| *BMPR1B* | bone morphogenetic protein receptor type 1B |
| *BMPR2* | bone morphogenetic protein receptor type 2 |
| *CDKN2B* | cyclin dependent kinase inhibitor 2B |
| *CHRD* | chordin |
| *CREBBP* | CREB binding protein |
| *CUL1* | cullin 1 |
| *DCN* | decorin |
| *E2F4* | E2F transcription factor 4 |
| *E2F5* | E2F transcription factor 5 |
| *EP300* | E1A binding protein p300 |
| *FBN1* | fibrillin 1 |
| *FMOD* | fibromodulin |
| *FST* | follistatin |
| *GDF5* | growth differentiation factor 5 |
| *GDF6* | growth differentiation factor 6 |
| *GDF7* | growth differentiation factor 7 |
| *GREM1* | gremlin 1, DAN family BMP antagonist |
| *GREM2* | gremlin 2, DAN family BMP antagonist |
| *HAMP* | hepcidin antimicrobial peptide |
| *HJV* | hemojuvelin BMP co-receptor |
| *ID1* | inhibitor of DNA binding 1, HLH protein |
| *ID2* | inhibitor of DNA binding 2 |
| *ID3* | inhibitor of DNA binding 3, HLH protein |
| *ID4* | inhibitor of DNA binding 4, HLH protein |
| *IFNG* | interferon gamma |
| *INHBA* | inhibin subunit beta A |
| *INHBB* | inhibin subunit beta B |
| *INHBC* | inhibin subunit beta C |
| *INHBE* | inhibin subunit beta E |
| *LEFTY1* | left-right determination factor 1 |
| *LEFTY2* | left-right determination factor 2 |
| *LTBP1* | latent transforming growth factor beta binding protein 1 |
| *MAPK1* | mitogen-activated protein kinase 1 |
| *MAPK3* | mitogen-activated protein kinase 3 |
| *MICOS10-NBL1* | MICOS10-NBL1 readthrough |
| *MYC* | MYC proto-oncogene, bHLH transcription factor |
| *NBL1* | NBL1, DAN family BMP antagonist |
| *NEO1* | neogenin 1 |
| *NODAL* | nodal growth differentiation factor |
| *NOG* | noggin |
| *PITX2* | paired like homeodomain 2 |
| *PPP2CA* | protein phosphatase 2 catalytic subunit alpha |
| *PPP2CB* | protein phosphatase 2 catalytic subunit beta |
| *PPP2R1A* | protein phosphatase 2 scaffold subunit Aalpha |
| *PPP2R1B* | protein phosphatase 2 scaffold subunit Abeta |
| *RBL1* | RB transcriptional corepressor like 1 |
| *RBX1* | ring-box 1 |
| *RGMA* | repulsive guidance molecule BMP co-receptor a |
| *RGMB* | repulsive guidance molecule BMP co-receptor b |
| *RHOA* | ras homolog family member A |
| *ROCK1* | Rho associated coiled-coil containing protein kinase 1 |
| *RPS6KB1* | ribosomal protein S6 kinase B1 |
| *RPS6KB2* | ribosomal protein S6 kinase B2 |
| *SKP1* | S-phase kinase associated protein 1 |
| *SMAD1* | SMAD family member 1 |
| *SMAD2* | SMAD family member 2 |
| *SMAD3* | SMAD family member 3 |
| *SMAD4* | SMAD family member 4 |
| *SMAD5* | SMAD family member 5 |
| *SMAD6* | SMAD family member 6 |
| *SMAD7* | SMAD family member 7 |
| *SMAD9* | SMAD family member 9 |
| *SMURF1* | SMAD specific E3 ubiquitin protein ligase 1 |
| *SMURF2* | SMAD specific E3 ubiquitin protein ligase 2 |
| *SP1* | Sp1 transcription factor |
| *TFDP1* | transcription factor Dp-1 |
| *TGFB1* | transforming growth factor beta 1 |
| *TGFB2* | transforming growth factor beta 2 |
| *TGFB3* | transforming growth factor beta 3 |
| *TGFBR1* | transforming growth factor beta receptor 1 |
| *TGFBR2* | transforming growth factor beta receptor 2 |
| *TGIF1* | TGFB induced factor homeobox 1 |
| *TGIF2* | TGFB induced factor homeobox 2 |
| *THBS1* | thrombospondin 1 |
| *THSD4* | thrombospondin type 1 domain containing 4 |
| *TNF* | tumor necrosis factor |
| *ZFYVE16* | zinc finger FYVE-type containing 16 |
| *ZFYVE9* | zinc finger FYVE-type containing 9 |
