# Supplementary material for: Key Genetic Components of Fibrosis in Diabetic Nephropathy: An Updated Systematic Review and Meta-Analysis
Source: Int J Mol Sci. 2022 Dec 5;23(23):15331. doi: 10.3390/ijms232315331 (PMC9736240; doi:10.3390/ijms232315331)
Supplement: Supplementary file 1 [file ijms-23-15331-s001.zip › Supplementary Table S7 NOTCH.docx]

**Table S7:** Acronyms of the gens participated in NOTCH signaling pathway.

| ADAM17 | ADAM metallopeptidase domain 17 |
| --- | --- |
| APH1A | aph-1 homolog A, gamma-secretase subunit |
| APH1B | aph-1 homolog B, gamma-secretase subunit |
| ATXN1 | ataxin 1 |
| ATXN1L | ataxin 1 like |
| CIR1 | corepressor interacting with RBPJ, CIR1 |
| CREBBP | CREB binding protein |
| CTBP1 | C-terminal binding protein 1 |
| CTBP2 | C-terminal binding protein 2 |
| DLL1 | delta like canonical Notch ligand 1 |
| DLL3 | delta like canonical Notch ligand 3 |
| DLL4 | delta like canonical Notch ligand 4 |
| DTX1 | deltex E3 ubiquitin ligase 1 |
| DTX2 | deltex E3 ubiquitin ligase 2 |
| DTX3 | deltex E3 ubiquitin ligase 3 |
| DTX3L | deltex E3 ubiquitin ligase 3L |
| DTX4 | deltex E3 ubiquitin ligase 4 |
| DVL1 | dishevelled segment polarity protein 1 |
| DVL2 | dishevelled segment polarity protein 2 |
| DVL3 | dishevelled segment polarity protein 3 |
| EP300 | E1A binding protein p300 |
| HDAC1 | histone deacetylase 1 |
| HDAC2 | histone deacetylase 2 |
| HES1 | hes family bHLH transcription factor 1 |
| HES5 | hes family bHLH transcription factor 5 |
| HEY1 | hes related family bHLH transcription factor with YRPW motif 1 |
| HEY2 | hes related family bHLH transcription factor with YRPW motif 2 |
| HEYL | hes related family bHLH transcription factor with YRPW motif like |
| JAG1 | jagged canonical Notch ligand 1 |
| JAG2 | jagged canonical Notch ligand 2 |
| KAT2A | lysine acetyltransferase 2A |
| KAT2B | lysine acetyltransferase 2B |
| LFNG | LFNG O-fucosylpeptide 3-beta-N-acetylglucosaminyltransferase |
| MAML1 | mastermind like transcriptional coactivator 1 |
| MAML2 | mastermind like transcriptional coactivator 2 |
| MAML3 | mastermind like transcriptional coactivator 3 |
| MFNG | MFNG O-fucosylpeptide 3-beta-N-acetylglucosaminyltransferase |
| NCOR2 | nuclear receptor corepressor 2 |
| NCSTN | nicastrin |
| NOTCH1 | notch receptor 1 |
| NOTCH2 | notch receptor 2 |
| NOTCH3 | notch receptor 3 |
| NOTCH4 | notch receptor 4 |
| NUMB | NUMB endocytic adaptor protein |
| NUMBL | NUMB like endocytic adaptor protein |
| PSEN1 | presenilin 1 |
| PSEN2 | presenilin 2 |
| PSENEN | presenilin enhancer, gamma-secretase subunit |
| PTCRA | pre T cell antigen receptor alpha |
| RBPJ | recombination signal binding protein for immunoglobulin kappa J region |
| RBPJL | recombination signal binding protein for immunoglobulin kappa J region like |
| RFNG | RFNG O-fucosylpeptide 3-beta-N-acetylglucosaminyltransferase |
| SNW1 | SNW domain containing 1 |
| TLE1 | TLE family member 1, transcriptional corepressor |
| TLE2 | TLE family member 2, transcriptional corepressor |
| TLE3 | TLE family member 3, transcriptional corepressor |
| TLE4 | TLE family member 4, transcriptional corepressor |
| TLE6 | TLE family member 6, subcortical maternal complex member |
| TLE7 | TLE family member 7 |
