# Supplementary material for: Key Genetic Components of Fibrosis in Diabetic Nephropathy: An Updated Systematic Review and Meta-Analysis
Source: Int J Mol Sci. 2022 Dec 5;23(23):15331. doi: 10.3390/ijms232315331 (PMC9736240; doi:10.3390/ijms232315331)
Supplement: Supplementary file 1 [file ijms-23-15331-s001.zip › Supplementary Table S8 JAK.docx]

**Table S8:** Acronyms of the genes participated in JAK signaling pathway.

| AKT1 | AKT serine/threonine kinase 1 |
| --- | --- |
| AKT2 | AKT serine/threonine kinase 2 |
| AKT3 | AKT serine/threonine kinase 3 |
| AOX1 | aldehyde oxidase 1 |
| BCL2 | BCL2 apoptosis regulator |
| BCL2L1 | BCL2 like 1 |
| CCND1 | cyclin D1 |
| CCND2 | cyclin D2 |
| CCND3 | cyclin D3 |
| CDKN1A | cyclin dependent kinase inhibitor 1A |
| CISH | cytokine inducible SH2 containing protein |
| CNTF | ciliary neurotrophic factor |
| CNTFR | ciliary neurotrophic factor receptor |
| CREBBP | CREB binding protein |
| CRLF2 | cytokine receptor like factor 2 |
| CSF2 | colony stimulating factor 2 |
| CSF2RA | colony stimulating factor 2 receptor subunit alpha |
| CSF2RB | colony stimulating factor 2 receptor subunit beta |
| CSF3 | colony stimulating factor 3 |
| CSF3R | colony stimulating factor 3 receptor |
| CSH1 | chorionic somatomammotropin hormone 1 |
| CSH2 | chorionic somatomammotropin hormone 2 |
| CTF1 | cardiotrophin 1 |
| EGF | epidermal growth factor |
| EGFR | epidermal growth factor receptor [KO:K04361] |
| EP300 | E1A binding protein p300 |
| EPO | erythropoietin |
| EPOR | erythropoietin receptor |
| FHL1 | four and a half LIM domains 1 |
| GFAP | glial fibrillary acidic protein |
| GH1 | growth hormone 1 |
| GH2 | growth hormone 2 |
| GHR | growth hormone receptor |
| GRB2 | growth factor receptor bound protein 2 |
| HRAS | HRas proto-oncogene, GTPase |
| IFNA1 | interferon alpha 1 |
| IFNA10 | interferon alpha 10 |
| IFNA13 | interferon alpha 13 |
| IFNA14 | interferon alpha 14 |
| IFNA16 | interferon alpha 16 |
| IFNA17 | interferon alpha 17 |
| IFNA2 | interferon alpha 2 |
| IFNA21 | interferon alpha 21 |
| IFNA4 | interferon alpha 4 |
| IFNA5 | interferon alpha 5 |
| IFNA6 | interferon alpha 6 |
| IFNA7 | interferon alpha 7 |
| IFNA8 | interferon alpha 8 |
| IFNAR1 | interferon alpha and beta receptor subunit 1 |
| IFNAR2 | interferon alpha and beta receptor subunit 2 |
| IFNB1 | interferon beta 1 |
| IFNE | interferon epsilon |
| IFNG | interferon gamma |
| IFNGR1 | interferon gamma receptor 1 |
| IFNGR2 | interferon gamma receptor 2 |
| IFNK | interferon kappa |
| IFNL1 | interferon lambda 1 |
| IFNL2 | interferon lambda 2 |
| IFNL3 | interferon lambda 3 |
| IFNLR1 | interferon lambda receptor 1 |
| IFNW1 | interferon omega 1 |
| IL10 | interleukin 10 |
| IL10RA | interleukin 10 receptor subunit alpha |
| IL10RB | interleukin 10 receptor subunit beta |
| IL11 | interleukin 11 |
| IL11RA | interleukin 11 receptor subunit alpha |
| IL12A | interleukin 12A |
| IL12B | interleukin 12B |
| IL12RB1 | interleukin 12 receptor subunit beta 1 |
| IL12RB2 | interleukin 12 receptor subunit beta 2 |
| IL13 | interleukin 13 |
| IL13RA1 | interleukin 13 receptor subunit alpha 1 |
| IL13RA2 | interleukin 13 receptor subunit alpha 2 |
| IL15 | interleukin 15 |
| IL15RA | interleukin 15 receptor subunit alpha |
| IL17D | interleukin 17D |
| IL19 | interleukin 19 |
| IL2 | interleukin 2 |
| IL20 | interleukin 20 |
| IL20RA | interleukin 20 receptor subunit alpha |
| IL20RB | interleukin 20 receptor subunit beta |
| IL21 | interleukin 21 |
| IL21R | interleukin 21 receptor |
| IL22 | interleukin 22 |
| IL22RA1 | interleukin 22 receptor subunit alpha 1 |
| IL22RA2 | interleukin 22 receptor subunit alpha 2 |
| IL23A | interleukin 23 subunit alpha |
| IL23R | interleukin 23 receptor |
| IL24 | interleukin 24 |
| IL27RA | interleukin 27 receptor subunit alpha |
| IL2RA | interleukin 2 receptor subunit alpha |
| IL2RB | interleukin 2 receptor subunit beta |
| IL2RG | interleukin 2 receptor subunit gamma |
| IL3 | interleukin 3 |
| IL3RA | interleukin 3 receptor subunit alpha |
| IL4 | interleukin 4 |
| IL4R | interleukin 4 receptor |
| IL5 | interleukin 5 |
| IL5RA | interleukin 5 receptor subunit alpha |
| IL6 | interleukin 6 |
| IL6R | interleukin 6 receptor |
| IL6ST | interleukin 6 cytokine family signal transducer |
| IL7 | interleukin 7 |
| IL7R | interleukin 7 receptor |
| IL9 | interleukin 9 |
| IL9R | interleukin 9 receptor |
| IRF9 | interferon regulatory factor 9 |
| JAK1 | Janus kinase 1 |
| JAK2 | Janus kinase 2 |
| JAK3 | Janus kinase 3 |
| LEP | leptin |
| LEPR | leptin receptor |
| LIF | LIF interleukin 6 family cytokine |
| LIFR | LIF receptor subunit alpha |
| MCL1 | MCL1 apoptosis regulator, BCL2 family member |
| MPL | MPL proto-oncogene, thrombopoietin receptor |
| MTOR | mechanistic target of rapamycin kinase |
| MYC | MYC proto-oncogene, bHLH transcription factor |
| OSM | oncostatin M |
| OSMR | oncostatin M receptor |
| PDGFA | platelet derived growth factor subunit A |
| PDGFB | platelet derived growth factor subunit B |
| PDGFRA | platelet derived growth factor receptor alpha |
| PDGFRB | platelet derived growth factor receptor beta |
| PIAS1 | protein inhibitor of activated STAT 1 |
| PIAS2 | protein inhibitor of activated STAT 2 |
| PIAS3 | protein inhibitor of activated STAT 3 |
| PIAS4 | protein inhibitor of activated STAT 4 |
| PIK3CA | phosphatidylinositol-4,5-bisphosphate 3-kinase catalytic subunit alpha |
| PIK3CB | phosphatidylinositol-4,5-bisphosphate 3-kinase catalytic subunit beta |
| PIK3CD | phosphatidylinositol-4,5-bisphosphate 3-kinase catalytic subunit delta |
| PIK3R1 | phosphoinositide-3-kinase regulatory subunit 1 |
| PIK3R2 | phosphoinositide-3-kinase regulatory subunit 2 |
| PIK3R3 | phosphoinositide-3-kinase regulatory subunit 3 |
| PIM1 | Pim-1 proto-oncogene, serine/threonine kinase |
| PRL | prolactin |
| PRLR | prolactin receptor |
| PTPN11 | protein tyrosine phosphatase non-receptor type 11 |
| PTPN2 | protein tyrosine phosphatase non-receptor type 2 |
| PTPN6 | protein tyrosine phosphatase non-receptor type 6 |
| RAF1 | Raf-1 proto-oncogene, serine/threonine kinase |
| SOCS1 | suppressor of cytokine signaling 1 |
| SOCS2 | suppressor of cytokine signaling 2 |
| SOCS3 | suppressor of cytokine signaling 3 |
| SOCS4 | suppressor of cytokine signaling 4 |
| SOCS5 | suppressor of cytokine signaling 5 |
| SOCS6 | suppressor of cytokine signaling 6 |
| SOCS7 | suppressor of cytokine signaling 7 |
| SOS1 | SOS Ras/Rac guanine nucleotide exchange factor 1 |
| SOS2 | SOS Ras/Rho guanine nucleotide exchange factor 2 |
| STAM | signal transducing adaptor molecule |
| STAM2 | signal transducing adaptor molecule 2 |
| STAT1 | signal transducer and activator of transcription 1 |
| STAT2 | signal transducer and activator of transcription 2 |
| STAT3 | signal transducer and activator of transcription 3 |
| STAT4 | signal transducer and activator of transcription 4 |
| STAT5A | signal transducer and activator of transcription 5A |
| STAT5B | signal transducer and activator of transcription 5B |
| STAT6 | signal transducer and activator of transcription 6 |
| THPO | thrombopoietin |
| TSLP | thymic stromal lymphopoietin |
| TYK2 | tyrosine kinase 2 |
