# Supplementary material for: Key Genetic Components of Fibrosis in Diabetic Nephropathy: An Updated Systematic Review and Meta-Analysis
Source: Int J Mol Sci. 2022 Dec 5;23(23):15331. doi: 10.3390/ijms232315331 (PMC9736240; doi:10.3390/ijms232315331)
Supplement: Supplementary file 1 [file ijms-23-15331-s001.zip › Supplementary Table S9 demo 2.docx]

**Supplementary Table S9**: Demographic characteristics of included studies.

| Variant | References | Ethnicity | DM | Trait | Ν | Selection criteria | Ν | Selection criteria | N | Selection criteria | HWE HT | HWE DC | Analyses |
| --- | --- | --- | --- | --- | --- | --- | --- | --- | --- | --- | --- | --- | --- |
| ACE I>D (rs4646994) | Wyawahare 2017 (1) | Asian Indians | T2DM | DN | 129 | DM≥5 yrs, pers. micr/ria | 50 | DM≥5 yrs, pers. norm/ria |  |  |  |  | DC-C |
|  | Mansouri 2017 (2) | Africans | T2DM | DN | 130 | pers. micr/ria | 85 | DM≥15 yrs, pers. norm/ria |  |  |  |  | DC-C |
|  | Fawwaz 2017 (3) | Lebanese | T2DM | DN | 50 | ESRD | 64 | DM≥10 yrs, pers. norm/ria |  |  |  |  | DC-C |
|  | Wang 2016 (4) | Asians | T2DM | DN | 301 | Pers. micr/macroalbuminuria | 497 | pers. norm/ria |  |  |  |  | DC-C |
|  | Parchwani (2015) (5) | Asians | T2DM | DN | 143 | pers. micro/macroalbuminuria | 166 | pers. norm/ria |  | - |  | No | DC-C |
|  | Fathi (2015) (6) | Asians | T2DM | DN | 255 | pers. micr/ria | 235 | pers. norm/ria |  | - |  |  | DC-C |
|  | Parchwani (2014) (7) | Asians |  | DN | 138 | pers. micro/macroalbuminuria | 142 | pers. norm/ria |  | - |  |  | DC-C |
|  | Ilic (2014) (8) | Caucasians | T1DM | DN | 46 | pers. micro/macroalbuminuria | 33 | pers. norm/ria |  | - |  |  | DC-C |
|  | Kumar (2013) (9) | Asians | T2DM | DN | 407 | pers. proteinuria> 500 mg/day | 185 | diabetics without nephropathy matched for age and gender | 232 | Healthy non-diabetics | No | No | DC-C, HT-DC-C, HT-C |
|  | El-Baz (2012) (10) | Africans | T2DM | DN | 102 | micro/macroalbuminuria, DM≥10 yrs | 100 | norm/ria , DM≥10 yrs |  | - |  | No | DC-C |
|  | Rahimi (2012) (11) | Asians | T2DM | DN | 140 | pers. micro/macroalbuminuria | 72 | pers. norm/ria |  | - |  |  | DC-C |
|  | Al-Harbi (2011) (12) | Asians | T2DM | DN | 110 | DN (from pers. proteinuria>0.1 g/day up to ESRD) | 250 | diabetics without nephropathy | 360 | Healthy non-diabetics matched for age |  |  | DC-C, HT-DC-C, HT-C |
|  | Felehgari (2011) (13) | Asians | T2DM | DN | 68 | pers. macr/ria | 72 | pers. norm/ria |  | - |  |  | DC-C |
|  | Rahimi (2011) (14) | Asians | T2DM | DN | 72 | pers. micr/ria | 72 | pers. norm/ria matched for age and gender |  | - |  |  | DC-C |
|  | Zsom (2011) (15) | Caucasians | T1DM | d. ESRD | 21 | d. ESRD |  | - | 200 | non-diabetics matched for age |  |  | HT-C |
|  | Zsom (2011) (15) | Caucasians | T2DM | d. ESRD | 93 | d. ESRD |  | - |  | non-diabetics matched for age |  |  | HT-C |
|  | Blech (2011) (16) | Asians | mostly T2DM, T1DM | DN | 556 | microalbubinuria, proteinuria or dialysis due to DM,, DM>10 yrs | 873 | norm/ria , DM>10 yrs |  |  |  |  | Alleles DC-C |
|  | Jayapalan (2010) (17) | Asians | T2DM | DN | 127 | macr/ria or diabetic ESRF | 81 | norm/ria | 137 |  |  |  | DC-C, HT-DC-C, HT-C |
|  | Currie (2010) (18) | Caucasians | T1DM | DN | 718 | pers. proteinuria>0.5 g/24h, hypertension with/without meds, retinopathy | 749 | DM≥15 yrs, norm/ria, no anti-HT meds |  | - |  | No | DC-C |
|  | Palomo-Piñón (2009) (19) | Mixed | T2DM | albuminuria | 235 | micro/macroalbuminuria | 200 | norm/ria |  | - |  |  | DC-C |
|  | Ahluwalia (2009) (20) | Asians | T2DM | DN | 240 | pers. macr/ria or CRF without microscopic hematuria | 200 | pers. norm/ria, no anti-HT meds matched for age and ethnnicity |  | - |  |  | DC-C |
|  | Ezzidi (2009) (21) | Africans | T2DM | DN | 515 | diabetics with kidney damage | 402 | pers. norm/ria | 748 |  |  |  | DC-C, HT-DC-C, HT-C |
|  | Nikzamir (2009) (22) | Asians | T2DM | DN | 177 | pers. micro/macroalbuminuria | 145 | pers. norm/ria matched for gender, age, DM duration |  | - |  |  | DC-C |
|  | Naresh (2009) (23) | Asians | T2DM | DN | 30 | proteinuria>500 mg, DR |  | - | 30 | non-diabetics |  |  | HT-C |
|  | Arfa (2008) (24) | Africans | T2DM | DN | 90 | DM≥10 yrs, pers. micro/macroalbuminuria | 51 | DM≥10 yrs and norm/ria | 103 |  |  |  | DC-C, HT-DC-C, HT-C |
|  | Mollsten (2008) (25) | Caucasians | T1DM | DN | 121 | pers. micro/macroalbuminuria | 197 | DM≥20 yrs, norm/ria , no anti-HT meds |  | - |  | No | DC-C |
|  | Movva (2007) (26) | Asians | T2DM | DN | 174 | s. Cr.>1.5 or s. albumin>30 mg/dl, DN (Stage I to ESRD), ESRD: 77% | 175 | DM≥10 yrs and norm/ria | 111 | non-diabetics matched for age and gender |  |  | DC-C, HT-DC-C, HT-C |
|  | Hadjadj (2007) (27) | Caucasians | T1DM | DN | 380 | pers. albuminuria with or without renal failure | 382 | pers. norm/ria and no ACE inhibitors or angiotensin receptor blockers |  |  |  |  | Alleles DC-C |
|  | Hadjadj (2007) (27) | Caucasians | T1DM | DN | 385 | pers. albuminuria with or without renal failure | 468 | pers. norm/ria and no ACEi or ARBs |  |  |  |  | Alleles DC-C |
|  | Hadjadj (2007) (27) | Caucasians | T1DM | DN | 277 | pers. albuminuria with or without renal failure | 273 | pers. norm/ria and no ACEi or ARBs |  |  |  |  | Alleles DC-C |
|  | Buraczynska (2006) (28) | Caucasians |  | ESRD | 141 | ESRD on dialysis due to DM |  | - | 520 | non-diabetics |  |  | Alleles HT-C |
|  | Lee (2006) (29) | E. Asians | T2DM | DN | 127 | micro/macroalbuminuria | 244 | norm/ria | 178 | non-diabetics |  |  | Alleles DC-C/ HT-C |
|  | Ng (2006) (30) | Caucasians | T2DM | DN | 291 | pers. proteinuria or ESRD | 167 | DM>6yrs and pers. norm/ria |  | - |  |  | DC-C |
|  | Prasad (2006) (31) | Asians | T2DM | diabetic CRI | 196 | DM≥2 years, moderate CRI (s. Cr. ≥3 mg/dl, macr/ria , DR) | 225 | pers. norm/ria , DM≥10 yrs |  | - |  |  | DC-C |
|  | Shestakova (2006) (32) | Caucasians | T1DM | DN | 63 | pers. macr/ria, with/without anti-HT meds | 66 | pers. norm/ria | 96 | non-diabetics |  |  | DC-C, HT-DC-C, HT-C |
|  | Park (2005) (33) | E. Asians | T2DM | diabetic ESRF | 103 | ESRF, dialysis | 88 | DM>15 yrs, normal renal function and norm/ria matched for age,  gender, BMI, HbA1c level, or lipid profiles |  | - |  | No | DC-C |
|  | Canani (2005) (34) | Latin Americans | T2DM | DN | 373 | Incipient or overt nephropathy and DR | 609 | pers. norm/ria |  | - |  |  | DC-C |
|  | Degirmenci (2005) (35) | Asians (Turkey) | T2DM | DN | 65 | pers. micr/ria no ACEi and/or ARBs | 75 | pers. norm/ria without ACEi and/or ARBs | 133 |  |  |  | DC-C, HT-DC-C, HT-C |
|  | Shin Shin (2004) (36) | E. Asians | T2DM | DN | 118 | overt nephropathy or pers. micr/ria, DM>5 yrs | 59 | norm/ria , DM>5 yrs | 129 | non-diabetics |  | No | DC-C, HT-DC-C, HT-C |
|  | Arzu Ergen (2004) (37) | Asians (Turkey) | T2DM | DN | 25 | macr/ria , serum urea>50 mg/dl and Cr.>1 mg/dl |  | - | 37 | non-diabetics |  |  | HT-C |
|  | Chang (2003) (38) | E. Asians | T2DM | DN | 129 | ESRD |  |  | 116 | non-diabetics matched for age, gender, blood-pressure |  |  | HT-C |
|  | De Cosmo (2002) (39) | Caucasians | T1DM | DN | 75 | pers. micro/macroalbuminuria | 122 | pers. norm/ria , DM duration>15 yrs |  | - |  |  | DC-C |
|  | Fradin (2002) (40) | Caucasians | T2DM | DN | 117 | pers. micro/macroalbuminuria | 118 | pers. norm/ria |  | - |  |  | DC-C |
|  | Nakajima (2002) (41) | E. Asians | T2DM | DN | 132 | micro/macroalbuminuriaexcluding Cr. level ≥ 2.0 mg/dl | 269 | pers. norm/ria |  | - |  |  | DC-C |
|  | Araz (2001) (42) | Asians (Turkey) | T2DM | DN | 116 | pers. micro/macroalbuminuria | 123 | pers. norm/ria | 138 | non-diabetics matched for age and gender |  |  | DC-C, HT-DC-C, HT-C |
|  | Azar (2001) (43) | Caucasians | T1DM | DN | 52 | pers. micro/macroalbuminuriaor ESRD on dialysis | 10 | DM>15 yrs with norm/ria | 27 |  |  |  | DC-C, HT-DC-C, HT-C |
|  | Viswanathan (2001) (44) | Asians | T2DM | DN | 86 | pers. proteinuria, hypertension, DR, normal urine microscopy | 23 | pers. norm/ria , normal urine microscopy without hypertension and retinopathy matched for gender, age, BMI, DM duration and hypertension, HbA1 values |  | - |  |  | DC-C |
|  | Hadjadj (2001) (45) | Caucasians | T1DM | DN | 59 | pers. micro/macroalbuminuria | 251 | pers. norm/ria without hypertension |  | - |  |  | DC-C |
|  | Thomas (2001) (46) | E. Asians | T2DM | DN | 51 | macr/ria | 255 | norm/ria | 111 | non-diabetics |  |  | DC-C, HT-DC-C, HT-C |
|  | Gohda (2001) (47) | E. Asians | T2DM | DN | 536 | micr/ria, proteinuria, CRF, ESRD | 212 | norm/ria |  | - |  |  | DC-C |
|  | Wu (2000) (48) | E. Asians | T2DM | DN | 71 | early-onset DN or clinical proteinuria or renal failure | 41 | norm/ria | 61 | non-diabetics |  |  | DC-C, HT-DC-C, HT-C |
|  | van Ittersum (2000) (49) | Caucasians | T1DM | DN | 69 | pers. micro/macroalbuminuria | 188 | pers. norm/ria |  |  |  |  | DC-C |
|  | Hsieh (2000) (50) | E. Asians | T2DM | DN | 179 | overt proteinuria (>500 mg/day), with or without elevated ser. Cr., hypertension, under or on dilaysis |  |  | 263 | non-diabetics matched for age |  |  | HT-C |
|  | De Cosmo (1999) (51) | Caucasians | T1DM | DN | 175 | DM>10 yrs, pers. micro/macroalbuminuria, DR | 136 | DM>15 yrs, pers. norm/ria | 200 | non-diabetics |  |  | DC-C, HT-DC-C, HT-C |
|  | Miura (1999) (52) | E. Asians | T1DM | DN | 32 | UAE>220 mg/g. Cr with increased s. Cr.,  ESRD, or hemodialysis, CAPD or renal  transplantation (pers. UAE>200 mg/min) | 103 | UAE<12 mg/g. Cr (pers. UAE<10 mg/min) matched for gender, age, DM onset age |  |  |  |  | DC-C |
|  | Vleming (1999) (53) | Caucasians | T1DM | DN | 79 | ESRD due to DN, recipeints of pancreas and kidney mostly | 82 | pers. norm/ria without ACE inhibition |  |  |  |  | DC-C |
|  | Tomino (1999) (54) | E. Asians | T2DM | DN | 745 | micr/ria/ overt nephropathy | 407 | DM>10 yrs, norm/ria |  |  |  |  | DC-C |
|  | Freire (1998) (55) | Asians | T1DM | DN | 77 | pers. micro/macroalbuminuria, DM≥10 yrs | 89 | pers. norm/ria , DM duration≥10 yrs |  |  |  |  | DC-C |
|  | Grzeszczak (1998) (56) | Caucasians | T2DM | DN | 462 | pers. micro/macroalbuminuria | 254 | pers. norm/ria |  |  |  |  | DC-C |
|  | Young (1998) (57) | E. Asians | T2DM | DN | 20 | pers. micro/macroalbuminuria | 54 | pers. norm/ria | 183 | non-diabetics |  |  | DC-C, HT-DC-C, HT-C |
|  | Hanyu (1998) (58) | E. Asians | T2DM | DN | 24 | micr/ria, DR | 21 | norm/ria , DR matched for DM duration, HbA1c, blood pressure |  |  |  |  | DC-C |
|  | Huang (1998) (59) | Caucasians | T2DM | DN | 13 | pers. micr/ria | 46 | pers. norm/ria |  |  |  |  | DC-C |
|  | Kimura (1998) (60) | E. Asians | T2DM | DN | 98 | overt proteinuria, impaired renal function, DR or ESRD requiring dialysis |  | - | 177 | non-diabetics |  |  | HT-C |
|  | Ringel (1997) (61) | Caucasians | T1DM | DN | 134 | pers. micro/macroalbuminuria | 226 | pers. norm/ria |  |  |  | No | DC-C |
|  | Ringel (1997) (61) | Caucasians | T2DM | DN | 161 | pers. micro/macroalbuminuria | 140 | pers. norm/ria |  |  |  |  | DC-C |
|  | Barnas (1997) (62) | Caucasians | T1DM | DN | 63 | pers. micr/ria or overt albuminuria or increased s. Cr. or RRT | 59 | pers. norm/ria | 74 | non-diabetics matched for age |  |  | DC-C, HT-DC-C, HT-C |
|  | Marre (1997) (63) | Caucasians | T1DM | DN | 233 | past or present macr/ria with or without anti-HT meds or RRT | 157 | normal kidney function (pers. norm/ria and plasma Cr.<150μmol/liter without anti-HT meds | 346 | non-diabetics matched for age and gender |  |  | DC-C, HT-DC-C, HT-C |
|  | Schmidt (1997) (64) | Caucasians | T2DM | DN | 61 | DM≥20 yrs, dialysis | 149 | DM≥20 yrs, norm/ria and no hypertension |  |  |  |  | DC-C |
|  | Hibberd (1997) (65) | Caucasians | T1DM | DN | 72 | pers. macr/ria , DR |  | - | 90 | non-diabetics |  |  | HT-C |
|  | Ha (1997) (66) | E. Asians | T2DM | DN | 70 | DM≥10 yrs, macr/ria and hypertension |  | - | 146 | non-diabetics |  |  | HT-C |
|  | Gutiérrez (1997) (66) | Caucasians | T2DM | DN | 60 | pers. micro/macroalbuminuria | 100 | norm/ria matched for gender, age, BMI, DM duration, HbAlc, lipidic profile | 90 | non-diabetics |  |  | Alleles DC-C/ HT-C |
|  | Chowdhury (1996) (67) | Caucasians | T1DM | DN | 242 | pers. macr/ria , DR without hypertension | 166 | pers. norm/ria , DM duration≥15 yrs | 187 | non-diabetics matched for ethnicity |  |  | DC-C, HT-DC-C, HT-C |
|  | Nakajima (1996) (68) | E. Asians | T2DM | DN | 101 | pers. micro/macroalbuminuria, retinopathy | 41 | pers. norm/ria matched for age, DM duration, HbA1c |  |  |  |  | DC-C |
|  | Ohno (1996) (69) | E. Asians | T2DM | DN | 79 | pers. micro/macroalbuminuria | 53 | pers. norm/ria | 74 | non-diabetics |  |  | DC-C, HT-DC-C, HT-C |
|  | Schmidt (1995) (70) | Caucasians | T1DM | DN | 114 | pers. micr/ria, DM duration>10 yrs | 133 | pers. norm/ria , DM duration>10 yrs |  |  |  |  | DC-C |
|  | Schmidt (1995) (70) | Caucasians | T2DM | DN | 247 | pers. micr/ria, DM duration>10 yrs | 208 | pers. norm/ria , DM duration>10 yrs |  |  |  |  | DC-C |
|  | Mizuiri (1995) (71) | E. Asians | T2DM | DN | 80 | micr/ria with renal biopsis/macr/ria and advanced DR before proteinuria | 31 | DM duration≥10 yrs and norm/ria matched for age | 76 |  |  |  | DC-C, HT-DC-C, HT-C |
|  | Panagiotopoulos (1995) (72) | Caucasians | T2DM | DN | 50 | pers. micro/macroalbuminuria | 115 | pers. norm/ria matched for arterial pressure, age, DM duration | 51 | non-diabetics matched for arterial pressure, age, DM duration |  | Νο | DC-C, HT-DC-C, HT-C |
|  | Tarnow (1995) (73) | Caucasians | T1DM | DN | 198 | pers. macr/ria , retinopathy | 190 | pers. norm/ria matched for gender, age, DM duration |  |  |  | Νο | DC-C |
|  | Dudley (1995) (74) | Caucasians | T2DM | DN | 158 | pers. micr/ria | 158 | pers. norm/ria matched for age at DM diagnosis, gender, HbA1c, triglycerides |  |  |  |  | DC-C |
|  | Marre (1994) (63) | Caucasians | T1DM | DN | 62 | pers. micr/ria without permanent hypertension or pers. macr/ria with DR | 62 | pers. norm/ria matched for gender, age, DM duration, DR severity |  |  |  |  | Alleles DC-C |
|  | Luo (2019) (75) | East Asians | T2DM | DN | 650 | pers. macr/ria | 580 | pers. norm/ria |  |  |  |  | DC-C |
|  |  |  |  |  |  |  |  |  |  |  |  |  |  |
| ACE rs4311 | Currie (2010) (18) | Caucasians | T1DM | DN | 718 | pers. proteinuria>0.5 g/24h, hypertension with/without meds, retinopathy | 749 | DM≥15 yrs, norm/ria, no anti-HT meds |  | - |  | No | DC-C |
|  | Ahluwalia (2009) (20) | Asians | T2DM | DN | 240 | pers. macr/ria or CRF without microscopic hematuria | 200 | pers. norm/ria, no anti-HT meds matched for age and ethnnicity |  | - |  |  | DC-C |
|  | Osawa (2007) (76) | East Asians | T2DM | DN | 747 | pers. macr/ria | 557 | pers. norm/ria |  |  |  |  | DC-C |
|  | Hadjadj (2007) (27) | Caucasians | T1DM | DN | 380 | pers. albuminuria with or without renal failure | 382 | pers. norm/ria and no ACE inhibitors or angiotensin receptor blockers |  |  |  |  | Alleles DC-C |
|  | Hadjadj (2007) (27) | Caucasians | T1DM | DN | 385 | pers. albuminuria with or without renal failure | 468 | pers. norm/ria and no ACEi or ARBs |  |  |  |  | Alleles DC-C |
|  | Hadjadj (2007) (27) | Caucasians | T1DM | DN | 277 | pers. albuminuria with or without renal failure | 273 | pers. norm/ria and no ACEi or ARBs |  |  |  |  | Alleles DC-C |
|  |  |  |  |  |  |  |  |  |  |  |  |  |  |
| ACE rs1800764, rs4366, rs12449782 | Hadjadj (2007) (27) | Caucasians | T1DM | DN | 380 | pers. albuminuria with or without renal failure | 382 | pers. norm/ria and no ACE inhibitors or angiotensin receptor blockers |  |  |  |  | Alleles DC-C |
|  | Hadjadj (2007) (27) | Caucasians | T1DM | DN | 385 | pers. albuminuria with or without renal failure | 468 | pers. norm/ria and no ACEi or ARBs |  |  |  |  | Alleles DC-C |
|  | Hadjadj (2007) (27) | Caucasians | T1DM | DN | 277 | pers. albuminuria with or without renal failure | 273 | pers. norm/ria and no ACEi or ARBs |  |  |  |  | Alleles DC-C |
|  |  |  |  |  |  |  |  |  |  |  |  |  |  |
| ADCY1 (rs17489793, rs34154333), AKT3 rs1121276, CACNA1E rs11579714, CACNG2 rs11913713, CALM2 rs1027478, FBN1 (rs16961261, rs16961269), IL12RB1 rs372889, KRAS rs10842508, MAPK10 (rs6827098, rs1460753), ROR1 rs7521581, THSD4 rs7174056 | McDonough (2011)(77) | African Americans | T2DM | ESRD | 965 | pers. albuminuria | 1029 | pers. norm/ria |  |  |  |  | Alleles DC-C |
|  | McDonough (2011) (77) | African Americans | T2DM | ESRD | 965 | pers. albuminuria | 1029 | pers. norm/ria |  |  |  |  | Alleles DC-C |
|  |  |  |  |  |  |  |  |  |  |  |  |  |  |
| AGT M235T (rs699) | Makuc (2017) (78) | Caucasians | T2DM | DN | 276 | pers. micr/macroalbuminuria, DM≥10yrs | 375 | pers. norm/ria, DM>10yrs |  |  |  |  | DC-C |
|  | Wang (2016) (4) | Asians | T2DM | DN | 301 | Pers. micr/macroalbuminuria | 497 | pers. norm/ria |  |  |  |  | DC-C |
|  | Ilic (2014) (79) | Caucasians | T1DM | DN | 46 | pers. micro/macroalbuminuria | 33 | pers. norm/ria |  | - |  | No | DC-C |
|  | Reis (2011) (80) | Asians (Turkey) | T2DM |  | 111 | pers. macr/ria | 108 | pers. norm/ria | 206 | non-diabetics |  |  | DC-C, HT-DC-C, HT-C |
|  | Zsom (2011) (15) | Caucasians | T1DM | d. ESRD | 21 | d. ESRD |  | - | 200 | non-diabetics matched for age |  |  | HT-C |
|  | Zsom (2011) (15) | Caucasians | T2DM | d. ESRD | 93 | d. ESRD |  | - |  | non-diabetics matched for age |  |  | HT-C |
|  | Mtiraoui (2011) (81) | Africans (Tunisia) | T2DM |  | 512 | pers. micro/macroalbuminuria | 405 | pers. norm/ria matched for gender, age, BMI, waist-to-hip ratio, prevalence of hypertension, age at disease onset |  |  |  | No | DC-C |
|  | Ahluwalia (2009) (20) | Asians | T2DM | DN | 240 | pers. macr/ria or CRF without microscopic hematuria | 200 | pers. norm/ria excluding micr/ria and those on anti-HT treatment matched for age and ethnnicity |  | - |  |  | DC-C |
|  | Mollsten (2008) (25) | Caucasians | T1DM | DN | 121 | pers. micro/macroalbuminuria | 197 | DM≥20 yrs, norm/ria , no anti-HT meds |  | - |  |  | DC-C |
|  | Osawa (2007) (76) | E. Asians | T2DM | DN | 747 | overt nephropathy and DR | 557 | norm/ria and DR |  | - |  | No | DC-C |
|  | Prasad (2006) (31) | Asians | T2DM | diabetic CRI | 196 | DM duration≥2 years, moderate CRI (serum Cr. ≥3 mg/dl, macr/ria , DR) | 225 | pers. norm/ria , DM≥10 yrs |  | - |  | No | DC-C |
|  | Chang (2003) (82) | E. Asians | T2DM | DN | 129 | ESRD |  |  | 116 | non-diabetics matched for age, gender, blood-pressure |  |  | HT-C |
|  | Fradin (2002) (40) | Caucasians | T2DM | DN | 117 | pers. micro/macroalbuminuria | 118 | pers. norm/ria |  | - |  |  | DC-C |
|  | Thomas (2001) (46) | E. Asians | T2DM | DN | 51 | macr/ria | 255 | norm/ria | 111 | non-diabetics |  |  | DC-C, HT-DC-C, HT-C |
|  | Wu (2000) (48) | E. Asians | T2DM | DN | 71 | early-onset DN or clinical proteinuria or renal failure | 41 | norm/ria | 61 | non-diabetics |  | No | DC-C, HT-DC-C, HT-C |
|  | Zychma (2000) (83) | Caucasians | T2DM | DN | 450 | pers. micr/ria/overt nephropathy/CRF | 243 | pers. norm/ria |  |  |  |  | DC-C |
|  | van Ittersum (2000) (49) | Caucasians | T1DM | DN | 69 | pers. micro/macroalbuminuria | 188 | pers. norm/ria |  |  |  |  | DC-C |
|  | Miura (1999) (52) | E. Asians | T1DM | DN | 32 | UAE>220 mg/g. Cr with increased serum Cr.,  ESRD, or hemodialysis, CAPD or renal  transplantation (pers. UAE>200 mg/min) | 103 | UAE<12 mg/g. Cr (pers. UAE<10 mg/min) matched for gender, age, DM onset age |  |  |  |  | DC-C |
|  | Young (1998) (57) | E. Asians | T2DM | DN | 20 | pers. micro/macroalbuminuria | 54 | pers. norm/ria | 183 | non-diabetics |  |  | DC-C, HT-DC-C, HT-C |
|  | Freire (1998) (84) | Caucasians | T2DM | DN | 117 | DM duration≥10 yrs, pers. proteinuria | 125 | DM duration≥10 yrs, pers. norm/ria |  |  |  |  | DC-C |
|  | Ringel (1997) (61) | Caucasians | T1DM | DN | 134 | pers. micro/macroalbuminuria | 226 | pers. norm/ria |  |  |  |  | DC-C |
|  | Ringel (1997) (61) | Caucasians | T2DM | DN | 161 | pers. micro/macroalbuminuria | 140 | pers. norm/ria |  |  |  |  | DC-C |
|  | Marre (1997) (63) | Caucasians | T1DM | DN | 233 | past or present macr/ria with or without anti-HT meds or RRT | 157 | normal kidney function (pers. norm/ria and plasma Cr.<150μmol/liter without anti-HT treatment | 346 | non-diabetics matched for age and gender |  |  | DC-C |
|  | Chowdhury (1996) (67) | Caucasians | T1DM | DN | 242 | pers. macr/ria , DR without other kidney or urinary tract disease and hypertension | 166 | pers. norm/ria , DM duration≥15 yrs | 187 | non-diabetics matched for ethnicity |  |  | DC-C, HT-DC-C, HT-C |
|  | Ohno (1996) (69) | E. Asians | T2DM | DN | 79 | pers. micro/macroalbuminuria | 53 | pers. norm/ria | 74 | non-diabetics |  |  | DC-C, HT-DC-C, HT-C |
|  | Schmidt (1996) (85) | Caucasians | T1DM | DN | 180 | DM duration≥10 yrs, pers. micr/ria with or without elevated s. Cr. or dialysis | 243 | DM duration≥10 yrs, pers. norm/ria | 230 | non-diabetics |  | No | DC-C, HT-DC-C, HT-C |
|  | Schmidt (1996) (85) | Caucasians | T2DM | DN | 310 | DM duration≥10 yrs, pers. micr/ria with or without elevated s. Cr. or dialysis | 353 | DM duration≥10 yrs, pers. norm/ria | 230 | non-diabetics |  | Yes | DC-C, HT-DC-C, HT-C |
|  | Fogarty (1996) (86) | Caucasians | T1DM | DN | 95 | DM duration≥10 yrs, pers. proteinuria with or without impaired renal function or ESRD | 100 | DM duration≥20 yrs, pers. norm/ria matched for age of onset, DM duration, and age at recruitment | 80 | non-diabetics |  | No | DC-C, HT-DC-C, HT-C |
|  | Tarnow (1996) (87) | Caucasians | T1DM | DN | 195 | DN | 185 | pers. norm/ria matched for age, gender, DM duration |  |  |  |  | DC-C |
|  |  |  |  |  |  |  |  |  |  |  |  |  |  |
| AGT T174M (rs4762) | Makuc (2017) (78) | Caucasians | T2DM | DN | 276 | pers. micr/macroalbuminuria, DM≥10yrs | 375 | pers. norm/ria, DM>10yrs |  |  |  |  | DC-C |
|  | Mtiraoui (2011) (81) | Africans (Tunisia) | T2DM |  | 512 | pers. micro/macroalbuminuria | 405 | pers. norm/ria matched for gender, age, BMI, waist-to-hip ratio, prevalence of hypertension, age at disease onset |  |  |  | Yes | DC-C |
|  | Ahluwalia (2009) (20) | Asians | T2DM | DN | 240 | pers. macr/ria or CRF without microscopic hematuria | 200 | pers. norm/ria excluding micr/ria and those on anti-HT treatment matched for age and ethnnicity |  | - |  | Yes | DC-C |
|  | Prasad (2006) (31) | Asians | T2DM | diabetic CRI | 196 | DM duration≥2 years, moderate CRI (serum Cr. ≥3 mg/dl, macr/ria , DR) | 225 | pers. norm/ria , DM≥10 yrs |  | - |  | Yes | DC-C |
|  | Wu (2000) (48) | E. Asians | T2DM | DN | 71 | early-onset DN or clinical proteinuria or renal failure | 41 | norm/ria | 61 | non-diabetics |  | Yes | DC-C, HT-C |
|  | Marre (1997) (63) | Caucasians | T1DM | DN | 233 | past or present macr/ria with or without anti-HT meds or RRT | 157 | normal kidney function (pers. norm/ria and plasma Cr.<150μmol/liter without anti-HT treatment | 346 | non-diabetics matched for age and gender |  | Yes | DC-C |
|  | Tarnow (1996) (87) | Caucasians | T1DM | DN | 195 | DN | 185 | pers. norm/ria matched for age, gender, DM duration |  |  |  | Yes | DC-C |
|  | Vázquez-Moreno (2021) (88) |  |  |  |  |  |  |  |  |  |  |  |  |
|  | Chang (2003) (38) | E. Asians | T2DM | DN | 129 | ESRD |  |  | 116 | non-diabetics matched for age, gender, blood-pressure |  |  | HT-C |
|  |  |  |  |  |  |  |  |  |  |  |  |  |  |
| AGT rs699, rs2478522, rs3827749 | Tregouet (2008) (89) | Caucasians | T1DM | DN | 489 | pers. macr/ria | 463 | pers. norm/ria , DM≥15 yrs |  |  |  |  | Alleles DC-C, HT-C |
|  | Tregouet (2008) (89) | Caucasians | T1DM | DN | 387 | pers. macr/ria | 469 | pers. norm/ria , DM≥15 yrs |  |  |  |  | Alleles DC-C, HT-C |
|  | Tregouet (2008) (89) | Caucasians | T1DM | DN | 300 | pers. macr/ria | 391 | pers. norm/ria , DM≥15 yrs |  |  |  |  | Alleles DC-C, HT-C |
|  |  |  |  |  |  |  |  |  |  |  |  |  |  |
| AGTR1 A1166C (rs5186) | Ali (2018) (90) | Malay | T2DM | DN | 80 | Pers. micr/macroalbuminuria | 40 | Pers. normoalbuminuria |  |  |  |  | DC-C |
|  | Moradi (2015) (91) | Asians | T2DM | DN | 94 | pers. micro/macroalbuminuria | 41 | pers. norm/ria matched for gender | 98 | non-diabetics matched for gender |  |  | DC-C, HT-DC-C, HT-C |
|  | Ilic (2014) (8) | Caucasians | T1DM | DN | 46 | pers. micro/macroalbuminuria | 33 | pers. norm/ria |  | - |  |  | DC-C |
|  | Shah (2013) (92) | Asian Indians | T2DM | DN | 240 | macr/ria | 255 | DM≥5 yrs, pers. norm/ria matched for age, ethnicity |  | - |  | No | DC-C |
|  | Shah (2013) (92) | Asian Indians | T2DM | DN | 260 | macr/ria | 215 | DM≥5 yrs, pers. norm/ria matched for age, ethnicity |  | - |  | No | DC-C |
|  | Shah (2013) (92) | Asian Indians | T2DM | DN | 96 | macr/ria | 92 | DM≥5 yrs, pers. norm/ria matched for age, ethnicity |  | - |  |  | DC-C |
|  | Mollsten (2011) (93) | Caucasians | T1DM | DN | 359 | pers. micr/ria | 577 | DM>15 yrs, pers. norm/ria , no anti-HT meds |  |  |  |  | DC-C |
|  | Mollsten (2011) (93) | Caucasians | T1DM | DN | 325 | pers. micr/ria | 665 | DM>15 yrs, pers. norm/ria , no anti-HT meds |  |  |  |  | DC-C |
|  | Mollsten (2011) (93) | Caucasians | T1DM | DN | 880 | overt nephropathy |  |  |  |  |  |  | DC-C |
|  | Mollsten (2011) (93) | Caucasians | T1DM | DN | 610 | overt nephropathy |  |  |  |  |  |  | DC-C |
|  | Zsom (2011) (15) | Caucasians | T1DM | d. ESRD | 21 | d. ESRD |  | - | 200 | non-diabetics matched for age |  |  | HT-C |
|  | Zsom (2011) (15) | Caucasians | T2DM | d. ESRD | 93 | d. ESRD |  | - |  | non-diabetics matched for age |  |  | HT-C |
|  | Currie (2010) (18) | Caucasians | T1DM | DN | 718 | pers. proteinuria>0.5 g/24h, hypertension with/without meds, retinopathy | 749 | DM≥15 yrs, norm/ria with no anti-HT meds |  | - |  |  | DC-C |
|  | Ahluwalia (2009) (20) | Asians | T2DM | DN | 240 | pers. macr/ria or CRF without microscopic hematuria | 200 | pers. norm/ria excluding micr/ria and those on anti-HT treatment matched for age and ethnnicity |  | - |  | No | DC-C |
|  | Mollsten (2008) (94) | Caucasians | T1DM | DN | 121 | pers. micro/macroalbuminuria | 197 | DM≥20 yrs, norm/ria , no anti-HT meds |  | - |  |  | DC-C |
|  | Osawa (2007) (76) | E. Asians | T2DM | DN | 747 | overt nephropathy and DR | 557 | norm/ria and DR |  | - |  |  | DC-C |
|  | Prasad (2006) (31) | Asians | T2DM | diabetic CRI | 196 | DM duration≥2 years, moderate CRI (serum Cr. ≥3 mg/dl, macr/ria , DR) | 225 | pers. norm/ria , DM≥10 yrs |  | - |  |  | DC-C |
|  | Fradin (2002) (40) | Caucasians | T2DM | DN | 117 | pers. micro/macroalbuminuria | 118 | pers. norm/ria |  | - |  |  | DC-C |
|  | Thomas (2001) (46) | E. Asians | T2DM | DN | 51 | macr/ria | 255 | norm/ria | 111 | non-diabetics |  |  | DC-C, HT-DC-C, HT-C |
|  | Wu (2000) (48) | E. Asians | T2DM | DN | 71 | early-onset DN or clinical proteinuria or renal failure | 41 | norm/ria | 61 | non-diabetics |  |  | DC-C, HT-DC-C, HT-C |
|  | van Ittersum (2000) (49) | Caucasians | T1DM | DN | 69 | pers. micro/macroalbuminuria | 188 | pers. norm/ria |  |  |  |  | DC-C |
|  | Savage (1999) (95) | Caucasians | T1DM | DN | 95 | DM duration>10 yrs, pers. proteinuria | 97 | DM>20 yrs, pers. norm/ria , no anti-HT treatment |  |  |  |  | DC-C |
|  | Young (1998) (57) | E. Asians | T2DM | DN | 20 | pers. micro/macroalbuminuria | 54 | pers. norm/ria | 183 | non-diabetics |  |  | DC-C, HT-DC-C, HT-C |
|  | Marre (1997) (63) | Caucasians | T1DM | DN | 233 | past or present macr/ria with or without anti-HT meds or RRT | 157 | normal kidney function (pers. norm/ria and plasma Cr.<150μmol/liter without anti-HT treatment | 346 | non-diabetics matched for age and gender |  |  | DC-C |
|  | Doria (1997) (96) | Caucasians | T1DM | DN | 73 | micro/macroalbuminuria | 79 | DM≥15 yrs, normoambuminuria |  |  |  |  | DC-C |
|  | Chowdhurry (1997) (97) | Caucasians | T1DM | DN | 264 | pers. proteinuria, retinopathy and hypertension |  | - | 212 | non-diabetics |  |  | HT-C |
|  | Tarnow (1996) (98) | Caucasians | T1DM | DN | 198 | DN | 190 | pers. norm/ria matched for gender, age, DM duration |  |  |  |  | DC-C |
|  | Vionnet (2006) (99) | Caucasians | T1DM | DN | 390 | pers. albuminuria | 385 | pers. norm/ria , DM≥15yrs |  |  |  |  | Alleles DC-C |
|  | Vionnet (2006) (99) | Caucasians | T1DM | DN | 387 | pers. albuminuria | 469 | pers. norm/ria , DM≥15yrs |  |  |  |  | Alleles DC-C |
|  | Vionnet (2006) (99) | Caucasians | T1DM | DN | 280 | pers. albuminuria | 273 | pers. norm/ria , DM≥15yrs |  |  |  |  | Alleles DC-C |
|  |  |  |  |  |  |  |  |  |  |  |  |  |  |
| AGTR1 (rs1492078, rs275653, rs2276736, rs1800766, rs5182), IL12A (rs583911, rs2243135, IL12A_6489, rs2243136, rs568408), THPO (rs956732, rs6141, rs6142) | Vionnet (2006) (99) | Caucasians | T1DM | DN | 390 | pers. albuminuria | 385 | pers. norm/ria , DM≥15yrs |  |  |  |  | Alleles DC-C |
|  | Vionnet (2006) (99) | Caucasians | T1DM | DN | 387 | pers. albuminuria | 469 | pers. norm/ria , DM≥15yrs |  |  |  |  | Alleles DC-C |
|  | Vionnet (2006) (99) | Caucasians | T1DM | DN | 280 | pers. albuminuria | 273 | pers. norm/ria , DM≥15yrs |  |  |  |  | Alleles DC-C |
|  |  |  |  |  |  |  |  |  |  |  |  |  |  |
|  | Tregouet (2008) (89) | Caucasians | T1DM | DN | 489 | pers. macr/ria | 463 | pers. norm/ria , DM≥15 yrs |  |  |  |  | Alleles DC-C |
|  | Tregouet (2008) (89) | Caucasians | T1DM | DN | 387 | pers. macr/ria | 469 | pers. norm/ria , DM≥15 yrs |  |  |  |  | Alleles DC-C |
|  | Tregouet (2008) (89) | Caucasians | T1DM | DN | 300 | pers. macr/ria | 391 | pers. norm/ria , DM≥15 yrs |  |  |  |  | Alleles DC-C |
|  |  |  |  |  |  |  |  |  |  |  |  |  |  |
| CDKN2A/B rs10811661 | Fagerholm (2012) (100) | Caucasians | T1DM | DN | 1320 | pers. miicro/macr/ria or ESRD | 1234 | DM≥15 yrs, pers. norm/ria |  |  |  |  | Alleles DC-C |
|  | Fagerholm (2012) (100) | Caucasians | T1DM | DN | 747 | pers. miicro/macr/ria or ESRD | 808 | DM≥15 yrs, pers. norm/ria |  |  |  |  | Alleles DC-C |
|  | Fagerholm (2012) (100) | Caucasians | T1DM | DN | 516 | pers. miicro/macr/ria or ESRD | 376 | DM≥15 yrs, pers. norm/ria |  |  |  |  | Alleles DC-C |
|  | Fagerholm (2012) (100) | Caucasians | T1DM | DN | 231 | pers. miicro/macr/ria or ESRD | 298 | DM≥15 yrs, pers. norm/ria |  |  |  |  | Alleles DC-C |
|  |  |  |  |  |  |  |  |  |  |  |  |  |  |
| EPO rs1617640 | Tong (2008) – Utah cohort (101) | Caucasians | T2DM |  | 374 | ESRD | 239 | norm/ria , DM≥15 yrs, matched age, ethnicity |  |  |  |  | DC-C |
|  | Tong (2008) - GoKinD cohort A (101) | Caucasians | T1DM |  | 365 | ESR | 574 | norm/ria |  |  |  |  | DC-C |
|  | Tong (2008) - Boston cohort (101) | Caucasians | T1DM |  | 379 | nephropathy (macr/ria or ESRD) | 141 | norm/ria , DM duration≥15 yrs |  |  |  |  | DC-C |
|  |  |  |  |  |  |  |  |  |  |  |  |  |  |
| GREM1 rs1129456, rs3207357 | McKnight (2010) – Initial Study (102) | Caucasians | T1DM | DN | 267 | nephropathy | 442 | norm/ria |  |  |  |  | DC-C |
|  | McKnight (2010) – Replication Study (102) | Caucasians | T1DM | DN | 597 | nephropathy | 502 | norm/ria |  |  |  |  | DC-C |
|  |  |  |  |  |  |  |  |  |  |  |  |  |  |
| IL1B -511C/T | Buraczynska (2019) (103) | Caucasians | T2DM | DN | 506 | nephropathy | 354 | norm/ria | 505 | Healthy controls |  |  | DC-C |
|  | Stefanidis (2014) (104) | Caucasians | T2DM | DN | 173 | nephropathy (macr/ria or ESRD) | 186 | Persistent norm/ria |  |  |  |  | DC-C |
|  | Lee (2004) (105) | Asians | T2DM | DN |  |  |  |  |  |  |  |  | DC-C |
|  |  |  |  |  |  |  |  |  |  |  |  |  |  |
| IL10 -1082 A>G (rs1800896) | Erdogan (2012) (106) | Asians (Turkey) | T2DM | DN | 43 | With nephropathy | 48 | Without nephropathy | 112 | healthy | No | No | DC-C, HT-DC-C, HT-C |
|  | Kung (2010) (107) | E. Asians | T2DM | DN | 24 | DN | 23 | normal renal funcrion, matched age, gender, ethnicity | 25 | Healthy, matched age, gender, ethnicity | No | No | DC-C, HT-DC-C, HT-C |
|  | Mtiraoui (2009) (108) | Africans | T2DM | DN | 515 | pers. micr/ria, p. Cr.>176 μmol/L | 402 | pers. norm/ria , matched gender, age | 748 | Healthy, matched gender, age |  |  | DC-C, HT-DC-C, HT-C |
|  | Babel (2006) (109) | Caucasians | T2DM | ESRD | 44 | d. ESRD |  | - | 118 | healthy | No |  | HT-C |
|  | Yin (2015) (110) | E. Asians | T2DM | DN | 172 | DN |  |  | 344 | Healthy controls |  |  | HT-C |
|  | Polina (2017) (111) | Caucasians | T2DM | DN | 126 | DN | 249 | pers. norm/ria |  |  |  |  | DC-C |
|  |  |  |  |  |  |  |  |  |  |  |  |  |  |
| IL10  -592 C>A rs1800872 | Arababadi (2012) (112) | Asians | T2DM | DN | 100 | overt albuminuria | 100 | pers. norm/ria , matched DM duration, gender, age, socioeconomic status | 100 | Healthy, matched DM duration, gender, age, socioeconomic status |  |  | DC-C, HT-DC-C, HT-C |
|  | Kung (2010) (107) | E. Asians | T2DM | DN | 24 | DN | 23 | normal renal funcrion, matched age, gender, ethnicity | 25 | Healthy, matched age, gender, ethnicity | No | No | DC-C, HT-DC-C, HT-C |
|  | Mtiraoui (2009) (108) | Africans | T2DM | DN | 515 | pers. micr/ria, p. Cr.>176 μmol/L | 402 | pers. norm/ria , matched gender, age | 748 | Healthy, matched gender, age |  |  | DC-C, HT-DC-C, HT-C |
|  | Yin (2015) (113) | East Asians | T2DM | DN | 172 | DN |  |  | 344 | Healthy controls |  |  | HT-C |
|  |  |  |  |  |  |  |  |  |  |  |  |  |  |
| IL1RN  86 bp VNTR IL1RN*1>2/3/4 | Loughrey (1998) (114) | Caucasians | IDDM | DN | 95 (NR) | proteinuria (≥300mg/24h), DM≥10yrs | 96 | albuminuria<20μg/min, DM>20 yrs, matched age at onset of DM and DM duration | 210 |  |  |  | DC-C |
|  | Blakemore (1996) (115) | Caucasians | T1DM | DN | 23 | Pers. proteinuria and impaired renal function | 125 | Without complications | 261 | Healthy, matched ethnicity |  |  | DC-C |
|  | Blakemore (1996) (115) | Caucasians | T2DM |  | 17 | Pers. proteinuria and impaired renal function | 120 | Without complications |  |  |  |  | DC-C |
|  |  |  |  |  |  |  |  |  |  |  |  |  |  |
| IL6 G(-174)C rs1800795 | Abrahamian (2007) (116) | Caucasians | T2DM | DN | 44 | pers. macr/ria | 66 | pers. norm/ria |  |  |  |  | DC-C |
|  | Papaoikonomou (2013) (117) | Caucasians | T2DM | DN | 59 | pers. macr/ria | 240 | pers. norm/ria |  |  |  | No | DC-C |
|  |  |  |  |  | 94 | pers. micr/ria |  |  |  |  |  |  |  |
|  | Ng (2008) (118) | Caucasians |  |  | 295 | Pers. proteinuria or CRF or d. ESRD | 174 | Pers. norm/ria, DM≥7 yrs, Comparable gender composition, age at DM diagnosis, HbA1c |  |  |  |  | DC-C |
|  |  |  |  |  |  |  |  |  |  |  |  |  |  |
| ITP3 rs9368768 | Germain (2015) (119) |  |  |  |  |  |  |  |  |  |  |  |  |
|  | Germain (2015) (119) |  |  |  |  |  |  |  |  |  |  |  |  |
|  |  |  |  |  |  |  |  |  |  |  |  |  |  |
| MMP9 rs17576 | **Ahluwalia (2009)** (120) | Asian Indians | T2DM | DN | 240 | macr/ria | 255 | norm/ria |  |  |  |  | DC-C |
|  | **Ahluwalia (2009)** (120) | Asian Indians | T2DM | DN | 96 | macr/ria | 92 | norm/ria |  |  |  |  | DC-C |
|  | Albert (2019) (121) | Caucasians | T2DM | DN | 32 | macr/ria | 162 | norm/ria |  |  |  |  | DC-C |
|  |  |  |  |  |  |  |  |  |  |  |  |  |  |
| MRAS rs9818870 | Horová (2012) (122) | Caucasians | T1DM | DN | 243 | macr/ria | 817 | norm/ria |  |  |  |  | DC-C |
|  | Horová (2012) (122) | Caucasians | T2DM | DN |  |  |  |  |  |  |  |  | DC-C |
|  |  |  |  |  |  |  |  |  |  |  |  |  |  |
| NOS2 (AAAT)4>5/6 | Liao (2006) (123) | Asians | T2DM | DN | 81 | macr/ria with or without elevated s.Cr. | 65 | norm/ria , normal s. Cr. levels |  |  |  |  | DC-C |
|  | Liao (2006) (123) | Asians | T2DM | DN | 51 | macr/ria with or without elevated s.Cr. | 20 | norm/ria , normal s. Cr. levels |  |  |  |  | DC-C |
|  | Liao (2006) (123) | Asians | T2DM | DN | 26 | macr/ria with or without elevated s. Cr. | 11 | norm/ria , normal s.Cr. levels |  |  |  |  | DC-C |
|  | Rippin (2003) (124) | Caucasians | T1DM | DN | 464 | macr/ria | 396 | norm/ria |  |  |  |  | DC-C |
|  |  |  |  |  |  |  |  |  |  |  |  |  |  |
| NOS3  Intron 4 b>a | Rahimi (2013) (125) | Asians | T2DM | DN | 121 | pers. micro/macroalbuminuria | 52 | pers. norm/ria | 101 | non-diabetics | Yes |  | DC-C, HT-DC-C, HT-C |
|  | Shoukry (2012) (126) | Africans | T2DM | DN | 200 | pers. macr/ria | 200 | DM≥10 yrs, norm/ria , no anti-HT meds |  |  |  |  | DC-C |
|  | Ahluwalia (2008) (127) | Asian Indians | T2DM | DN | 195 | macr/ria | 255 | DM≥10 yrs, pers. norm/ria <10mg/l, no anti-HT treatment matched for age, gender, ethnicity |  |  |  |  | DC-C |
|  | Ezzidi (2008) (128) | Africans | T2DM | DN | 515 | confirmed DN, AER>30 mg and/or Cr>176 μmol/l | 402 | pers. norm/ria matched for age, gender | 748 | non-diabetics matched for age and gender | Yes |  | DC-C, HT-DC-C, HT-C |
|  | Mollsten (2006) (129) | Caucasians | T1DM | DN | 805 | pers. microabuminuria or macr/ria or ESRD | 358 | DM≥20 yrs, norm/ria , no anti-HT meds |  |  |  |  | DC-C |
|  | Mollsten (2006) (129) | *Caucasians* | *T1DM* | *DN* | 150 | pers. microabuminuria or macr/ria or ESRD | 197 | DM≥20 yrs, norm/ria , no anti-HT meds |  |  |  |  | DC-C |
|  | Rippin (2003) (124) | Caucasians | T1DM | DN | 464 | overt proteinuria, hypertension and DR | 396 | DM≥50 yrs and no nephropathy |  |  |  |  | DC-C |
|  | Shimizu (2002) (130) | E. Asians | T2DM | DN | 107 | overt proteinuria, normal s. Cr. | 203 | DM>10 yrs, normal renal function, norm/ria matched for age and gender | 114 | non-diabetics matched for age and gender | Yes |  | DC-C, HT-DC-C, HT-C |
|  | Shestakova (2006) (32) | Caucasians | T1DM | DN | 63 | pers. macr/ria with or without anti-HT meds | 66 | pers. norm/ria | 96 | non-diabetics |  | No | DC-C |
|  | Neugebauer (2000) (131) | E. Asians | T2DM | DN | 133 | pers. micr/ria/proteinuria, DM≥5 yrs | 82 | pers. norm/ria , DM≥5 yrs | 155 | non-diabetics | Yes |  | DC-C, HT-DC-C, HT-C |
|  | Santos (2009) (132) | Caucasian-Brazilians | T2DM | DN | 376 | pers. micro/macroalbuminuriaor ESRD | 241 | DM≥10 yrs, pers. norm/ria | 100 | non-diabetics | Yes |  | DC-C, HT-DC-C, HT-C |
|  | Degen (2001) (133) | Caucasians | T1DM | DN | 130 | DM≥10 yrs, pers. micr/ria | 194 | pers. norm/ria |  |  |  |  | DC-C |
|  | Degen (2001) (133) | Caucasians | T2DM | DN | 197 | DM≥10 yrs, pers. micr/ria | 217 | pers. norm/ria |  |  |  |  | DC-C |
|  | Fujita (2000) (134) | E. Asians | T2DM | DN | 102 | macr/ria and proliferative DR | 65 | norm/ria and proliferative DR |  |  |  |  | DC-C |
|  | Lin (2002) (135) | E. Asians | T2DM | DN | 80 | DN with or without CRF | 48 |  | 70 | non-diabetics | Yes |  | DC-C, HT-DC-C, HT-C |
|  | Mohseni (2011) (136) | Asians | T2DM | DN | 20 | pers. micr/ria | 71 | norm/ria | 96 | non-diabetics | Yes |  | DC-C, HT-DC-C, HT-C |
|  | Narne (2014) (137) | Asians | T2DM | DN | 155 | pers. macr/ria , DR | 162 | pers. norm/ria |  |  |  |  | DC-C |
|  | Bellini (2007) (138) | Caucasians-Brazilians | NR | DN | 37 | DN |  | - | 94 | non-diabetics |  |  | HT-C |
|  | Nagase (2003) (139) | E. Asians | NR | d. ESRD | 71 | d. ESRD, under dialysis |  | - | 248 | non-diabetics |  | - | HT-C |
|  | Lamnissou (2004) (140) | Caucasians | NR | DN | 77 | d. ESRD |  | - | 295 | non-diabetics |  |  | HT-C |
|  | Asakimori (2001) (141) | E. Asians | NR | ESRD | 295 | d. ESRD |  | - | 189 | non-diabetics |  |  | HT-C |
|  |  |  |  |  |  |  |  |  |  |  |  |  |  |
| **NOS3**  T-786C rs2070744 | Shoukry (2012) (126) | Africans | T2DM | DN | 200 | pers. macr/ria | 200 | DM≥10 yrs, norm/ria , no anti-HT meds |  |  |  |  | DC-C |
|  | Ahluwalia (2008) (127) | Asian Indians | T2DM | DN | 195 | macr/ria | 255 | DM≥10 yrs, pers. norm/ria <10mg/l under no anti-HT treatment matched for age, gender, ethnicity |  |  |  | No | DC-C |
|  | Ezzidi (2008) (128) | Africans | T2DM | DN | 515 | confirmed DN, AER>30 mg and/or Cr>176 μmol/l | 402 | pers. norm/ria matched for age, gender | 748 | non-diabetics matched for age and gender |  |  | DC-C, HT-C |
|  | Santos (2011) (132) | Caucasian-Brazilians | T2DM | DN | 376 | pers. micro/macroalbuminuriaor ESRD | 241 | DM≥10 yrs, pers. norm/ria | 100 | non-diabetics |  |  | DC-C, HT-C |
|  | Narne (2014) (137) | Asians | T2DM | DN | 155 | pers. macr/ria , DR | 162 | pers. norm/ria and negative for dipstick urinary protein |  |  |  |  | DC-C |
|  | Huo (2015) (142) | E. Asians | T2DM | DN | 431 | urinary albumin>500 mg/L and ACR>300 mg/g | 420 | negative dipstick yrinary protein and urinary albumin<10 mg/L |  |  |  |  | DC-C |
|  | Bazaaz (2010) (143) | Caucasians | T1DM | DN | 88 | DM duration>3 yrs, pers. macr/ria |  |  | 104 | non-diabetics |  |  | HT-C |
|  | Zsom (2011) (15) | Caucasians | T1DM | d. ESRD | 21 | d. ESRD |  | - | 200 | non-diabetics matched for age |  |  | HT-C |
|  | Zsom (2011) (15) | Caucasians | T2DM | d. ESRD | 93 | d. ESRD |  | - |  | non-diabetics matched for age |  |  | HT-C |
|  | Asakimori (2002) (144) | E. Asians | ND | DN | 74 | d. ESRD |  | - | 187 | non-diabetics |  |  | HT-C |
|  | Moguib (2017) (145) | Africans | T2DM |  | 200 | pers. proteinuria |  |  | 100 | Healthy |  |  | DC-C |
|  | Raina cohort 1 (146) | Asians | T2DM | DN | 204 | DN | 257 | pers. norm/ria | 315 | Healthy |  |  | DC-C |
|  | Raina cohort 2 (146) | Asians | T2DM | DN | 150 | DN | 187 | pers. norm/ria | 200 | Healthy |  |  | DC-C |
|  |  |  |  |  |  |  |  |  |  |  |  |  |  |
| NOS3 G894T rs1799983 | Rahimi (2012) (11) | Asians | T2DM | DN | 140 | pers. micro/macroalbuminuria | 72 | norm/ria |  |  |  | No | DC-C |
|  | Shin Shin (2004) (36) | E. Asians | T2DM | DN | 118 | overt nephropathy or pers. micr/ria, DM>5 yrs | 59 | norm/ria , DM>5 yrs | 129 | non-diabetics |  |  | DC-C, HT-DC-C, HT-C |
|  | Shoukry (2012) (126) | Africans | T2DM | DN | 200 | pers. macr/ria | 200 | DM≥10 yrs, norm/ria , no anti-HT meds |  |  |  |  | DC-C |
|  | Bessa (2011) (147) | Africans | T2DM | DN | 40 | ESRD, pers. proteinuria,CrCl<15 mL/min, hemodialysis | 40 | norm/ria , normal renal function | 20 | non-diabetics matched for age and gender |  |  | DC-C, HT-DC-C, HT-C |
|  | Mollsten (2009) (148) | Caucasians | T1DM | DN | 458 | pers. macr/ria , DR | 319 | DM≥20 yrs, pers. norm/ria , no anti-HT meds |  |  |  |  | DC-C |
|  | Ahluwalia (2008) (127) | Asian Indians | T2DM | DN | 195 | macr/ria | 255 | DM≥10 yrs, pers. norm/ria <10mg/l under no anti-HT treatment matched for age, gender, ethnicity |  |  |  |  | DC-C |
|  | Ezzidi (2008) (128) | Africans | T2DM | DN | 515 | confirmed DN, AER>30 mg and/or Cr>176 μmol/l | 402 | pers. norm/ria matched for age, gender | 748 | non-diabetics matched for age and gender |  |  | DC-C, HT-DC-C, HT-C |
|  | Tiwari (2009) (149) | Asian Indians | T2DM | Diabetic CRI | 90 | moderate CRI, pers.ly s. Cr≥2 mg/dl, DM≥2 yrs, DR | 75 | DM≥10 yrs and s. Cr<2 mg/dl |  |  |  |  | DC-C |
|  | Tiwari (2009) (149) | Asian Indians | T2DM | Diabetic CRI | 106 | moderate CRI, serum Cr≥2 mg/dl, DM≥2 yrs, DR | 149 | DM≥10 yrs and serum Cr<2 mg/dl with no history of kidney diseases |  |  |  | No | DC-C |
|  | Mollsten (2006) (150) | Caucasians | T1DM | DN | 805 | pers. microabuminuria or macr/ria or ESRD | 358 | DM≥20 yrs, norm/ria , no anti-HT meds |  |  |  |  | DC-C |
|  | Mollsten (2006) (150) | Caucasians | T1DM | DN | 150 | pers. microabuminuria or macr/ria or ESRD | 197 | DM≥20 yrs, norm/ria , no anti-HT meds |  |  |  |  | DC-C |
|  | Santos (2011) (132) | Caucasian-Brazilians | T2DM | DN | 376 | pers. micro/macroalbuminuriaor ESRD | 241 | DM≥10 yrs, pers. norm/ria | 100 | non-diabetics |  |  | DC-C, HT-DC-C, HT-C |
|  | Mackawy (2014) (151) | Asians | T2DM | DN | 40 | pers. micr/ria | 40 | DM>10 yrs, pers. norm/ria | 40 | non-diabetics |  |  | DC-C, HT-DC-C, HT-C |
|  | Huo (2015) (142) | E. Asians | T2DM | DN | 431 | urinary albumin>500 mg/L and ACR>300 mg/g | 420 | negative dipstick yrinary protein and urinary albumin<10 mg/L |  |  |  |  | DC-C |
|  | Narne (2014) (137) | Asians | T2DM | DN | 155 | pers. macr/ria , DR | 162 | pers. norm/ria |  |  |  |  | DC-C |
|  | Nagase (2003) (139) | E. Asians | NR | d. ESRD | 71 | d. ESRD, under dialysis |  | - | 248 | non-diabetics | No | - | HT-C |
|  | McKnight (2010) (152) | Caucasians | T1DM | DN | 718 | pers. proteinuria, hypertension, retinopathy | 749 | DM>15 yrs, norm/ria |  |  |  |  | HT-C |
|  | Thaha (2008) (153) | Asians | T2DM | ESRD | 100 | d. ESRD |  | - | 100 | non-diabetics |  |  | HT-C |
|  | Yahya (2019) (154) | Asians | T2DM | DN-ESRD | 131 | DM≥10 yrs, pers. proteinuria or ESRD | 96 | DM≥10 yrs, pers. normoalbuminuria |  |  |  |  | DC-C |
|  | Yahya (2019) (154) | Asians | T2DM | DN-ESRD | 108 | DM≥10 yrs, pers. proteinuria or ESRD | 95 | DM≥10 yrs, pers. normoalbuminuria |  |  |  |  | DC-C |
|  | Yahya (2019) (154) | Asians | T2DM | DN-ESRD | 86 | DM≥10 yrs, pers. proteinuria or ESRD | 136 | DM≥10 yrs, pers. normoalbuminuria |  |  |  |  | DC-C |
|  | Moguib (2017) (145) | Africans | T2DM |  | 200 | pers. proteinuria |  |  | 100 | Healthy |  |  | DC-C |
|  | Raina (2021) cohort 1 (146) | Asians | T2DM | DN | 204 | DN | 257 | pers. norm/ria | 315 | Healthy |  |  | DC-C |
|  | Raina (2021) cohort 2 (146) | Asians | T2DM | DN | 150 | DN | 187 | pers. norm/ria | 200 | Healthy |  |  | DC-C |
|  |  |  |  |  |  |  |  |  |  |  |  |  |  |
| NOS3 rs869109213 | Raina cohort 1 (146) | Asians | T2DM | DN | 204 | DN | 257 | pers. norm/ria | 315 | Healthy |  |  | DC-C |
|  | Raina cohort 2 (146) | Asians | T2DM | DN | 150 | DN | 187 | pers. norm/ria | 200 | Healthy |  |  | DC-C |
|  |  |  |  |  |  |  |  |  |  |  |  |  |  |
| **PRKCB**  c.–1504C>T, c.–546C>G | Pettigrew (2008) (155) | Caucasians | T1DM |  |  |  |  |  |  |  |  |  |  |
|  | Araki (2003) (156) | Caucasians | T1DM | DN | 231 | DN | 220 | pers. norm/ria |  |  |  |  | DC-C |
|  |  |  |  |  |  |  |  |  |  |  |  |  |  |
| TNF  -308G>A  rs1800629 | Kung (2010) (107) | E. Asians | T2DM | DN | 24 | DN | 23 | normal renal funcrion, matched age, gender, ethnicity | 25 | Healthy, matched age, gender, ethnicity | No | No | DC-C, HT-DC-C, HT-C |
|  | Prasad (2007) (157) | Asians | Type 2 | diabetic CRI | 196 | AER≥200 mg/l, serum Cr.≥3 mg/dl | 225 | norm/ria , DM≥10 yrs |  |  |  |  | DC-C |
|  | Lindholm (2008) (158) | Caucasians | T1DM | DN | 113 | pers. macr/ria or micr/ria | 340 | pers. norm/ria , DM≥10yrs |  |  |  | No | DC-C |
|  | Lindholm (2008) (158) | Caucasians | T2DM | DN | 314 | pers. macr/ria or micr/ria | 442 | pers. norm/ria , DM≥10yrs |  |  |  |  | DC-C |
|  | Lindholm (2008) (158) | Caucasians | T1DM | DN | 113 | pers. macr/ria or micr/ria | 342 | pers. norm/ria , DM≥10yrs |  |  |  | No | DC-C |
|  | Lindholm (2008) (158) | Caucasians | T2DM | DN | 314 | pers. macr/ria or micr/ria | 438 | pers. norm/ria , DM≥10yrs |  |  |  |  | DC-C |
|  | Sikka (2014) (159) | Asians | T2DM | DN | 145 | d. ESRD | 152 | DM only matched gender, ethnicity | 203 | Healthy, matched gender, ethnicity |  |  | DC-C, HT-DC-C, HT-C |
|  | Dabhi (2015) (160) | Asians | T2DM | DN | 188 | pers. micr/ria or proteinuria | 214 | Norm/ria | 235 | healthy |  |  | DC-C, HT-DC-C, HT-C |
|  | [Buraczynska (2004)](http://www.ncbi.nlm.nih.gov/pubmed?term=Buraczynska%20K%5BAuthor%5D&cauthor=true&cauthor_uid=15600254) (161) | Caucasians | NR | DN | 37 | diabetics under dialysis |  |  | 115 | healthy |  |  | HT-C |
|  | Babel (2006) (109) | Caucasians | T2DM | ESRD | 44 | d. ESRD |  | - | 118 | healthy |  |  | HT-C |
|  | Umapathy (2018) (162) | Asians | T2DM | DN | 164 | DN | 196 | pers. norm/ria | 218 | healthy |  |  | DC-C, HT-DC-C, HT-C |
|  |  |  |  |  |  |  |  |  |  |  |  |  |  |
| TGFB1 T869C rs1800470 | El-Sherbini (2013) (163) | Africans | T2DM | DN | 49 | pers. macro/micr/ria | 50 | pers. norm/ria | 98 | Healthy, matched age, gender |  |  | DC-C, HT-DC-C, HT-C |
|  | Mou (2011) (164) | E. Asians | T2DM | DN | 180 | stage III, IV, and V | 180 | normal renal function |  |  |  | No | DC-C |
|  | Valladares-Salgado (2010) (165) | Mixed (Mexico) | T2DM | DN | 233 | albuminuria (stage II, III, IV, and V) | 206 | norm/ria |  |  |  |  | DC-C |
|  | Jahromi (2010) (166) | caucasians | T1DM | DN | 64 | pers. proteinuria | 60 | norm/ria | 229 | newborn babies | No |  | DC-C, HT-DC-C, HT-C |
|  | Ahluwalia (2009)-North India (167) | E. Asians | T2DM | DN | 240 | albuminuria | 255 | pers. norm/ria , matched age and ethnicity |  |  |  | No | DC-C |
|  | Ahluwalia (2009)-South India (167) | E. Asians | T2DM | DN | 96 | albuminuria | 92 | pers. norm/ria , matched age and ethnicity |  |  |  |  |  |
|  | Buraczynska (2007) (168) | caucasians | T2DM | DN | 245 | pers. macr/ria , on dialysis, DM> 10 yrs | 168 | uncomplicated, DM>10 yrs | 400 | healthy |  |  | DC-C, HT-DC-C, HT-C |
|  | McKnight (2007) (169) | Caucasians | T1DM | DN | 272 | proteinuria excluding micr/ria | 367 | norm/ria excluding micr/ria, DM≥15 yrs, matched DM duration |  |  |  |  | DC-C |
|  | Patel (2005) (170) | Caucasians | T1DM | DN | 420 | pers. proteinuria excluding micr/ria | 410 | norm/ria , insulin≥50 yrs |  |  |  |  | DC-C |
|  | Wong (2003) (171) | E. Asians | T2DM | DN | 58 | pers. micr/ria or macr/ria with or without renal impairment | 65 | pers. norm/ria , normal renal function, DM>10 yrs, matched DM duration, age, gender, retinopathy, BP, diabetic control | 90 | healthy |  | No | DC-C, HT-DC-C, HT-C |
|  | Ng (2003) (172) | Caucasians (eyr. Amer) | T1DM | DN | 298 | pers. proteinuria or d. ESRD | 263 | pers. norm/ria , DM≥15 yrs |  |  |  |  | DC-C |
|  | Raina (2015) (173) | E. Asians | T2DM | DN | 354 | ESRD | 444 | norm/ria | 515 | Healthy matched gender |  |  | DC-C, HT-DC-C, HT-C |
|  | Babel (2006) (109) | Caucasians | T2DM | ESRD | 44 | d. ESRD |  | - | 118 | healthy | No |  | HT-C |
|  |  |  |  |  |  |  |  |  |  |  |  |  |  |
| TGFB1 Arg25Pro rs1800471 | El-Sherbini (2013) (163) | Africans | T2DM | DN | 49 | pers. macro/micr/ria | 50 | pers. norm/ria | 98 | Healthy, matched age, gender |  |  | DC-C |
|  | Valladares-Salgado (2010) (165) | Mixed (Mexico) | T2DM | DN | 233 | albuminuria (stage II, III, IV, and V) | 206 | norm/ria |  |  |  |  | DC-C |
|  | McKnight (2007) (169) | Caucasians | T1DM | DN | 272 | DM≥10 yrs before the onset of proteinuria | 367 | DM≥15 yrs, norm/ria , no anti-HT meds matched for DM duration |  |  |  |  | DC-C |
|  | Patel (2005) (170) | Caucasians | T1DM | DN | 420 | pers. proteinuria, hypertension and DR | 410 | insulin≥50 yrs,  norm/ria |  |  |  |  | DC-C |
|  | Ng (2003) (172) | Caucasians | T1DM | DN | 298 | pers. proteinuria or d. ESRD | 263 | pers. norm/ria , DM≥15 yrs |  |  |  |  | DC-C |
|  |  |  |  |  |  |  |  |  |  |  |  |  |  |
| TGFB1  -800 (G>A) rs1800468 | Valladares-Salgado (2010) (165) | Mixed (Mexico) | T2DM | DN | 233 | albuminuria (stage II, III, IV, and V) | 206 | norm/ria |  |  |  |  | DC-C |
|  | Prasad (2007) (157) | Asians | Type 2 | diabetic CRI | 196 | AER≥200 mg/l, s. Cr.≥3 mg/dl | 225 | norm/ria , DM≥10 yrs |  |  |  |  | DC-C |
|  | McKnight (2007) (169) | Caucasians | T1DM | DN | 272 | DM≥10 yrs before the onset of proteinuria | 367 | DM≥15 yrs, norm/ria , no anti-HT meds matched for DM duration |  |  |  |  | DC-C |
|  | Ng (2003) (172) | Caucasians | T1DM | DN | 298 | pers. proteinuria or d. ESRD | 263 | pers. norm/ria , DM≥15 yrs |  |  |  |  | DC-C |
|  |  |  |  |  |  |  |  |  |  |  |  |  |  |
| TGFB1  -509 C>T  rs1800469 | Buraczynska (2007) (168) | Caucasians | T2DM | DN | 245 | pers. macr/ria , on dialysis, DM> 10 yrs | 168 | uncomplicated, DM>10 yrs | 400 | healthy |  |  | DC-C |
|  | Prasad (2007) (157) | Asians | T2DM | diabetic CRI | 196 | AER≥200 mg/l, s. Cr.≥3 mg/dl | 225 | norm/ria , DM≥10 yrs |  |  |  |  | DC-C |
|  | McKnight (2007) (169) | Caucasians | T1DM | DN | 272 | DM≥10 yrs before the onset of proteinuria | 367 | DM≥15 yrs, norm/ria , no anti-HT meds matched for DM duration |  |  |  |  | DC-C |
|  | Ng (2003) (172) | Caucasians | T1DM | DN | 298 | pers. proteinuria or d. ESRD | 263 | pers. norm/ria , DM≥15 yrs |  |  |  |  | DC-C |
|  | Raina (2015) (173) | E. Asians | T2DM | DN | 354 | ESRD | 444 | norm/ria | 515 | Healthy matched gender |  | No | DC-C |
|  |  |  |  |  |  |  |  |  |  |  |  |  |  |
| TGFB1 Thr263lle rs1800472 | Patel (2005) (170) | Caucasians | T1DM | DN | 420 | pers. macr/ria | 410 | pers. norm/ria |  |  |  |  | DC-C |
|  | Ng (2003) (172) | Caucasians | T1DM | DN | 298 | pers. macr/ria | 263 | norm/ria, DM≥15 yrs |  |  |  |  | DC-C |
|  | Pociot (1998) (174) | Caucasians | T1DM | DN | 137 | pers. macr/ria | 105 | pers. norm/ria |  |  |  |  | DC-C |
|  |  |  |  |  |  |  |  |  |  |  |  |  |  |
| TGFB1 rs8179181 | Tong (2008) (101) | Caucasians | T2DM | DN | 374 | ESRD | 239 | norm/ria , DM≥15 yrs, matched age, ethnicity |  |  |  |  | Alleles DC-C |
|  | Tregouet (2008) (89) | Caucasians | T1DM | DN | 489 | pers. macr/ria | 463 | pers. norm/ria , DM≥15 yrs |  |  |  |  | Alleles DC-C |
|  | Tregouet (2008) (89) | Caucasians | T1DM | DN | 387 | pers. macr/ria | 469 | pers. norm/ria , DM≥15 yrs |  |  |  |  | Alleles DC-C |
|  | Tregouet (2008) (89) | Caucasians | T1DM | DN | 300 | pers. macr/ria | 391 | pers. norm/ria , DM≥15 yrs |  |  |  |  | Alleles DC-C |
|  |  |  |  |  |  |  |  |  |  |  |  |  |  |
| **VEGFA** rs2010963 | Nikzamir (2012) (175) | Asians | T2DM | DN | 255 | pers. macr/ria | 235 | pers. norm/ria |  |  |  |  | DC-C |
|  | [McKnight (2007)](http://www.ncbi.nlm.nih.gov/pubmed?term=McKnight%20AJ%5BAuthor%5D&cauthor=true&cauthor_uid=17616354) (176) | Caucasians | T1DM | DN | 242 | pers. macr/ria | 301 | pers. norm/ria | 400 | Healthy controls |  |  | DC-C |
|  | Buraczynska (2007) (177) | Caucasians | T2DM | DN | 245 | pers. macr/ria | 181 | pers. norm/ria |  |  |  |  | DC-C |
|  | Luo (2019) (75) | Asians | T2DM | DN | 650 | pers. macr/ria | 580 | pers. norm/ria |  |  |  |  | DC-C |
|  |  |  |  |  |  |  |  |  |  |  |  |  |  |
| **VEGFA** rs699947 | McKnight (2007) (176) | Caucasians | T1DM | DN | 242 | DN | 301 | Diabetics without DN | 400 | Healthy controls |  |  | DC-C |
|  | Luo (2019) (75) | East Asians | T2DM | DN | 650 | DN | 580 | Diabetics without DN |  |  |  |  | DC-C |
|  |  |  |  |  |  |  |  |  |  |  |  |  |  |
| **VEGFA**  -1499 C>T rs833061 | [McKnight (2007)](http://www.ncbi.nlm.nih.gov/pubmed?term=McKnight%20AJ%5BAuthor%5D&cauthor=true&cauthor_uid=17616354) (176) | Caucasians | T1DM | DN | 242 | proteinuria>0.5g/24h, DM≥10 yrs and DR | 301 | DM≥15 yrs, norm/ria, no anti-HT meds | 400 | non-diabetics |  |  | DC-C |
|  | Tiwari (2009) (149) | Asian Indians | T2DM | Diabetic CRI | 90 | moderate CRI, pers. s. Cr≥2 mg/dl, DM≥2 yrs, DR | 75 | DM≥10 yrs and s. Cr<2 mg/dl |  |  |  |  | DC-C |
|  | Tiwari (2009) (149) | Asian Indians | T2DM | Diabetic CRI | 106 | moderate CRI, s. Cr≥2 mg/dl, DM≥2 yrs, DR | 149 | DM≥10 yrs and s. Cr<2 mg/dl |  |  |  |  | DC-C |
|  |  |  |  |  |  |  |  |  |  |  |  |  |  |
| **VEGFA**  -2549 I/D  rs35569394 | Yang (2003) (178) | Caucasians | T1DM | DN | 102 | DM≥10 yrs, pers. proteinuria, retinopathy, without hematuria | 66 | DM≥20 yrs without retinopathy or proteinuria | 141 | non-diabetics |  |  | DC-C, HT-DC-C, HT-C |
|  | Buraczynska (2007) (177) | Caucasians | T2DM | DN | 245 | pers. macr/ria of whom 43% with DR | 91 | DM≥10 yrs, no nephropathy | 493 | non-diabetics |  |  | DC-C, HT-DC-C, HT-C |
|  | Dabhi (2015) (179) | Asians | T2DM | DN | 102 | pers. micr/ria or proteinuria | 103 | diabetics with norm/ria | 143 | non-diabetics |  |  | DC-C, HT-DC-C, HT-C |
|  | Amle (2015) (180) | Asians | T2DM | DN | 40 | macr/ria | 40 | only diabetics | 40 | non-diabetics matched for age, gender |  |  | DC-C, HT-DC-C, HT-C |

**References**

1. Wyawahare M, Neelamegam R, Vilvanathan S, Soundravally R, Das AK, Adithan C. Association of Angiotensin-Converting Enzyme Gene Polymorphisms and Nephropathy in Diabetic Patients at a Tertiary Care Centre in South India. Clinical medicine insights Endocrinology and diabetes [Internet]. 2017 Jan 29 [cited 2019 Jul 7];10:1179551417726779. Available from: http://journals.sagepub.com/doi/10.1177/1179551417726779

2. Mansouri M, Zniber A, Boualla L, El Badaoui G, Benkacem M, Rifai K, et al. Associations between clinical characteristics and angiotensin-converting enzyme gene insertion/deletion polymorphism in Moroccan population with Type-2 diabetic nephropathy. Saudi journal of kidney diseases and transplantation : an official publication of the Saudi Center for Organ Transplantation, Saudi Arabia. 2017;28(2):261–7.

3. Fawwaz S, Balbaa M, Fakhoury H, Borjac J, Fakhoury R. Association between angiotensin-converting enzyme insertion/deletion gene polymorphism and end-stage renal disease in lebanese patients with diabetic nephropathy. Saudi journal of kidney diseases and transplantation : an official publication of the Saudi Center for Organ Transplantation, Saudi Arabia. 2017;28(2):325–9.

4. Wang M, Zhang X, Song X, Zou X, Wu W, Wang Y, et al. Nodular glomerulosclerosis and renin angiotensin system in Chinese patients with type 2 diabetes. Molecular and cellular endocrinology. 2016 May;427:92–100.

5. Parchwani DN, Palandurkar KM, Hema Chandan Kumar D, Patel DJ. Genetic Predisposition to Diabetic Nephropathy: Evidence for a Role of ACE (I/D) Gene Polymorphism in Type 2 Diabetic Population from Kutch Region. Indian journal of clinical biochemistry : IJCB [Internet]. 2015 Jan [cited 2016 Sep 19];30(1):43–54. Available from: http://www.ncbi.nlm.nih.gov/pubmed/25646040

6. Fathi M, Nikzamir AR. Combination of Angiotensin Converting Enzyme Insertion / Deletion ( I / D ) ( rs4646994 ) and VEGF Polymorphism ( + 405G / C ; rs2010963 ) Synergistically Associated With the Development , of Albuminuria in Iranian Patients With Type 2 Diabetes Nakhjavani. 2015;17(2).

7. Parchwani DN, Kesari MG, Patel DD, Patel DJ. Influence of genetic variability at the ACE locus in intron 16 on Diabetic Nephropathy in T1DM patients. Indian Journal of Physiology and Pharmacology. 2014;58(4):327–37.

8. Ilić V, Ilić M, Soldatović I, Popović S, Magić Z. Association of renin-angiotensin system genes polymorphism with progression of diabetic nephropathy in patients with type 1 diabetes mellitus. Vojnosanitetski pregled. 2014 Jul;71(7):627–33.

9. Kumar R, Sharma RK, Agarwal S. Genetic predisposition for development of nephropathy in type 2 diabetes mellitus. Biochemical genetics [Internet]. 2013 Dec [cited 2014 Dec 4];51(11–12):865–75. Available from: http://www.ncbi.nlm.nih.gov/pubmed/23846111

10. El-Baz R, Settin A, Ismaeel A, Khaleel AA, Abbas T, Tolba W, et al. MTHFR C677T, A1298C and ACE I/D polymorphisms as risk factors for diabetic nephropathy among type 2 diabetic patients. Journal of the renin-angiotensin-aldosterone system : JRAAS [Internet]. 2012 Dec [cited 2014 Dec 4];13(4):472–7. Available from: http://www.ncbi.nlm.nih.gov/pubmed/22554825

11. Rahimi Z, Vaisi-Raygani A, Rahimi Z, Parsian A. Concomitant presence of endothelial nitric oxide 894T and angiotensin II-converting enzyme D alleles are associated with diabetic nephropathy in a Kurdish population from Western Iran. Nephrology (Carlton, Vic) [Internet]. 2012 Feb [cited 2014 Dec 4];17(2):175–81. Available from: http://www.ncbi.nlm.nih.gov/pubmed/22026967

12. Al-Harbi EM, Farid EM, Gumaa K a, Masuadi EM, Singh J. Angiotensin-converting enzyme gene polymorphisms and T2DM in a case-control association study of the Bahraini population. Molecular and cellular biochemistry [Internet]. 2011 Apr [cited 2014 Dec 4];350(1–2):119–25. Available from: http://www.ncbi.nlm.nih.gov/pubmed/21207118

13. Felehgari V, Rahimi Z, Mozafari H, Vaisi-Raygani A. ACE gene polymorphism and serum ACE activity in Iranians type II diabetic patients with macroalbuminuria. Molecular and cellular biochemistry. 2011 Jan;346(1–2):23–30.

14. Rahimi Z, Felehgari V, Rahimi M, Mozafari H, Yari K, Vaisi-Raygani A, et al. The frequency of factor V Leiden mutation, ACE gene polymorphism, serum ACE activity and response to ACE inhibitor and angiotensin II receptor antagonist drugs in Iranians type II diabetic patients with microalbuminuria. Molecular biology reports [Internet]. 2011 Mar [cited 2014 Dec 4];38(3):2117–23. Available from: http://www.ncbi.nlm.nih.gov/pubmed/20853144

15. Zsom M, Fülöp T, Zsom L, Baráth A, Maróti Z, Endreffy E. Genetic polymorphisms and the risk of progressive renal failure in elderly Hungarian patients. Hemodialysis international International Symposium on Home Hemodialysis [Internet]. 2011 Oct [cited 2014 Dec 4];15(4):501–8. Available from: http://www.ncbi.nlm.nih.gov/pubmed/22111818

16. Blech I, Katzenellenbogen M, Katzenellenbogen A, Wainstein J, Rubinstein A, Harman-Boehm I, et al. Predicting diabetic nephropathy using a multifactorial genetic model. PloS one. 2011;6(4):e18743.

17. Jayapalan JJ, Muniandy S, Chan SP. Null association between ACE gene I/D polymorphism and diabetic nephropathy among multiethnic Malaysian subjects. Indian journal of human genetics [Internet]. 2010 May [cited 2014 Dec 4];16(2):78–86. Available from: http://www.pubmedcentral.nih.gov/articlerender.fcgi?artid=2955956&tool=pmcentrez&rendertype=abstract

18. Currie D, McKnight a J, Patterson CC, Sadlier DM, Maxwell a P. Investigation of ACE, ACE2 and AGTR1 genes for association with nephropathy in Type 1 diabetes mellitus. Diabetic medicine : a journal of the British Diabetic Association [Internet]. 2010 Oct [cited 2014 Dec 4];27(10):1188–94. Available from: http://www.ncbi.nlm.nih.gov/pubmed/20854388

19. Palomo-Piñón S, Gutiérrez-Rodríguez ME, Díaz-Flores M, Sánchez-Barrera R, Valladares-Salgado A, Utrera-Barillas D, et al. DD genotype of angiotensin-converting enzyme in type 2 diabetes mellitus with renal disease in Mexican Mestizos. Nephrology (Carlton, Vic) [Internet]. 2009 Apr [cited 2014 Dec 4];14(2):235–9. Available from: http://www.ncbi.nlm.nih.gov/pubmed/19207872

20. Ahluwalia TS, Ahuja M, Rai TS, Kohli HS, Bhansali A, Sud K, et al. ACE variants interact with the RAS pathway to confer risk and protection against type 2 diabetic nephropathy. DNA and Cell Biology. 2009;28(3):141–50.

21. Ezzidi I, Mtiraoui N, Kacem M. Identification of specific angiotensin‐converting enzyme variants and haplotypes that confer risk and protection against type 2 diabetic nephropathy. Diabetes/metabolism … [Internet]. 2009 [cited 2015 Apr 22];(September):717–24. Available from: http://onlinelibrary.wiley.com/doi/10.1002/dmrr.1006/full

22. Nikzamir A, Esteghamati A, Feghhi M, Nakhjavani M, Rashidi A, Reza JZ. The insertion/deletion polymorphism of the angiotensin-converting enzyme gene is associated with progression, but not development, of albuminuria in Iranian patients with type 2 diabetes. Journal of the renin-angiotensin-aldosterone system : JRAAS. 2009;10(2):109–14.

23. Naresh VVS, Reddy a LK, Sivaramakrishna G, Sharma PVGK, Vardhan R V, Kumar VS. Angiotensin converting enzyme gene polymorphism in type II diabetics with nephropathy. Indian journal of nephrology. 2009;19(4):145–8.

24. Arfa I, Abid A, Nouira S, Elloumi-Zghal H, Malouche D, Mannai I, et al. Lack of association between the angiotensin-converting enzyme gene (I/D) polymorphism and diabetic nephropathy in Tunisian type 2 diabetic patients. Journal of the renin-angiotensin-aldosterone system : JRAAS [Internet]. 2008 Mar [cited 2014 Dec 4];9(1):32–6. Available from: http://www.ncbi.nlm.nih.gov/pubmed/18404607

25. Möllsten A, Kockum I, Svensson M, Rudberg S, Ugarph-Morawski A, Brismar K, et al. The effect of polymorphisms in the renin-angiotensin-aldosterone system on diabetic nephropathy risk. Journal of diabetes and its complications [Internet]. 2008 [cited 2014 Dec 4];22(6):377–83. Available from: http://www.ncbi.nlm.nih.gov/pubmed/18413189

26. Movva S, Alluri R V, Komandur S, Vattam K, Eppa K, Mukkavali KK, et al. Relationship of angiotensin-converting enzyme gene polymorphism with nephropathy associated with Type 2 diabetes mellitus in Asian Indians. Journal of diabetes and its complications [Internet]. 2007 [cited 2014 Dec 4];21(4):237–41. Available from: http://www.ncbi.nlm.nih.gov/pubmed/17616353

27. Hadjadj S, Tarnow L, Forsblom C, Kazeem G, Marre M, Groop P-H, et al. Association between angiotensin-converting enzyme gene polymorphisms and diabetic nephropathy: case-control, haplotype, and family-based study in three European populations. Journal of the American Society of Nephrology : JASN [Internet]. 2007 Apr [cited 2014 Nov 28];18(4):1284–91. Available from: http://www.ncbi.nlm.nih.gov/pubmed/17376814

28. Buraczynska M, Ksiazek P, Drop A, Zaluska W, Spasiewicz D, Ksiazek A. Genetic polymorphisms of the renin-angiotensin system in end-stage renal disease. Nephrology, dialysis, transplantation : official publication of the European Dialysis and Transplant Association - European Renal Association [Internet]. 2006 Apr [cited 2014 Dec 4];21(4):979–83. Available from: http://www.ncbi.nlm.nih.gov/pubmed/16384824

29. Lee SJ, Choi MG, Kim D-S, Kim TW. Manganese superoxide dismutase gene polymorphism (V16A) is associated with stages of albuminuria in Korean type 2 diabetic patients. Metabolism: clinical and experimental. 2006 Jan;55(1):1–7.

30. Ng DPK, Placha G, Choo S, Chia K, Warram JH, Krolewski AS. A disease haplotype for advanced nephropathy in type 2 diabetes at the ACE locus. Diabetes [Internet]. 2006 Sep [cited 2014 Dec 4];55(9):2660–3. Available from: http://www.ncbi.nlm.nih.gov/pubmed/16936219

31. Prasad P, Tiwari AK, Kumar KMP, Ammini a C, Gupta A, Gupta R, et al. Chronic renal insufficiency among Asian Indians with type 2 diabetes: I. Role of RAAS gene polymorphisms. BMC medical genetics [Internet]. 2006 Jan [cited 2014 Dec 4];7:42. Available from: http://www.pubmedcentral.nih.gov/articlerender.fcgi?artid=1479320&tool=pmcentrez&rendertype=abstract

32. Shestakova MV, Vikulova OK, Gorashko NM, Voronko OE, Babunova NB, Nosikov VV, et al. The relationship between genetic and haemodynamic factors in diabetic nephropathy (DN): Case–control study in type 1 diabetes mellitus (T1DM). Diabetes Research and Clinical Practice [Internet]. 2006 Nov [cited 2015 Apr 22];74(2):S41–50. Available from: http://linkinghub.elsevier.com/retrieve/pii/S0168822706002816

33. Park HC, Choi SR, Kim BS, Lee TH, Kang BS, Choi KH, et al. Polymorphism of the ACE gene in dialysis patients: Overexpression of DD genotype in type 2 diabetic end-stage renal failure patients. Yonsei Medical Journal. 2005;46(6):779–87.

34. Canani LH, Costa LA, Crispim D, Gonçalves Dos Santos K, Roisenberg I, Lisbôa HRK, et al. The presence of allele D of angiotensin-converting enzyme polymorphism is associated with diabetic nephropathy in patients with less than 10 years duration of Type 2 diabetes. Diabetic Medicine. 2005;22(9):1167–72.

35. Degirmenci I, Kebapci N, Basaran A, Efe B, Gunes H V, Akalin A, et al. Frequency of angiotensin-converting enzyme gene polymorphism in Turkish type 2 diabetic patients. International journal of clinical practice. 2005 Oct;59(10):1137–42.

36. Shin Shin Y, Baek SH, Chang KY, Park CW, Yang CW, Jin DC, et al. Relations between eNOS Glu298Asp polymorphism and progression of diabetic nephropathy. Diabetes research and clinical practice [Internet]. 2004 Sep [cited 2014 Dec 4];65(3):257–65. Available from: http://www.ncbi.nlm.nih.gov/pubmed/15331206

37. Arzu Ergen H, Hatemi H, Agachan B, Camlica H, Isbir T. Angiotensin-I converting enzyme gene polymorphism in Turkish type 2 diabetic patients. Experimental & molecular medicine. 2004 Aug;36(4):345–50.

38. Chang H-R, Cheng C-H, Shu K-H, Chen C-H, Lian J-D, Wu M-Y. Study of the polymorphism of angiotensinogen, anigiotensin-converting enzyme and angiotensin receptor in type II diabetes with end-stage renal disease in Taiwan. Journal of the Chinese Medical Association : JCMA. 2003 Jan;66(1):51–6.

39. De Cosmo S, Miscio G, Zucaro L, Margaglione M, Argiolas A, Thomas S, et al. The role of PC-1 and ACE genes in diabetic nephropathy in type 1 diabetic patients: evidence for a polygenic control of kidney disease progression. Nephrology, dialysis, transplantation : official publication of the European Dialysis and Transplant Association - European Renal Association. 2002;17(8):1402–7.

40. Fradin S, Goulet-Salmon B, Chantepie M, Grandhomme F, Morello R, Jauzac P, et al. Relationship between polymorphisms in the renin-angiotensin system and nephropathy in type 2 diabetic patients. Diabetes & metabolism. 2002 Feb;28(1):27–32.

41. Nakajima K, Tanaka Y, Nomiyama T, Ogihara T, Piao L, Sakai K, et al. Chemokine receptor genotype is associated with diabetic nephropathy in Japanese with type 2 diabetes. Diabetes. 2002 Jan;51(1):238–42.

42. Araz M, Yilmaz N, Gu K. Angiotensin-converting enzyme gene polymorphism and microvascular complications in Turkish type 2 diabetic patients. 2001;54:95–104.

43. Azar ST, Zalloua PA, Medlej R, Halabi G. The DD genotype of the ACE gene polymorphism is associated with diabetic nephropathy in the type-1 diabetics. Endocrine research. 2001;27(1–2):99–108.

44. Viswanathan V, Zhu Y, Bala K, Dunn S, Snehalatha C, Ramachandran A, et al. Association between ACE Gene Polymorphism and Diabetic Nephropathy in South Indian Patients. 2001;2(2):83–7.

45. Hadjadj S, Belloum R, Bouhanick B, Gallois Y, Guilloteau G, Chatellier G, et al. Prognostic value of angiotensin-I converting enzyme I/D polymorphism for nephropathy in type 1 diabetes mellitus: a prospective study. Journal of the American Society of Nephrology : JASN. 2001;12(3):541–9.

46. Thomas GN, Critchley JA, Tomlinson B, Lee ZS, Young RP, Cockran CS, et al. Albuminuria and the renin-angiotensin system gene polymorphisms in type-2-diabetic and in normoglycemic hypertensive Chinese. Clinical nephrology. 2001 Jan;55(1):7–15.

47. Gohda T, Makita Y, Shike T, Kobayashi M, Funabiki K, Haneda M, et al. Association of the DD genotype and development of Japanese type 2 diabetic nephropathy. Clinical nephrology. 2001 Dec;56(6):475–80.

48. Wu S, Xiang K, Zheng T, Sun D, Weng Q, Zhao H, et al. Relationship between the renin-angiotensin system genes and diabetic nephropathy in the Chinese. Chinese medical journal. 2000 May;113(5):437–41.

49. van Ittersum FJ, de Man AM, Thijssen S, de Knijff P, Slagboom E, Smulders Y, et al. Genetic polymorphisms of the renin-angiotensin system and complications of insulin-dependent diabetes mellitus. Nephrology, dialysis, transplantation : official publication of the European Dialysis and Transplant Association - European Renal Association. 2000 Jul;15(7):1000–7.

50. Hsieh MC, Lin SR, Hsieh TJ, Hsu CH, Chen HC, Shin SJ, et al. Increased frequency of angiotensin-converting enzyme DD genotype in patients with type 2 diabetes in Taiwan. Nephrology, dialysis, transplantation : official publication of the European Dialysis and Transplant Association - European Renal Association. 2000 Jul;15(7):1008–13.

51. De Cosmo S, Margaglione M, Tassi V, Garrubba M, Thomas S, Olivetti C, et al. ACE, PAI-1, decorin and Werner helicase genes are not associated with the development of renal disease in European patients with type 1 diabetes. Diabetes/metabolism research and reviews. 1999;15(4):247–53.

52. Miura J, Uchigata Y, Yokoyama H, Omori Y, Iwamoto Y. Genetic polymorphism of renin-angiotensin system is not associated with diabetic vascular complications in Japanese subjects with long-term insulin dependent diabetes mellitus. Diabetes Research and Clinical Practice. 1999;45(1):41–9.

53. Vleming LJ, van der Pijl JW, Lemkes HH, Westendorp RG, Maassen JA, Daha MR, et al. The DD genotype of the ACE gene polymorphism is associated with progression of diabetic nephropathy to end stage renal failure in IDDM. Clinical nephrology. 1999 Mar;51(3):133–40.

54. Tomino Y, Makita Y, Shike T, Gohda T, Haneda M, Kikkawa R, et al. Relationship between polymorphism in the angiotensinogen, angiotensin-converting enzyme or angiotensin II receptor and renal progression in Japanese NIDDM patients. Nephron. 1999 Jun;82(2):139–44.

55. Freire MB, van Dijk DJ, Erman A, Boner G, Warram JH, Krolewski AS. DNA polymorphisms in the ACE gene, serum ACE activity and the risk of nephropathy in insulin-dependent diabetes mellitus. Nephrology, dialysis, transplantation : official publication of the European Dialysis and Transplant Association - European Renal Association. 1998 Oct;13(10):2553–8.

56. Grzeszczak W, Zychma MJ, Lacka B, Zukowska-Szczechowska E. Angiotensin I-converting enzyme gene polymorphisms: relationship to nephropathy in patients with non-insulin dependent diabetes mellitus. Journal of the American Society of Nephrology : JASN. 1998 Sep;9(9):1664–9.

57. Young RP, Chan JC, Critchley JA, Poon E, Nicholls G, Cockram CS. Angiotensinogen T235 and ACE insertion/deletion polymorphisms associated with albuminuria in Chinese type 2 diabetic patients. Diabetes care. 1998 Mar;21(3):431–7.

58. Hanyu O, Hanawa H, Nakagawa O, Tani N, Andou N, Aizawa Y, et al. Polymorphism of the angiotensin I-converting enzyme gene in diabetic nephropathy in type II diabetic patients with proliferative retinopathy. Renal failure. 1998 Jan;20(1):125–33.

59. Huang XH, Rantalaiho V, Wirta O, Pasternack A, Hiltunen TP, Koivula T, et al. Angiotensin-converting enzyme insertion/deletion polymorphism and diabetic albuminuria in patients with NIDDM followed Up for 9 years. Nephron. 1998 Sep;80(1):17–24.

60. Kimura H, Gejyo F, Suzuki Y, Suzuki S. Polymorphisms of angiotensin converting enzyme and plasminogen activator inhibitor-1 genes in diabetes and macroangiopathy1. Kidney … [Internet]. 1998 [cited 2015 Apr 22];54:1659–69. Available from: http://www.nature.com/ki/journal/v54/n5/abs/4490422a.html

61. Ringel J, Beige J, Kunz R, Distler A, Sharma AM. Genetic variants of the renin-angiotensin system , diabetic nephropathy and hypertension. 1997;193–9.

62. Barnas U, Schmidt A, Illievich A, Kiener HP, Rabensteiner D, Kaider A, et al. Evaluation of risk factors for the development of nephropathy in patients with IDDM : insertion / deletion angiotensin converting enzyme gene polymorphism , hypertension and metabolic control. 1997;327–31.

63. Marre M, Jeunemaitre X, Gallois Y, Rodier M, Chatellier G, Sert C, et al. Contribution of genetic polymorphism in the renin-angiotensin system to the development of renal complications in insulin-dependent diabetes: Genetique de la Nephropathie Diabetique (GENEDIAB) study group. The Journal of clinical investigation. 1997 Apr;99(7):1585–95.

64. Schmidt S, Ritz E. Angiotensin I converting enzyme gene polymorphism and diabetic nephropathy in type II diabetes. Nephrology, dialysis, transplantation : official publication of the European Dialysis and Transplant Association - European Renal Association. 1997;12 Suppl 2:37–41.

65. Hibberd ML, Millward BA, Demaine AG. The angiotensin I-converting enzyme (ACE) locus is strongly associated with age and duration of diabetes in patients with type I diabetes. Journal of diabetes and its complications. 1997;11(1):2–8.

66. Ha SK, Seo JK. Insertion/deletion polymorphism in ACE gene as a predictor for progression of diabetic nephropathy. Kidney international Supplement. 1997 Sep;60:S28-32.

67. Chowdhury TA, Dronsfield MJ, Kumar S, Gough SL, Gibson SP, Khatoon A, et al. Examination of two genetic polymorphisms within the renin-angiotensin system: no evidence for an association with nephropathy in IDDM. Diabetologia. 1996 Sep;39(9):1108–14.

68. Nakajima S, Baba T, Yajima Y. Is ACE gene polymorphism a useful marker for diabetic albuminuria in Japanese NIDDM patients? Diabetes care. 1996 Dec;19(12):1420–2.

69. Ohno T, Kawazu S, Tomono S. Association analyses of the polymorphisms of angiotensin-converting enzyme and angiotensinogen genes with diabetic nephropathy in Japanese non-insulin-dependent diabetics. Metabolism: clinical and experimental. 1996 Feb;45(2):218–22.

70. Schmidt S, Schone N, Ritz E. Association of ACE gene polymorphism and diabetic nephropathy? The Diabetic Nephropathy Study Group. Kidney international. 1995 Apr;47(4):1176–81.

71. Mizuiri S, Hemmi H, Inoue A, Yoshikawa H, Tanegashima M, Fushimi T, et al. Angiotensin-converting enzyme polymorphism and development of diabetic nephropathy in non-insulin-dependent diabetes mellitus. Nephron. 1995;70(4):455–9.

72. Panagiotopoulos S, Smith TJ, Aldred GP, Baker EJ, Jacklin CJ, Jerums G. Angiotensin-converting enzyme (ACE) gene polymorphism in type II diabetic patients with increased albumin excretion rate. Journal of diabetes and its complications. 1995;9(4):272–6.

73. Tarnow L, Cambien F, Rossing P, Nielsen FS, Hansen B V, Lecerf L, et al. Lack of relationship between an insertion/deletion polymorphism in the angiotensin I-converting enzyme gene and diabetic nephropathy and proliferative retinopathy in IDDM patients. Diabetes. 1995 May;44(5):489–94.

74. Dudley CR, Keavney B, Stratton IM, Turner RC, Ratcliffe PJ. U.K. Prospective Diabetes Study. XV: Relationship of renin-angiotensin system gene polymorphisms with microalbuminuria in NIDDM. Kidney international. 1995 Dec;48(6):1907–11.

75. Luo Y, Luo J, Peng H. Associations Between Genetic Polymorphisms in the VEGFA, ACE, and SOD2 Genes and Susceptibility to Diabetic Nephropathy in the Han Chinese. Genetic testing and molecular biomarkers. 2019 Sep;23(9):644–51.

76. Osawa N, Koya D, Araki S, Uzu T, Tsunoda T, Kashiwagi A, et al. Combinational effect of genes for the renin-angiotensin system in conferring susceptibility to diabetic nephropathy. Journal of human genetics [Internet]. 2007 Jan [cited 2014 Dec 4];52(2):143–51. Available from: http://www.ncbi.nlm.nih.gov/pubmed/17143591

77. McDonough CW, Palmer ND, Hicks PJ, Roh BH, An SS, Cooke JN, et al. A genome-wide association study for diabetic nephropathy genes in African Americans. Kidney international. 2011 Mar;79(5):563–72.

78. Makuc J, Seruga M, Zavrsnik M, Cilensek I, Petrovic D. Angiotensinogen (AGT) gene missense polymorphisms (rs699 and rs4762) and diabetic nephropathy in Caucasians with type 2 diabetes mellitus. Bosnian journal of basic medical sciences. 2017 Aug;17(3):262–7.

79. Ilic V, Ilic M, Soldatovic I, Popovic S, Magic Z. Association of renin-angiotensin system genes polymorphism with progression of diabetic nephropathy in patients with type 1 diabetes mellitus. Vojnosanitetski pregled. 2014 Jul;71(7):627–33.

80. Reis KA, Ebinç FA, Koç E, Demirci H, Erten Y, Güz G, et al. Association of the angiotensinogen M235T and APO E gene polymorphisms in Turkish type 2 diabetic patients with and without nephropathy. Renal failure [Internet]. 2011 Jan [cited 2014 Dec 4];33(5):469–74. Available from: http://www.ncbi.nlm.nih.gov/pubmed/21500980

81. Mtiraoui N, Ezzidi I, Turki A, Chaieb M, Mahjoub T, Almawi WY. Renin-angiotensin-aldosterone system genotypes and haplotypes affect the susceptibility to nephropathy in type 2 diabetes patients. Journal of the renin-angiotensin-aldosterone system : JRAAS. 2011 Dec;12(4):572–80.

82. Chang H-R, Cheng C-H, Shu K-H, Chen C-H, Lian J-D, Wu M-Y. Study of the polymorphism of angiotensinogen, anigiotensin-converting enzyme and angiotensin receptor in type II diabetes with end-stage renal disease in Taiwan. Journal of the Chinese Medical Association : JCMA. 2003 Jan;66(1):51–6.

83. Zychma MJ, Zukowska-Szczechowska E, Lacka BI, Grzeszczak W. Angiotensinogen M235T and chymase gene CMA/B polymorphisms are not associated with nephropathy in type II diabetes. Nephrology, dialysis, transplantation : official publication of the European Dialysis and Transplant Association - European Renal Association. 2000 Dec;15(12):1965–70.

84. Freire MB, Ji L, Onuma T, Orban T, Warram JH, Krolewski AS. Gender-specific association of M235T polymorphism in angiotensinogen gene and diabetic nephropathy in NIDDM. Hypertension. 1998 Apr;31(4):896–9.

85. Schmidt S, Giessel R, Bergis KH, Strojek K, Grzeszczak W, Ganten D, et al. Angiotensinogen gene M235T polymorphism is not associated with diabetic nephropathy. The Diabetic Nephropathy Study Group. Nephrology, dialysis, transplantation : official publication of the European Dialysis and Transplant Association - European Renal Association. 1996 Sep;11(9):1755–61.

86. Fogarty DG, Harron JC, Hughes AE, Nevin NC, Doherty CC, Maxwell AP. A molecular variant of angiotensinogen is associated with diabetic nephropathy in IDDM. Diabetes. 1996 Sep;45(9):1204–8.

87. Tarnow L, Cambien F, Rossing P, Nielsen FS, Hansen B V, Ricard S, et al. Angiotensinogen gene polymorphisms in IDDM patients with diabetic nephropathy. Diabetes. 1996 Mar;45(3):367–9.

88. Vázquez-Moreno M, Locia-Morales D, Peralta-Romero J, Sharma T, Meyre D, Cruz M, et al. AGT rs4762 is associated with diastolic blood pressure in Mexicans with diabetic nephropathy. Journal of diabetes and its complications. 2021 Mar;35(3):107826.

89. Tregouet D-A, Groop P-H, McGinn S, Forsblom C, Hadjadj S, Marre M, et al. G/T substitution in intron 1 of the UNC13B gene is associated with increased risk of nephropathy in patients with type 1 diabetes. Diabetes. 2008 Oct;57(10):2843–50.

90. Ali Z, Kusrini I, Shahab A, Saleh I. Association between A1166C Polymorphism of the Angiotensin II Type-1 Receptor Gene and Type-2 Diabetic Nephropathy in an Indonesian Malay Population. Acta medica Indonesiana [Internet]. 2018 Oct [cited 2019 Jul 7];50(4):314–9. Available from: http://www.ncbi.nlm.nih.gov/pubmed/30630996

91. Moradi M, Rahimi Z, Amiri S, Rahimi Z, Vessal M, Nasri H. AT1R A1166C variants in patients with type 2 diabetes mellitus and diabetic nephropathy. Journal of nephropathology [Internet]. 2015;4(3):69–76. Available from: http://www.pubmedcentral.nih.gov/articlerender.fcgi?artid=4544557&tool=pmcentrez&rendertype=abstract

92. Shah VN, Cheema BS, Sharma R, Khullar M, Kohli HS, Ahluwalia TS, et al. ACACβ gene (rs2268388) and AGTR1 gene (rs5186) polymorphism and the risk of nephropathy in Asian Indian patients with type 2 diabetes. Molecular and cellular biochemistry [Internet]. 2013 Jan [cited 2014 Dec 4];372(1–2):191–8. Available from: http://www.ncbi.nlm.nih.gov/pubmed/23081748

93. Möllsten A, Vionnet N, Forsblom C, Parkkonen M, Tarnow L, Hadjadj S, et al. A polymorphism in the angiotensin II type 1 receptor gene has different effects on the risk of diabetic nephropathy in men and women. Molecular genetics and metabolism [Internet]. 2011 May [cited 2014 Dec 4];103(1):66–70. Available from: http://www.ncbi.nlm.nih.gov/pubmed/21316998

94. Mollsten A, Kockum I, Svensson M, Rudberg S, Ugarph-Morawski A, Brismar K, et al. The effect of polymorphisms in the renin-angiotensin-aldosterone system on diabetic nephropathy risk. Journal of diabetes and its complications. 2008;22(6):377–83.

95. Savage DA, Feeney SA, Fogarty DG, Maxwell AP. Risk of developing diabetic nephropathy is not associated with synergism between the angiotensin II (type 1) receptor C1166 allele and poor glycaemic control. Nephrology, dialysis, transplantation : official publication of the European Dialysis and Transplant Association - European Renal Association. 1999 Apr;14(4):891–4.

96. Doria A, Onuma T, Warram JH, Krolewski AS. Synergistic effect of angiotensin II type 1 receptor genotype and poor glycaemic control on risk of nephropathy in IDDM. Diabetologia. 1997 Nov;40(11):1293–9.

97. Chowdhury TA, Dyer PH, Kumar S, Gough SC, Gibson SP, Rowe BR, et al. Lack of association of angiotensin II type 1 receptor gene polymorphism with diabetic nephropathy in insulin-dependent diabetes mellitus. Diabetic medicine : a journal of the British Diabetic Association. 1997 Oct;14(10):837–40.

98. Tarnow L, Cambien F, Rossing P, Nielsen FS, Hansen B V, Ricard S, et al. Angiotensin-II type 1 receptor gene polymorphism and diabetic microangiopathy. Nephrology, dialysis, transplantation : official publication of the European Dialysis and Transplant Association - European Renal Association. 1996 Jun;11(6):1019–23.

99. Vionnet N, Tregouët D, Kazeem G, Gut I, Groop P-H, Tarnow L, et al. Analysis of 14 candidate genes for diabetic nephropathy on chromosome 3q in European populations: strongest evidence for association with a variant in the promoter region of the adiponectin gene. Diabetes [Internet]. 2006 Nov [cited 2014 Dec 4];55(11):3166–74. Available from: http://www.ncbi.nlm.nih.gov/pubmed/17065357

100. Fagerholm E, Ahlqvist E, Forsblom C, Sandholm N, Syreeni a, Parkkonen M, et al. SNP in the genome-wide association study hotspot on chromosome 9p21 confers susceptibility to diabetic nephropathy in type 1 diabetes. Diabetologia [Internet]. 2012 Sep [cited 2014 Dec 4];55(9):2386–93. Available from: http://www.ncbi.nlm.nih.gov/pubmed/22643932

101. Tong Z, Yang Z, Patel S, Chen H, Gibbs D, Yang X, et al. Promoter polymorphism of the erythropoietin gene in severe diabetic eye and kidney complications. 2008;

102. McKnight AJ, Patterson CC, Pettigrew K a, Savage D a, Kilner J, Murphy M, et al. A GREM1 gene variant associates with diabetic nephropathy. Journal of the American Society of Nephrology : JASN [Internet]. 2010 May [cited 2014 Nov 20];21(5):773–81. Available from: http://www.pubmedcentral.nih.gov/articlerender.fcgi?artid=2865734&tool=pmcentrez&rendertype=abstract

103. Buraczynska M, Ksiazek K, Wacinski P, Zaluska W. Interleukin-1β Gene (IL1B) Polymorphism and Risk of Developing Diabetic Nephropathy. Immunological investigations [Internet]. 2019 Aug 18 [cited 2019 Jul 7];48(6):577–84. Available from: https://www.tandfonline.com/doi/full/10.1080/08820139.2019.1595642

104. Stefanidis I, Kreuer K, Dardiotis E, Arampatzis S, Eleftheriadis T, Hadjigeorgiou GM, et al. Association between the interleukin-1β Gene (IL1B) C-511T polymorphism and the risk of diabetic nephropathy in type 2 diabetes: a candidate-gene association study. DNA and cell biology [Internet]. 2014 Jul [cited 2014 Dec 4];33(7):463–8. Available from: http://www.ncbi.nlm.nih.gov/pubmed/24839897

105. Lee SH, Ihm C-G, Sohn SD, Lee TW, Kim MJ, Koh G, et al. Polymorphisms in interleukin-1 beta and Interleukin-1 receptor antagonist genes are associated with kidney failure in Korean patients with type 2 diabetes mellitus. American journal of nephrology [Internet]. 2004 [cited 2014 Dec 4];24(4):410–4. Available from: http://www.ncbi.nlm.nih.gov/pubmed/15286433

106. Erdogan M, Cetinkalp S, Ozgen AG, Saygili F, Berdeli A, Yilmaz C. Interleukin-10 (-1082G/A) gene polymorphism in patients with type 2 diabetes with and without nephropathy. Genetic testing and molecular biomarkers. 2012 Feb;16(2):91–4.

107. Kung W-J, Lin C-C, Liu S-H, Chaung H-C. Association of interleukin-10 polymorphisms with cytokines in type 2 diabetic nephropathy. Diabetes technology & therapeutics [Internet]. 2010 Oct [cited 2014 Dec 4];12(10):809–13. Available from: http://www.ncbi.nlm.nih.gov/pubmed/20809684

108. Mtiraoui N. Predictive value of interleukin-10 promoter genotypes and haplotypes in determining the susceptibility to nephropathy in type 2 diabetes patients. 2009;(November 2008):57–63.

109. Babel N, Gabdrakhmanova L, Hammer MH, Schoenemann C, Skrypnikov V, Poliak N, et al. Predictive value of cytokine gene polymorphisms for the development of end-stage renal disease. Journal of nephrology. 2006;19(6):802–7.

110. Yin Q, Zhai Q, Wang D, Hai J, Cao M, Wang J, et al. Investigation on the association between inerleukin-10 -592C/A, 819C/T and -1082A/G gene polymorphisms and development of diabetic nephrophathy. International journal of clinical and experimental pathology. 2015;8(11):15216–21.

111. Polina ER, da Silva Pereira BL, Crispim D, Sbruzzi RC, Canani LH, dos Santos KG. Association of –1082A&amp;#x3e;G Polymorphism in the Interleukin-10 Gene with Estimated Glomerular Filtration Rate in Type 2 Diabetes. Kidney and Blood Pressure Research [Internet]. 2017 [cited 2019 Jul 6];42(6):1164–74. Available from: http://www.ncbi.nlm.nih.gov/pubmed/29227971

112. Arababadi MK, Reza Mirzaei M, Ali Sajadi SM, Hassanshahi G, Ahmadabadi BN, Salehabadi VA, et al. Interleukin (IL)-10 gene polymorphisms are associated with type 2 diabetes with and without nephropathy: a study of patients from the southeast region of Iran. Inflammation [Internet]. 2012 Jun [cited 2014 Dec 4];35(3):797–802. Available from: http://www.ncbi.nlm.nih.gov/pubmed/21909800

113. Yin Q, Zhai Q, Wang D, Hai J, Cao M, Wang J, et al. Investigation on the association between inerleukin-10 -592C/A, 819C/T and -1082A/G gene polymorphisms and development of diabetic nephrophathy. International journal of clinical and experimental pathology [Internet]. 2015 [cited 2019 Jul 7];8(11):15216–21. Available from: http://www.ncbi.nlm.nih.gov/pubmed/26823869

114. Loughrey B V, Maxwell AP, Fogarty DG, Middleton D, Harron JC, Patterson CC, et al. An interluekin 1B allele, which correlates with a high secretor phenotype, is associated with diabetic nephropathy. Cytokine. 1998 Dec;10(12):984–8.

115. Blakemore AI, Cox A, Gonzalez AM, Maskil JK, Hughes ME, Wilson RM, et al. Interleukin-1 receptor antagonist allele (IL1RN*2) associated with nephropathy in diabetes mellitus. Human genetics. 1996 Mar;97(3):369–74.

116. Abrahamian H, Endler G, Exner M, Mauler H, Raith M, Endler L, et al. Association of low-grade inflammation with nephropathy in type 2 diabetic patients: role of elevated CRP-levels and 2 different gene-polymorphisms of proinflammatory cytokines. Experimental and clinical endocrinology & diabetes : official journal, German Society of Endocrinology [and] German Diabetes Association [Internet]. 2007 Jan [cited 2014 Dec 4];115(1):38–41. Available from: http://www.ncbi.nlm.nih.gov/pubmed/17286233

117. Papaoikonomou S, Tentolouris N, Tousoulis D, Papadodiannis D, Miliou A, Papageorgiou N, et al. The association of the 174G>C polymorphism of interleukin 6 gene with diabetic nephropathy in patients with type 2 diabetes mellitus. Journal of diabetes and its complications [Internet]. 2013 [cited 2014 Dec 4];27(6):576–9. Available from: http://www.ncbi.nlm.nih.gov/pubmed/23871133

118. Ng DPK, Nurbaya S, Ye SHJ, Krolewski AS. An IL-6 haplotype on human chromosome 7p21 confers risk for impaired renal function in type 2 diabetic patients. Kidney international. 2008 Aug;74(4):521–7.

119. Germain M, Pezzolesi MG, Sandholm N, McKnight AJ, Susztak K, Lajer M, et al. SORBS1 gene, a new candidate for diabetic nephropathy: results from a multi-stage genome-wide association study in patients with type 1 diabetes. Diabetologia. 2015 Mar;58(3):543–8.

120. Ahluwalia TS, Khullar M, Ahuja M, Kohli HS, Bhansali A, Mohan V, et al. Common variants of inflammatory cytokine genes are associated with risk of nephropathy in type 2 diabetes among Asian Indians. PloS one [Internet]. 2009 Jan [cited 2015 Feb 3];4(4):e5168. Available from: http://www.pubmedcentral.nih.gov/articlerender.fcgi?artid=2663813&tool=pmcentrez&rendertype=abstract

121. Albert C, Kube J, Albert A, Schanze D, Zenker M, Mertens PR. Cubilin Single Nucleotide Polymorphism Variants are Associated with Macroangiopathy While a Matrix Metalloproteinase-9 Single Nucleotide Polymorphism Flip-Flop may Indicate Susceptibility of Diabetic Nephropathy in Type-2 Diabetic Patients. Nephron. 2019;141(3):156–65.

122. Brismar K, Gu HF. Original Article Genetic and Functional Analyses of MRAS and HNF1A Genes in Diabetes and Diabetic Nephropathy. 2012;127:121–7.

123. Liao L, Lim M-C, Chan S-W, Zhao J-J, Lee K-O. Nitric oxide synthase gene polymorphisms and nephropathy in Asians with Type 2 diabetes. Journal of diabetes and its complications [Internet]. 2006 [cited 2014 Dec 4];20(6):371–5. Available from: http://www.ncbi.nlm.nih.gov/pubmed/17070441

124. Rippin JD, Patel a, Belyaev ND, Gill G V, Barnett a H, Bain SC. Nitric oxide synthase gene polymorphisms and diabetic nephropathy. Diabetologia [Internet]. 2003 Mar [cited 2014 Dec 4];46(3):426–8. Available from: http://www.ncbi.nlm.nih.gov/pubmed/12687343

125. Rahimi Z, Rahimi Z, Shahvaisi-Zadeh F, Sadeghei S, Vessal M, Yavari N. eNOS 4a/b polymorphism and its interaction with eNOS G894T variants in type 2 diabetes mellitus: modifying the risk of diabetic nephropathy. Disease markers [Internet]. 2013 Jan [cited 2014 Dec 4];34(6):437–43. Available from: http://www.pubmedcentral.nih.gov/articlerender.fcgi?artid=3810362&tool=pmcentrez&rendertype=abstract

126. Shoukry A, Shalaby SM, Abdelazim S, Abdelazim M, Ramadan A, Ismail MI, et al. Endothelial nitric oxide synthase gene polymorphisms and the risk of diabetic nephropathy in type 2 diabetes mellitus. Genetic testing and molecular biomarkers. 2012 Jun;16(6):574–9.

127. Ahluwalia TS, Ahuja M, Rai TS, Kohli HS, Sud K, Bhansali A, et al. Endothelial nitric oxide synthase gene haplotypes and diabetic nephropathy among Asian Indians. Molecular and cellular biochemistry [Internet]. 2008 Jul [cited 2014 Dec 4];314(1–2):9–17. Available from: http://www.ncbi.nlm.nih.gov/pubmed/18401556

128. Ezzidi I, Mtiraoui N, Mohamed MBH, Mahjoub T, Kacem M, Almawi WY. Association of endothelial nitric oxide synthase Glu298Asp, 4b/a, and -786T>C gene variants with diabetic nephropathy. Journal of diabetes and its complications [Internet]. 2008 [cited 2014 Dec 4];22(5):331–8. Available from: http://www.ncbi.nlm.nih.gov/pubmed/18413207

129. Möllsten A, Wessman M, Svensson M, Forsblom C, Parkkonen M, Brismar K, et al. Glu298Asp and NOS4ab polymorphisms in diabetic nephropathy. Annals of medicine [Internet]. 2006 Jan [cited 2014 Dec 4];38(7):522–8. Available from: http://www.ncbi.nlm.nih.gov/pubmed/17101543

130. Shimizu T, Onuma T, Kawamori R, Makita Y, Tomino Y. Endothelial nitric oxide synthase gene and the development of diabetic nephropathy. Diabetes research and clinical practice. 2002 Dec;58(3):179–85.

131. Neugebauer S, Baba T, Watanabe T. Association of the nitric oxide synthase gene polymorphism with an increased risk for progression to diabetic nephropathy in type 2 diabetes. Diabetes. 2000 Mar;49(3):500–3.

132. Santos KG, Crispim D, Canani LH, Ferrugem PT, Gross JL, Roisenberg I. Association of eNOS gene polymorphisms with renal disease in Caucasians with type 2 diabetes. Diabetes research and clinical practice. 2011 Mar;91(3):353–62.

133. Degen B, Schmidt S, Ritz E. A polymorphism in the gene for the endothelial nitric oxide synthase and diabetic nephropathy. Vol. 16, Nephrology, dialysis, transplantation : official publication of the European Dialysis and Transplant Association - European Renal Association. England; 2001. p. 185.

134. Fujita H, Narita T, Meguro H, Ishii T, Hanyu O, Suzuki K, et al. Lack of association between an ecNOS gene polymorphism and diabetic nephropathy in type 2 diabetic patients with proliferative diabetic retinopathy. Hormone and metabolic research = Hormon- und Stoffwechselforschung = Hormones et metabolisme. 2000 Feb;32(2):80–3.

135. Lin S, Qu H, Qiu M. Allele A in intron 4 of ecNOS gene will not increase the risk of diabetic nephropathy in type 2 diabetes of Chinese population. Vol. 91, Nephron. Switzerland; 2002. p. 768.

136. Mehrab-Mohseni M, Tabatabaei-Malazy O, Hasani-Ranjbar S, Amiri P, Kouroshnia A, Bazzaz JT, et al. Endothelial nitric oxide synthase VNTR (intron 4 a/b) polymorphism association with type 2 diabetes and its chronic complications. Diabetes research and clinical practice. 2011 Mar;91(3):348–52.

137. Narne P, Ponnaluri KC, Siraj M, Ishaq M. Polymorphisms in oxidative stress pathway genes and risk of diabetic nephropathy in South Indian type 2 diabetic patients. Nephrology (Carlton, Vic). 2014 Oct;19(10):623–9.

138. Bellini MH, Figueira MN, Piccoli MF, Marumo JT, Cendoroglo MS, Neto MC, et al. Association of endothelial nitric oxide synthase gene intron 4 polymorphism with end-stage renal disease. Nephrology (Carlton, Vic). 2007 Jun;12(3):289–93.

139. Nagase S, Suzuki H, Wang Y, Kikuchi S, Hirayama A, Ueda A, et al. Association of ecNOS gene polymorphisms with end stage renal diseases. Molecular and cellular biochemistry. 2003 Feb;244(1–2):113–8.

140. Lamnissou K, Zirogiannis P, Trygonis S, Demetriou K, Pierides A, Koptides M, et al. Evidence for association of endothelial cell nitric oxide synthase gene polymorphism with earlier progression to end-stage renal disease in a cohort of Hellens from Greece and Cyprus. Genetic testing. 2004;8(3):319–24.

141. Asakimori Y, Yorioka N, Yamamoto I, Okumoto S, Doi S, Hirai T, et al. Endothelial nitric oxide synthase intron 4 polymorphism influences the progression of renal disease. Nephron. 2001 Oct;89(2):219–23.

142. Huo P, Zhang D, Guan X, Mei Y, Zheng H, Feng X. Association between genetic polymorphisms of ACE & eNOS and diabetic nephropathy. Molecular Biology Reports. 2015;42(1).

143. Bazzaz JT, Amoli MM, Pravica V, Chandrasecaran R, Boulton AJ, Larijani B, et al. eNOS gene polymorphism association with retinopathy in type 1 diabetes. Ophthalmic genetics [Internet]. 2010 Sep [cited 2015 Feb 13];31(3):103–7. Available from: http://www.ncbi.nlm.nih.gov/pubmed/20565248

144. Asakimori Y, Yorioka N, Taniguchi Y, Ito T, Ogata S, Kyuden Y, et al. T(-786)-->C polymorphism of the endothelial nitric oxide synthase gene influences the progression of renal disease. Nephron. 2002 Aug;91(4):747–51.

145. Moguib O, Raslan HM, Abdel Rasheed I, Effat L, Mohamed N, El Serougy S, et al. Endothelial nitric oxide synthase gene (T786C and G894T) polymorphisms in Egyptian patients with type 2 diabetes. Journal, genetic engineering & biotechnology. 2017 Dec;15(2):431–6.

146. Raina P, Sikka R, Gupta H, Matharoo K, Bali SK, Singh V, et al. Association of eNOS and MCP-1 Genetic Variants with Type 2 Diabetes and Diabetic Nephropathy Susceptibility: A Case-Control and Meta-Analysis Study. Biochemical genetics. 2021 Aug;59(4):966–96.

147. El-Din Bessa SS, Hamdy SM. Impact of nitric oxide synthase Glu298Asp polymorphism on the development of end-stage renal disease in type 2 diabetic Egyptian patients. Renal failure [Internet]. 2011 Jan [cited 2014 Dec 4];33(9):878–84. Available from: http://www.ncbi.nlm.nih.gov/pubmed/21854353

148. Möllsten A, Lajer M, Jorsal A, Tarnow L. The endothelial nitric oxide synthase gene and risk of diabetic nephropathy and development of cardiovascular disease in type 1 diabetes. Molecular genetics and metabolism [Internet]. 2009 May [cited 2014 Dec 4];97(1):80–4. Available from: http://www.ncbi.nlm.nih.gov/pubmed/19246226

149. Tiwari AK, Prasad P, B K T, Kumar KMP, Ammini a C, Gupta A, et al. Oxidative stress pathway genes and chronic renal insufficiency in Asian Indians with Type 2 diabetes. Journal of diabetes and its complications [Internet]. 2009 [cited 2014 Dec 4];23(2):102–11. Available from: http://www.ncbi.nlm.nih.gov/pubmed/18413200

150. Mollsten A, Wessman M, Svensson M, Forsblom C, Parkkonen M, Brismar K, et al. Glu298Asp and NOS4ab polymorphisms in diabetic nephropathy. Annals of medicine. 2006;38(7):522–8.

151. Mackawy AMH, Khan AA, Badawy ME-S. Association of the endothelial nitric oxide synthase gene G894T polymorphism with the risk of diabetic nephropathy in Qassim region, Saudi Arabia-A pilot study. Meta gene. 2014 Dec;2:392–402.

152. McKnight a J, Patterson CC, Sandholm N, Kilner J, Buckham T a, Parkkonen M, et al. Genetic polymorphisms in nitric oxide synthase 3 gene and implications for kidney disease: a meta-analysis. American journal of nephrology [Internet]. 2010 Jan [cited 2014 Dec 4];32(5):476–81. Available from: http://www.ncbi.nlm.nih.gov/pubmed/20962522

153. Thaha M, Pranawa, Yogiantoro M, Sutjipto, Sunarjo, Tanimoto M, et al. Association of endothelial nitric oxide synthase Glu298Asp polymorphism with end-stage renal disease. Clinical nephrology. 2008 Aug;70(2):144–54.

154. Yahya MJ, Ismail PB, Nordin NB, Akim ABM, Binti Md Yusuf WS, Adam NLB, et al. CNDP1, NOS3, and MnSOD Polymorphisms as Risk Factors for Diabetic Nephropathy among Type 2 Diabetic Patients in Malaysia. Journal of nutrition and metabolism. 2019;2019:8736215.

155. Pettigrew KA, McKnight AJ, Martin RJ, Patterson CC, Kilner J, Sadlier D, et al. No support for association of protein kinase C, beta 1 (PRKCB1) gene promoter polymorphisms c.-1504C>T and c.-546C>G with diabetic nephropathy in Type 1 diabetes. Vol. 25, Diabetic medicine : a journal of the British Diabetic Association. England; 2008. p. 1127–9.

156. Araki S-I, Ng DPK, Krolewski B, Wyrwicz L, Rogus JJ, Canani L, et al. Identification of a common risk haplotype for diabetic nephropathy at the protein kinase C-beta1 (PRKCB1) gene locus. Journal of the American Society of Nephrology : JASN. 2003 Aug;14(8):2015–24.

157. Prasad P, Tiwari AK, Kumar KMP, Ammini a C, Gupta A, Gupta R, et al. Association of TGFbeta1, TNFalpha, CCR2 and CCR5 gene polymorphisms in type-2 diabetes and renal insufficiency among Asian Indians. BMC medical genetics [Internet]. 2007 Jan [cited 2014 Dec 4];8:20. Available from: http://www.pubmedcentral.nih.gov/articlerender.fcgi?artid=1853079&tool=pmcentrez&rendertype=abstract

158. Lindholm E, Bakhtadze E, Cilio C, Agardh E, Groop L, Agardh C-D. Association between LTA, TNF and AGER polymorphisms and late diabetic complications. PloS one [Internet]. 2008 Jan [cited 2014 Dec 4];3(6):e2546. Available from: http://www.pubmedcentral.nih.gov/articlerender.fcgi?artid=2429972&tool=pmcentrez&rendertype=abstract

159. Sikka R, Raina P, Matharoo K, Bandesh K, Bhatia R, Chakrabarti S, et al. TNF-alpha (g.-308 G > A) and ADIPOQ (g. + 45 T > G) gene polymorphisms in type 2 diabetes and microvascular complications in the region of Punjab (North-West India). Current eye research. 2014 Oct;39(10):1042–51.

160. Dabhi B, Mistry KN. Oxidative stress and its association with TNF-alpha-308 G/C and IL-1alpha-889 C/T gene polymorphisms in patients with diabetes and diabetic nephropathy. Gene. 2015 May;562(2):197–202.

161. Buraczynska K, Koziol‐Montewka M, Majdan M, Tokarz A, Ksiazek A. Genetic Determination of TNF and Myeloperoxidase Production in Dialyzed Patients with Diabetic Nephropathy. Renal Failure [Internet]. 2004 Jan [cited 2014 Dec 4];26(6):633–9. Available from: http://informahealthcare.com/doi/abs/10.1081/JDI-200037165

162. Umapathy D, Krishnamoorthy E, Mariappanadar V, Viswanathan V, Ramkumar KM. Increased levels of circulating (TNF-α) is associated with (-308G/A) promoter polymorphism of TNF-α gene in Diabetic Nephropathy. International journal of biological macromolecules [Internet]. 2018 Feb [cited 2019 Jul 7];107(Pt B):2113–21. Available from: https://linkinghub.elsevier.com/retrieve/pii/S0141813017325916

163. El-Sherbini SM, Shahen SM, Mosaad YM, Abdelgawad MS, Talaat RM. Gene polymorphism of transforming growth factor-(beta)1 in Egyptian patients with type 2 diabetes and diabetic nephropathy. Acta Biochimica et Biophysica Sinica [Internet]. 2013;45(4):330–8. Available from: http://www.embase.com/search/results?subaction=viewrecord&from=export&id=L369148758%5Cnhttp://dx.doi.org/10.1093/abbs/gmt003%5Cnhttp://sfx.galib.uga.edu/sfx_emu1?sid=EMBASE&issn=16729145&id=doi:10.1093/abbs/gmt003&atitle=Gene+polymorphism+of+transforming+growth+factor-β1+in+Egyptian+patients+with+type+2+diabetes+and+diabetic+nephropathy&stitle=Acta+Biochim.+Biophys.+Sin.&title=Acta+Biochimica+et+Biophysica+Sinica&volume=45&issue=4&spage=330&epage=338&aulast=El-Sherbini&aufirst=Sherif+M.&auinit=S.M.&

164. Mou X, Liu W, Zhou D, Liu Y, Hu Y, Ma G, et al. Association of Chinese medicine constitution susceptibility to diabetic nephropathy and transforming growth factor-β1 (T869C) gene polymorphism. Chinese journal of integrative medicine [Internet]. 2011 Sep [cited 2014 Dec 4];17(9):680–4. Available from: http://www.ncbi.nlm.nih.gov/pubmed/21910069

165. Valladares-Salgado A, Angeles-Martínez J, Rosas M, García-Mena J, Utrera-Barillas D, Gómez-Díaz R, et al. Association of polymorphisms within the transforming growth factor-β1 gene with diabetic nephropathy and serum cholesterol and triglyceride concentrations. Nephrology (Carlton, Vic) [Internet]. 2010 Sep [cited 2014 Dec 4];15(6):644–8. Available from: http://www.ncbi.nlm.nih.gov/pubmed/20883286

166. Jahromi MM, Millward BA, Demaine AG. Significant correlation between association of polymorphism in codon 10 of transforming growth factor-beta1 T (29) C with type 1 diabetes and patients with nephropathy disorder. Journal of interferon & cytokine research : the official journal of the International Society for Interferon and Cytokine Research [Internet]. 2010 Feb [cited 2014 Dec 4];30(2):59–66. Available from: http://www.ncbi.nlm.nih.gov/pubmed/20039825

167. Ahluwalia TS, Khullar M, Ahuja M, Kohli HS, Bhansali A, Mohan V, et al. Common variants of inflammatory cytokine genes are associated with risk of nephropathy in type 2 diabetes among Asian Indians. PloS one [Internet]. 2009 Jan [cited 2014 Dec 4];4(4):e5168. Available from: http://www.pubmedcentral.nih.gov/articlerender.fcgi?artid=2663813&tool=pmcentrez&rendertype=abstract

168. Buraczynska M, Baranowicz-Gaszczyk I, Borowicz E, Ksiazek A. TGF-beta1 and TSC-22 gene polymorphisms and susceptibility to microvascular complications in type 2 diabetes. Nephron Physiology [Internet]. 2007 Jan [cited 2014 Dec 4];106(4):p69-75. Available from: http://www.ncbi.nlm.nih.gov/pubmed/17622752

169. McKnight AJ, Savage D a, Patterson CC, Sadlier D, Maxwell a P. Resequencing of genes for transforming growth factor beta1 (TGFB1) type 1 and 2 receptors (TGFBR1, TGFBR2), and association analysis of variants with diabetic nephropathy. BMC medical genetics [Internet]. 2007 Jan [cited 2014 Dec 4];8:5. Available from: http://www.pubmedcentral.nih.gov/articlerender.fcgi?artid=1808054&tool=pmcentrez&rendertype=abstract

170. Patel A, Scott WR, Lympany PA, Rippin JD, Gill G V, Barnett AH, et al. The TGF-beta 1 gene codon 10 polymorphism contributes to the genetic predisposition to nephropathy in Type 1 diabetes. Diabetic medicine : a journal of the British Diabetic Association. 2005 Jan;22(1):69–73.

171. Wong TYH, Poon P, Chow KM, Szeto CC, Cheung MK, Li PKT. Association of transforming growth factor-beta (TGF-beta) T869C (Leu 10Pro) gene polymorphisms with type 2 diabetic nephropathy in Chinese. Kidney international. 2003 May;63(5):1831–5.

172. Ng DPK, Warram JH, Krolewski AS. TGF-beta 1 as a genetic susceptibility locus for advanced diabetic nephropathy in type 1 diabetes mellitus: an investigation of multiple known DNA sequence variants. American journal of kidney diseases : the official journal of the National Kidney Foundation [Internet]. 2003 Jan [cited 2014 Dec 4];41(1):22–8. Available from: http://www.ncbi.nlm.nih.gov/pubmed/12500218

173. Raina P, Sikka R, Kaur R, Sokhi J, Matharoo K, Singh V, et al. Association of Transforming Growth Factor Beta-1 (TGF-β1) Genetic Variation with Type 2 Diabetes and End Stage Renal Disease in Two Large Population Samples from North India. Omics : a journal of integrative biology [Internet]. 2015;19(5):306–17. Available from: http://www.ncbi.nlm.nih.gov/pubmed/25871499

174. Pociot F, Hansen PM, Karlsen AE, Langdahl BL, Johannesen J, Nerup J. TGF-beta1 gene mutations in insulin-dependent diabetes mellitus and diabetic nephropathy. Journal of the American Society of Nephrology : JASN. 1998 Dec;9(12):2302–7.

175. Nikzamir A, Esteghamati A, Hammedian AA, Mahmoudi T. The role of vascular endothelial growth factor +405 G/C polymorphism and albuminuria in patients with type 2 diabetes mellitus. Molecular biology reports [Internet]. 2012 Feb [cited 2014 Dec 4];39(2):881–6. Available from: http://www.ncbi.nlm.nih.gov/pubmed/21562766

176. McKnight A-J, Maxwell a P, Patterson CC, Brady HR, Savage D a. Association of VEGF-1499C-->T polymorphism with diabetic nephropathy in type 1 diabetes mellitus. Journal of diabetes and its complications [Internet]. 2007 [cited 2014 Dec 4];21(4):242–5. Available from: http://www.ncbi.nlm.nih.gov/pubmed/17616354

177. Buraczynska M, Ksiazek P, Baranowicz-Gaszczyk I, Jozwiak L. Association of the VEGF gene polymorphism with diabetic retinopathy in type 2 diabetes patients. Nephrology, dialysis, transplantation : official publication of the European Dialysis and Transplant Association - European Renal Association. 2007 Mar;22(3):827–32.

178. Yang B, Cross DF, Ollerenshaw M, Millward BA, Demaine AG. Polymorphisms of the vascular endothelial growth factor and susceptibility to diabetic microvascular complications in patients with type 1 diabetes mellitus. 2003;17:1–6.

179. Dabhi B, Mistry KN, Patel H, Lal S. Vascular endothelial growth factor insertion/deletion gene polymorphism in West Indian patients of type 2 diabetes and diabetic nephropathy. Indian journal of biochemistry & biophysics. 2015 Apr;52(2):209–12.

180. Amle D, Mir R, Khaneja A, Agarwal S, Ahlawat R, Ray PC, et al. Association of 18bp insertion/deletion polymorphism, at -2549 position of VEGF gene, with diabetic nephropathy in type 2 diabetes mellitus patients of North Indian population. Journal of diabetes and metabolic disorders [Internet]. 2015;14:19. Available from: http://www.pubmedcentral.nih.gov/articlerender.fcgi?artid=4407794&tool=pmcentrez&rendertype=abstract
