# Supplementary material for: Key Genetic Components of Fibrosis in Diabetic Nephropathy: An Updated Systematic Review and Meta-Analysis
Source: Int J Mol Sci. 2022 Dec 5;23(23):15331. doi: 10.3390/ijms232315331 (PMC9736240; doi:10.3390/ijms232315331)
Supplement: Supplementary file 1 [file ijms-23-15331-s001.zip › Table Fibrosis only non sig status S10-S12.docx]

**Supplementary Table S10**: Results from meta-analyses of statistically significant polymorphisms listed in alphabetical order based on **genotype counts.**

| Diseased controls versus cases | | | | | | | | | | |  |
| --- | --- | --- | --- | --- | --- | --- | --- | --- | --- | --- | --- |
| **GENE** | **VARIANT** | **RS** | **Studies (n)** | **Cases/Controls (n)** | **RE OR_G_** | **95% LL** | **95% UL** | **I^2^(%)** | **P_Q_** | **P_E_** | **Current status** |
| ACE | T8968C | rs4311 | 3 | 1677/1519 | 0.92 | 0.69 | 1.24 | 81.28 | 0.00 | 0.38 | [13] |
| **AGT** | T174M | Rs4762 | 8 | 2070/1838 | 1.02 | 0.88 | 1.18 | 0.00 | 0.50 | 0.51 | Updated |
|  | All in HWE |  | 8 |  |  |  |  |  |  |  |  |
| AGTR1 | A1166C | rs5186 | 24 | 6000/5020 | 1.05 | 0.93 | 1.18 | 49.13 | 0.003 | 0.75 | [13] |
| AGTR1 | All in HWE |  | 21 | 5260/4296 | 0.97 | 0.87 | 1.08 | 49.13 | 0.13 | 0.57 | [13] |
| GREM1 |  | rs3207357 (T/C) | 2 | 862/936 | 1.32 | 0.86 | 2.04 | 71.85 | 0.05 | - | new |
| IL10 | -592 C>A | rs1800872 | 4 | 706/575 | 1.26 | 0.84 | 1.91 | 55.44 | 0.08 | 0.42 | updated |
| IL10 |  | All in HWE | 3 | 682/552 | 1.24 | 0.78 | 1.97 | 67.02 | 0.04 | 0.45 | updated |
| IL1RN | 86 bp VNTR IL1RN*1>2/3/4 | - | 3 | 135/341 | 2.16 | 1 | 4.65 | 70.99 | 0.03 | 0.13 | [13] |
| **IL6** | G(-174)C | rs1800795 | 3 |  | 1.11 | 0.50 | 2.46 | 78.03 | 0.01 | 0.53 | updated |
| MMP9 |  | rs17576 | 3 | 367/512 | 1.28 | 0.60 | 2.73 | 87.22 | <0.001 | 0.52 | [13] |
| MMP9 | All in HWE |  | 1 | 31/165 | 0.45 | 0.23 | 0.89 |  | 0.15 (SE) | na | [13] |
| MRAS |  | rs9818870 | 2 | 326/473 | 1.02 | 0.75 | 1.39 | 0.00 | 0.88 | - | new |
| NOS2 | (AAAT)4>5/6 |  | 4 | 520/453 | 0.96 | 0.66 | 1.40 | 0 | 0.91 | 0.23 | [13] |
| NOS2 | All in HWE |  | 0 |  |  |  |  |  |  | **na** | **[13]** |
| NOS3 | Intron 4 b>a | - | 17 | 3887/3196 | 1.09 | 0.96 | 1.23 | 24.04 | 0.18 | 0.17 | [13] |
| NOS3 | All in HWE |  | 16 | 3824/3130 | 1.04 | 0.94 | 1.15 | 0 | 0.73 | 0.28 | [13] |
| **PRKCB** | c.–1504C>T |  | 2 |  | 1.28 | 0.83 | 1.96 | 75.94 | 0.04 | na | Updated |
| **PRKCB** | c.–546C>G |  | 2 |  | 1.29 | 0.83 | 2.02 | 81.41 | 0.02 | na | Updated |
| **TNF** | -308G>A | rs1800629 | 9 | 1563/1855 | 1.07 | 0.88 | 1.29 | 21.09 | 0.26 | 0.12 | Updated |
|  | All in HWE |  | 4 | 722/867 | 0.97 | 0.76 | 1.25 | 4.84 | 0.37 | 0.15 |  |
| TGFB1 | T869C | rs1800470 | 11 | 2408/2452 | 1.16 | 0.94 | 1.44 | 75.49 | 0.00 | 0.31 | [13] |
| TGFB1 |  | All in HWE | 8 | 1930/1952 | 1.08 | 0.84 | 1.38 | 77.06 | 0.00 | 0.44 |  |
| TGFB1 | Arg25Pro | [rs1800471](http://www.pharmgkb.org/do/serve?rsid=rs1800471) | 5 | 1268/1188 | 1.09 | 0.79 | 1.51 | 36.7 | 0.18 | 0.1 | [13] |
|  |  | All in HWE | 5 |  |  |  |  |  |  |  |  |
| TGFB1 | -800 (G>A) | rs1800468 | 4 | 987/1056 | 1.1 | 0.87 | 1.39 | 0 | 0.67 | 0.33 | [13] |
|  |  | All in HWE | 4 |  |  |  |  |  |  |  |  |
| TGFB1 | -509 (C>T) | rs1800469 | 5 | 1362/1462 | 1.09 | 0.95 | 1.26 | 0 | 0.54 | 0.32 | [13] |
| TGFB1 | All in HWE |  | 4 | 1007/1018 | 1.03 | 0.87 | 1.22 | 0 | 0.63 | **0.003** |  |
| TGFB1 | Thr263lle | rs1800472 | 3 | 847/700 | 1.21 | 0.65 | 2.23 | 40.58 | 0.19 | 0.22 | [13] |
|  |  | All in HWE | 2 | 677/602 | 1.05 | 0.68 | 1.62 | 0.00 | 0.33 | - |  |
| **VEGFA** |  | rs2010963 | 4 | 1392/1207 | 1.01 | 0.73 | 1.40 | 79.81 | 0.01 | 0.41 | Updated |
|  |  | All in HWE | 4 |  |  |  |  |  |  |  |  |
| **VEGFA** |  | rs699947 | 2 | 892/881 | 1.32 | 0.96 | 1.82 | 68.90 | 0.07 | - | Updated |
| VEGFA | -1499 C>T | rs833061 | 3 | 435/524 | 1.28 | 0.63 | 2.57 | 88.86 | 0.00 | 0.16 | [13] |
|  |  | All in HWE | 3 |  |  |  |  |  |  |  |  |
| VEGFA | I/D -2549 | rs35569394 | 4 | 489/300 | 1.34 | 0.85 | 2.12 | 66.93 | 0.03 | 0.11 | [13] |
| VEGFA |  | All in HWE | 4 |  |  |  |  |  |  |  |  |
| **Healthy controls versus cases** | | | | | | | | | | |  |
| AGT | M235T | rs699 | 12 | 1399/1759 | 1.17 | 0.87 | 1.58 | 76.47 | 0 | 0.41 | [13] |
| AGT | All in HWE |  | 12 |  |  |  |  |  |  |  |  |
| AGT | C3889T (T174M) | rs4762 | 2 | 200/177 | 1.44 | 0.25 | 8.35 | 90.99 | 0 | - | [13] |
| AGTR1 | A1166C | rs5186 | 7 | 614/982 | 1.06 | 0.85 | 1.34 | 0 | 0.52 | 0.44 | [13] |
|  | All in HWE |  | 7 |  |  |  |  |  |  |  |  |
| **IL1B** | -511C>T | rs16944 | 2 | 601/715 | 0.93 | 0.33 | 2.62 | 87.15 | 0.01 | na | Updated |
|  | All in HWE |  | 2 |  |  |  |  |  |  |  |  |
| **IL6** | G(-174)C | rs1800795 | 3 | 665/805 | 1.31 | 0.62 | 2.76 | 80.02 | 0.01 | 0.12 | Updated |
|  | All in HWE |  | 2 | 642/635 | 0.86 | 0.62 | 1.18 | 0.00 | 0.33 | - |  |
| MRAS |  | rs9818870 | 2 | 326/668 | 0.99 | 0.73 | 1.34 | 0.00 | 0.91 | - | new |
| **TNF** | -308G>A | rs1800629 | 7 | 847/951 | 1.45 | 0.82 | 2.57 | 76.79 | <0.001 | 0.51 | updated |
|  | All in HWE |  | 4 | 414/667 | 1.01 | 0.66 | 1.53 | 41.08 | 0.17 | 0.42 |  |
| **Healthy controls versus diseased controls versus cases** | | | | | | | | | | |  |
| ACE | I>D |  | 21 | 2973/2622/3668 | 1.12 | 0.94 | 1.34 | 88.33 | 0 | 0.07 | [13] |
| ACE | All in HWE | I>D | 20 | 2566/2437/3436 | 1.13 | 0.94 | 1.37 | 88.32 | 0 | 0.07 | [13] |
| AGTR1 | A1166C | rs5186 | 4 | 236/391/370 | 1.12 | 0.8 | 1.56 | 18.12 | 0.3 | 0.49 | [13] |
| AGTR1 | All in HWE |  | 4 |  |  |  |  |  |  |  |  |
| IL10 | -592 C>A | rs1800872 | 3 | 639/525/873 | 0.94 | 0.43 | 2.02 | 92.24 | 0 | 0.39 | [13] |
| IL10 |  | All in HWE | 2 | 615/502/848 | 0.79 | 0.31 | 1.97 | 96.01 | 0 |  |  |
| IL10 | -1082 A>G | rs1800896 | 3 | 582/473/885 | 1.01 | 0.89 | 1.16 | 0 | 0.98 | 0.27 | [13] |
| IL10 |  | All in HWE | 1 | 515/402/748 | 1.02 | 0.89 | 1.17 | na | na | na |  |
| MRAS |  | rs9818870 | 2 | 326/473/668 | 0.98 | 0.81 | 1.18 | 0.00 | 0.72 | - | new |
| NOS3 | Intron 4 b>a | - | 7 | 1438/1088/1384 | 1.18 | 0.99 | 1.4 | 41.3 | 0.12 | 0.16 | [13] |
| NOS3 | All in HWE |  | 7 |  |  |  |  |  |  |  |  |
| **TNF** | -308G>A | rs1800629 | 5 | 766/638/723 | 1.38 | 0.86 | 2.22 | 80.90 | <0.001 | 0.51 | updated |
|  | All in HWE |  | 2 | 333/369/438 | 0.90 | 0.68 | 1.19 | 0 | 0.41 | na |  |
| **VEGFA** | I/D -2549 | rs35569394 | 4 | 489/300/817 | 1.11 | 0.83 | 1.47 | 69.3 | 0.02 | 0.1 | [13] |
| **VEGFA** |  | All in HWE | 4 |  |  |  |  |  |  |  |  |

## **Supplementary Table S11:** Results from meta-analyses of all eligible polymorphisms using **diseased controls versus cases** listed in alphabetical order based on **allele counts.**

| **GENE** | **VARIANT** | **RS** | **Studies (n)** | **Cases/Controls (n)** | **RE OR** | **95% LL** | **95% UL** | **I^^2^(%)** | **P_Q_** | **P_E_** | **Current status** |
| --- | --- | --- | --- | --- | --- | --- | --- | --- | --- | --- | --- |
| ACE | I>D |  | 7 | 1847/2402 | 1.113 | 0.982 | 1.261 | 41.078 | 0.117 | 0.26 | [13] |
| ACE | T8968C | rs4311 | 3 | 1042/1123 | 0.863 | 0.758 | 0.983 | 14.88 | 0.309 | 0.27 | [13] |
| ACE | T-3892C | rs1800764 | 3 | 1042/1123 | 1.145 | 0.944 | 1.388 | 60.66 | 0.079 | 0.08 | [13] |
| ACE |  | rs4366 | 3 | 1042/1123 | 1.122 | 0.921 | 1.367 |  |  | 0.18 | [13] |
| ACE |  | rs12449782 | 3 | 1042/1123 | 1.147 | 0.969 | 1.357 |  |  | 0.1 | [13] |
| AGT | M235T | rs699 | 6 | 2097/2371 | 1.038 | 0.935 | 1.153 | 24.73 | 0.249 | 0.49 | [13] |
| AGT |  | rs2478522 | 3 | 1176/1323 | 0.977 | 0.795 | 1.202 |  |  | 0.12 | [13] |
| AGT |  | rs3827749 | 3 | 1176/1323 | 1.038 | 0.903 | 1.194 |  |  | 0.35 | [13] |
| AGTR1 | A1166C | rs5186 | 3 | 1057/1127 | 1.00 | 0.862 | 1.161 |  |  | 0.45 | [13] |
| AGTR1 |  | rs1492078 | 3 | 1057/1127 | 1.076 | 0.950 | 1.219 |  |  | 0.26 | [13] |
| AGTR1 |  | rs275653 | 3 | 1057/1127 | 0.970 | 0.769 | 1.222 |  |  | 0.32 | [13] |
| AGTR1 |  | rs2276736 | 3 | 1057/1127 | 1.046 | 0.923 | 1.186 |  |  | 0.41 | [13] |
| AGTR1 |  | rs1800766 | 3 | 1057/1127 | 0.913 | 0.774 | 1.077 |  |  | 0.44 | [13] |
| AGTR1 |  | rs5182 | 3 | 1057/1127 | 0.947 | 0.840 | 1.068 |  |  | 0.08 | [13] |
| ANGPT1 |  | rs6469108 | 3 | 1176/1323 | 1.019 | 0.911 | 1.139 | 0 | 0.799 | 0.1 | [13] |
| ANGPT1 |  | ss95210423 | 3 | 1176/1323 | 0.915 | 0.786 | 1.064 | 12.319 | 0.320 | 0.33 | [13] |
| ANGPT1 |  | rs2507799 | 3 | 1176/1323 | 1.033 | 0.921 | 1.158 | 0 | 0.862 | 0.49 | [13] |
| ANGPT2 |  | ss95210423 | 3 | 1176/1323 | 1.062 | 0.841 | 1.341 | 0 | 0.575 | 0.18 | [13] |
| ANGPT2 |  | rs3020221 | 3 | 1176/1323 | 1.035 | 0.922 | 1.162 | 0 | 0.418 | 0.39 | [13] |
| ANGPT2 |  | rs1961222 | 3 | 1176/1323 | 1.045 | 0.928 | 1.177 | 0 | 0.577 | 0.44 | [13] |
| ANGPT2 |  | rs1982386 | 3 | 1176/1323 | 1.002 | 0.881 | 1.139 | 0 | 0.935 | 0.25 | [13] |
| ATF2 |  | rs2302663 | 3 | 1176/1323 | 1.234 | 0.810 | 1.879 | 66.74 | 0.049 | 0.31 | [13] |
| CDKN2A/B |  | rs10811661 | 4 | 2814/2716 | 1.081 | 0.864 | 1.351 | 76.76 | 0.005 | 0.19 | [13] |
| DGKA |  | rs7297871 | 3 | 1176/1323 | 1.005 | 0.851 | 1.187 | 28.025 | 0.249 | 0.51 | [13] |
| DGKA |  | rs2291615 | 3 | 1176/1323 | 0.995 | 0.878 | 1.129 | 0 | 0.403 | 0.12 | [13] |
| **EDN1** |  | **rs1794849** | **3** | **1176/1323** | **1.16** | **1.02** | **1.31** | **0** | **0.62** | **0.08** | **new** |
| EDN1 |  | rs2070699 | 3 | 1176/1323 | 1.085 | 0.917 | 1.283 | 54.823 | 0.109 | 0.22 | [13] |
| EDN1 |  | rs1800543 | 3 | 1176/1323 | 0.977 | 0.828 | 1.154 | 33.763 | 0.221 | 0.37 | [13] |
| EDN1 |  | rs5369 | 3 | 1176/1323 | 1.189 | 0.962 | 1.470 | 29.96 | 0.240 | 0.43 | [13] |
| EDN1 |  | rs1629862 | 3 | 1176/1323 | 1.197 | 0.973 | 1.472 | 26.087 | 0.258 | 0.42 | [13] |
| EGF |  | rs10002971 | 3 | 1176/1323 | 0.990 | 0.877 | 1.118 | 0 | 0.615 | 0.49 | [13] |
| EGFR |  | rs2072454 | 3 | 1176/1323 | 1.037 | 0.889 | 1.210 | 47.117 | 0.151 | 0.29 | [13] |
| EGFR |  | rs13222549 | 3 | 1176/1323 | 1.081 | 0.962 | 1.214 | 0 | 0.581 | 0.18 | [13] |
| EGFR |  | rs2692456 | 3 | 1176/1323 | 1.063 | 0.920 | 1.227 | 0 | 0.373 | 0.24 | [13] |
| FGF1 |  | rs34002 | 3 | 1176/1323 | 0.960 | 0.855 | 1.077 | 5.51 | 0.347 | 0.07 | [13] |
| FGFR1 |  | rs2280846 | 3 | 1176/1323 | 0.978 | 0.698 | 1.371 | 69.605 | 0.037 | 0.44 | [13] |
| FGFR1 |  | rs3925 | 3 | 1176/1323 | 1.026 | 0.896 | 1.174 | 0 | 0.463 | 0.12 | [13] |
| FGFR1 |  | rs4647905 | 3 | 1176/1323 | 1.007 | 0.878 | 1.156 | 0 | 0.577 | 0.03 | [13] |
| FGFR2 |  | rs2278202 | 3 | 1176/1323 | 1.028 | 0.919 | 1.150 | 0 | 0.619 | 0.39 | [13] |
| FGFR2 |  | rs3135810 | 3 | 1176/1323 | 0.976 | 0.842 | 1.131 | 0 | 0.617 | 0.42 | [13] |
| FGFR2 |  | rs1047100 | 3 | 1176/1323 | 0.960 | 0.843 | 1.095 | 0 | 0.742 | 0.3 | [13] |
| FLT1 |  | rs2296284 | 3 | 1176/1323 | 0.995 | 0.840 | 1.179 | 49.573 | 0.138 | 0.17 | [13] |
| FLT1 |  | rs3751395 | 3 | 1176/1323 | 1.072 | 0.958 | 1.200 | 0 | 0.617 | 0.41 | [13] |
| FLT1 |  | rs9551471 | 3 | 1176/1323 | 0.916 | 0.809 | 1.036 | 0 | 0.474 | 0 | [13] |
| **FLT4** |  | **rs2242221** | **3** | **1176/1323** | **1.14** | **1.01** | **1.29** | **0** | **0.38** | **0.43** | **new** |
| FLT4 |  | rs399108 | 3 | 1176/1323 | 1.108 | 0.972 | 1.261 | 25.828 | 0.260 | 0.4 | [13] |
| IGF1R |  | rs2229765 | 3 | 1176/1323 | 0.920 | 0.749 | 1.129 | 69.725 | 0.037 | 0.19 | [13] |
| IGF1R |  | rs2593053 | 3 | 1176/1323 | 1.003 | 0.883 | 1.139 | 18.074 | 0.295 | 0.21 | [13] |
| **IGF2/INS/TH cluster** |  | **rs1004446** | **3** | **1176/1323** | **1.16** | **1.03** | **1.31** | **0** | **0.49** | **0.22** | **new** |
| **IGF2/INS/TH cluster** |  | **rs4320932** | **3** | **1176/1323** | **0.84** | **0.73** | **0.96** | **0** | **0.43** | **0.06** | **new** |
| IGF2/INS/TH cluster |  | rs3213223 | 3 | 1176/1323 | 1.135 | 0.967 | 1.332 | 11.05 | 0.325 | 0.39 | [13] |
| IGF2/INS/TH cluster |  | rs3213225 | 3 | 1176/1323 | 0.959 | 0.856 | 1.075 | 0 | 0.776 | 0.18 | [13] |
| IGF2/INS/TH cluster |  | rs3213232 | 3 | 1176/1323 | 1.038 | 0.833 | 1.294 | 0 | 0.667 | 0.39 | [13] |
| IGF2/INS/TH cluster |  | rs680 | 3 | 1176/1323 | 0.919 | 0.810 | 1.042 | 0 | 0.866 | 0.05 | [13] |
| IGF2/INS/TH cluster |  | rs1003483 | 3 | 1176/1323 | 1.004 | 0.896 | 1.124 | 0 | 0.713 | 0.32 | [13] |
| IGF2/INS/TH cluster |  | rs2585 | 3 | 1176/1323 | 0.941 | 0.830 | 1.067 | 0 | 0.728 | 0.1 | [13] |
| IGF2/INS/TH cluster |  | rs3802971 | 3 | 1176/1323 | 1.008 | 0.812 | 1.251 | 29.416 | 0.243 | 0.004 | [13] |
| IGF2/INS/TH cluster |  | rs4244808 | 3 | 1176/1323 | 1.013 | 0.905 | 1.134 | 0 | 0.937 | 0.33 | [13] |
| IGF2/INS/TH cluster |  | rs689 | 4 | 2005/2227 | 1.188 | 0.999 | 1.413 | 48.778 | 0.119 | 0.22 | [13] |
| IGF2/INS/TH cluster |  | rs734351 | 3 | 1176/1323 | 0.948 | 0.846 | 1.063 | 0 | 0.780 | 0.49 | [13] |
| IGF2/INS/TH cluster |  | rs7924316 | 3 | 1176/1323 | 1.001 | 0.892 | 1.124 | 0 | 0.644 | 0.03 | [13] |
| IGF2/INS/TH cluster |  | rs3213232 | 3 | 1176/1323 | 1.038 | 0.833 | 1.294 | 0 | 0.667 | 0.39 | [13] |
| IGF2/INS/TH cluster |  | rs2070762 | 3 | 1176/1323 | 0.949 | 0.848 | 1.061 | 0 | 0.781 | 0.07 | [13] |
| IGF2/INS/TH cluster |  | rs7113485 | 3 | 1176/1323 | 0.929 | 0.821 | 1.052 | 0 | 0.926 | 0.11 | [13] |
| IL12A |  | rs583911 | 3 | 1057/1127 | 0.986 | 0.874 | 1.111 | 0 | 0.659 | 0 | [13] |
| IL12A |  | rs2243135 | 3 | 1057/1127 | 0.981 | 0.860 | 1.119 | 13.144 | 0.316 | 0.01 | [13] |
| IL12A |  | IL12A_6489 | 3 | 1057/1127 | 0.945 | 0.795 | 1.123 | 0 | 0.455 | 0.01 | [13] |
| IL12A |  | rs2243136 | 3 | 1057/1127 | 0.917 | 0.755 | 1.112 | 0 | 0.373 | 0.48 | [13] |
| IL12A |  | rs568408 | 3 | 1057/1127 | 0.961 | 0.811 | 1.138 | 0 | 0.972 | 0.47 | [13] |
| INPPL1 |  | rs2276047 | 3 | 1176/1323 | 1.053 | 0.921 | 1.204 | 0 | 0.576 | 0.5 | [13] |
| INPPL1 |  | rs2276048 | 3 | 1176/1323 | 1.019 | 0.877 | 1.184 | 12.527 | 0.319 | 0.37 | [13] |
| INPPL1 |  | rs9886 | 3 | 1176/1323 | 0.959 | 0.749 | 1.229 | 23.888 | 0.269 | 0.5 | [13] |
| ITPR3 |  | rs9368768 | 2 | 1503/1664 | 1.237 | 0.881 | 1.737 | 90.797 | <0.001 | - | [13] |
| NFATC1 |  | rs9518 | 3 | 1176/1323 | 1.234 | 0.998 | 1.525 | 55.355 | 0.106 | 0.03 | [13] |
| NFATC1 |  | rs16958940 | 3 | 1176/1323 | 0.930 | 0.722 | 1.199 | 49.345 | 0.139 | 0.18 | [13] |
| NFATC1 |  | rs8097537 | 3 | 1176/1323 | 1.108 | 0.946 | 1.297 | 47.79 | 0.147 | 0.35 | [13] |
| NFATC1 |  | rs9966033 | 3 | 1176/1323 | 1.070 | 0.864 | 1.327 | 41.905 | 0.179 | 0.33 | [13] |
| NFATC2 |  | rs2273642 | 3 | 1176/1323 | 0.949 | 0.748 | 1.204 | 70.889 | 0.032 | 0.21 | [13] |
| NFATC2 |  | rs6021231 | 3 | 1176/1323 | 1.047 | 0.926 | 1.182 | 12.037 | 0.321 | 0.06 | [13] |
| NFATC2 |  | rs228840 | 3 | 1176/1323 | 1.110 | 0.869 | 1.417 | 59.464 | 0.085 | 0.25 | [13] |
| NFATC3 |  | rs1073632 | 3 | 1176/1323 | 0.928 | 0.708 | 1.215 | 0 | 0.947 | 0.37 | [13] |
| NFATC3 |  | rs8060893 | 3 | 1176/1323 | 0.958 | 0.821 | 1.117 | 0 | 0.750 | 0.38 | [13] |
| NOS1 |  | rs3741476 | 3 | 1176/1323 | 0.936 | 0.599 | 1.463 | 89.811 | <0.001 | 0.42 | [13] |
| NOS1 |  | rs3741475 | 3 | 1176/1323 | 1.020 | 0.776 | 1.342 | 79.897 | 0.007 | 0.37 | [13] |
| PDGFB |  | rs9622979 | 3 | 1176/1323 | 0.929 | 0.690 | 1.251 | 33.945 | 0.220 | 0.15 | [13] |
| PDGFB |  | rs4821877 | 3 | 1176/1323 | 1.043 | 0.910 | 1.195 | 31.978 | 0.230 | 0.49 | [13] |
| PDGFB |  | rs2285100 | 3 | 1176/1323 | 0.922 | 0.824 | 1.031 | 0 | 0.809 | 0.36 | [13] |
| PDGFD |  | rs7103465 | 2 |  | 0.799 | 0.608 | 1.049 | 63.028 | 0.100 | na | [13] |
| PDGFRA |  | ss95210906 | 3 | 1176/1323 | 1.101 | 0.827 | 1.465 | 62.174 | 0.071 | 0.4 | [13] |
| PDGFRA |  | rs869978 | 3 | 1176/1323 | 1.046 | 0.768 | 1.424 | 80.079 | 0.007 | 0.36 | [13] |
| PDGFRA |  | rs12511976 | 3 | 1176/1323 | 1.058 | 0.870 | 1.288 | 0 | 0.986 | 0.17 | [13] |
| PDGFRB |  | rs740750 | 3 | 1176/1323 | 0.962 | 0.860 | 1.077 | 0 | 0.966 | 0.2 | [13] |
| PDGFRB |  | ss95212947 | 3 | 1176/1323 | 1.262 | 0.893 | 1.783 | 0 | 0.686 | 0.45 | [13] |
| PDGFRB |  | rs2304061 | 3 | 1176/1323 | 0.932 | 0.785 | 1.106 | 38.464 | 0.197 | 0.38 | [13] |
| PDGFRB |  | rs2302273 | 3 | 1176/1323 | 1.005 | 0.870 | 1.160 | 0 | 0.994 | 0.41 | [13] |
| RAP1B |  | rs3213921 | 3 | 1176/1323 | 0.969 | 0.861 | 1.092 | 0 | 0.569 | 0.21 | [13] |
| RELA |  | rs11227247 | 3 | 1176/1323 | 1.295 | 0.996 | 1.685 | 56.698 | 0.099 | 0.43 | [13] |
| RELA |  | rs11820062 | 3 | 1176/1323 | 1.011 | 0.850 | 1.204 | 58.41 | 0.090 | 0.27 | [13] |
| SMAD3 |  | rs2289791 | 3 | 1176/1323 | 1.008 | 0.883 | 1.150 | 0 | 0.654 | 0.33 | [13] |
| SMAD3 |  | rs11629568 | 3 | 1176/1323 | 0.929 | 0.755 | 1.145 | 70.242 | 0.035 | 0.12 | [13] |
| SMAD3 |  | rs2289261 | 3 | 1176/1323 | 0.993 | 0.855 | 1.153 | 39.509 | 0.191 | 0.22 | [13] |
| SMAD3 |  | ss95213933 | 3 | 1176/1323 | 0.868 | 0.578 | 1.302 | 58.175 | 0.092 | 0.43 | [13] |
| TGFB1 | C>T | rs8179181 | 4 | 1550/1562 | 1.13 | 0.96 | 1.34 | 0.47 |  | 0.47 | [13] |
| TGFB1 | C>T | rs1982073 | 3 | 1176/1323 | 1.03 | 0.92 | 1.16 | 0.13 |  | 0.13 | [13] |
| TGFB1 | G>T | rs2241717 | 3 | 1176/1323 | 0.96 | 0.83 | 1.11 | 0.43 |  | 0.43 | [13] |
| TGFBR1 | A>G | rs1571589 | 3 | 1176/1323 | 1.02 | 0.88 | 1.18 | 0.18 |  | 0.18 | [13] |
| TGFBR1 | A>G | rs928180 | 3 | 1176/1323 | 1.05 | 0.77 | 1.44 | 0.37 |  | 0.37 | [13] |
| THPO |  | rs956732 | 3 | 1057/1127 | 1.087 | 0.964 | 1.226 | 0 | 0.861 | 0.25 | [13] |
| THPO |  | rs6142 | 3 | 1057/1127 | 1.070 | 0.936 | 1.225 | 12.069 | 0.321 | 0.3 | [13] |
| THPO |  | rs6141 | 3 | 1057/1127 | 0.997 | 0.884 | 1.125 | 0 | 0.516 | 0.44 | [13] |
| VEGFA | C>A | rs2146323 | 3 | 1176/1323 | 0.85 | 0.76 | 0.95 | 0.2 |  | 0.2 | [13] |
| VEGFA | G>A | rs3024997 | 3 | 1176/1323 | **1.03** | **0.90** | **1.18** | 0.27 |  | 0.27 | [13] |
| VEGFA | C>T | rs3025000 | 3 | 1176/1323 | 1.01 | 0.89 | 1.14 | 0.18 |  | 0.18 | [13] |
| VEGFB | C>T | rs12366035 | 3 | 1176/1323 | 0.91 | 0.67 | 1.22 | 0.02 |  | 0.02 | [13] |
| VEGFC | G>A | rs585706 | 3 | 1176/1323 | 1.12 | 0.80 | 1.57 | 0.04 |  | 0.04 | [13] |

RS: SNP identifier, RE OR_G_: random effects odds ratio generalized, LL: lower limit, UL: upper limit, I^2^: I^2^ statistic, P_Q_: P-value from heterogeneity testing, P_E_: P-value from Egger’s Test

## **Supplementary table S12:** Results from meta-analyses of all eligible polymorphisms using **healthy controls versus cases** listed in alphabetical order based on **allele counts.**

| GENE | VARIANT | RS | Studies (n) | Cases/Controls (n) | RE OR | 95% LL | 95% UL | I^2(%) | P_Q_ | P_E_ | Current status |
| --- | --- | --- | --- | --- | --- | --- | --- | --- | --- | --- | --- |
| ACE | I>D |  | 3 | 328/788 | 1.084 | 0.895 | 1.312 | 0 | 0.21 | 0.46 | [13] |
| ADCY1 |  | rs17489793 | 2 | 1674/1719 | 0.743 | 0.471 | 1.174 | 78.948 | 0.029 | - | new |
| ADCY1 |  | rs34154333 | 2 | 1674/1719 | 0.755 | 0.535 | 1.063 | 74.43 | 0.048 | - | new |
| AGT | M235T | rs699 | 3 | 506/693 | 1.104 | 0.911 | 1.338 | 0 | 0.53 | 0.19 | [13] |
| AKT3 |  | rs1121276 | 2 | 1674/1719 | 0.850 | 0.585 | 1.233 | 87.724 | 0.004 | - | new |
| CACNA1E |  | rs11579714 | 2 | 1674/1719 | 0.841 | 0.675 | 1.048 | 52.505 | 0.147 | - | new |
| CACNG2 |  | rs11913713 | 2 | 1674/1719 | 1.193 | 0.915 | 1.555 | 78.516 | 0.031 | - | [13] |
| CALM2 |  | rs1027478 | 2 | 1674/1719 | 0.856 | 0.598 | 1.226 | 87.184 | 0.005 | - | new |
| FBN1 |  | rs16961261 | 2 | 1674/1719 | 0.808 | 0.498 | 1.313 | 83.053 | 0.015 | - | new |
| FBN1 |  | rs16961269 | 2 | 1674/1719 | 0.822 | 0.456 | 1.482 | 90.493 | 0.001 | - | new |
| IL12RB1 |  | rs372889 | 2 | 1674/1719 | 1.243 | 1.130 | 1.367 | 0 | 0.567 | - | new |
| KRAS |  | rs10842508 | 2 | 1674/1719 | 0.838 | 0.699 | 1.005 | 61.843 | 0.105 | - | new |
| MAPK10 |  | rs6827098 | 2 | 1674/1719 | 0.900 | 0.672 | 1.206 | 89.001 | 0.003 | - | new |
| MAPK10 |  | rs1460753 | 2 | 1674/1719 | 0.901 | 0.658 | 1.233 | 90.373 | 0.001 | - | new |
| ROR1 |  | rs7521581 | 2 | 1674/1719 | 1.179 | 0.838 | 1.659 | 74.84 | 0.05 | - | new |
| THSD4 |  | rs7174056 | 2 | 1674/1719 | 1.169 | 0.988 | 1.384 | 67.03 | 0.08 | - | new |
